# Dissection of the Transformation of Primary Human Hematopoietic Cells by the Oncogene NUP98-HOXA9

**DOI:** 10.1371/journal.pone.0006719

**Published:** 2009-08-21

**Authors:** Enas R. Yassin, Nayan J. Sarma, Anmaar M. Abdul-Nabi, James Dombrowski, Ye Han, Akiko Takeda, Nabeel R. Yaseen

**Affiliations:** 1 Department of Pathology and Immunology, Washington University School of Medicine, St. Louis, Missouri, United States of America; 2 Department of Pathology, Feinberg School of Medicine, Northwestern University, Chicago, Illinois, United States of America; KU Leuven, Belgium

## Abstract

NUP98-HOXA9 is the prototype of a group of oncoproteins associated with acute myeloid leukemia. It consists of an N-terminal portion of NUP98 fused to the homeodomain of HOXA9 and is believed to act as an aberrant transcription factor that binds DNA through the homeodomain. Here we show that NUP98-HOXA9 can regulate transcription without binding to DNA. In order to determine the relative contributions of the NUP98 and HOXA9 portions to the transforming ability of NUP98-HOXA9, the effects of NUP98-HOXA9 on primary human CD34+ cells were dissected and compared to those of wild-type HOXA9. In contrast to previous findings in mouse cells, HOXA9 had only mild effects on the differentiation and proliferation of primary human hematopoietic cells. The ability of NUP98-HOXA9 to disrupt the differentiation of primary human CD34+ cells was found to depend primarily on the NUP98 portion, whereas induction of long-term proliferation required both the NUP98 moiety and an intact homeodomain. Using oligonucleotide microarrays in primary human CD34+ cells, a group of genes was identified whose dysregulation by NUP98-HOXA9 is attributable primarily to the NUP98 portion. These include *RAP1A*, *HEY1*, and *PTGS2* (*COX-2*). Their functions may reflect the contribution of the NUP98 moiety of NUP98-HOXA9 to leukemic transformation. Taken together, these results suggest that the effects of NUP98-HOXA9 on gene transcription and cell transformation are mediated by at least two distinct mechanisms: one that involves promoter binding through the homeodomain with direct transcriptional activation, and another that depends predominantly on the NUP98 moiety and does not involve direct DNA binding.

## Introduction

NUP98-HOXA9 is an oncogenic fusion protein associated with acute myeloid leukemia (AML) that consists of an N-terminal, FG repeat-rich portion of the nucleoporin NUP98 fused to the homeodomain region of HOXA9 [1], [2]. It is the prototype of several similar leukemogenic fusions linking NUP98 to one of several homeodomain-containing transcription factors [3]. NUP98-HOX fusions act as aberrant transcription factors [4], [5], [6], [7], [8], [9], [10]. They induce proliferation and block differentiation in mouse hematopoietic precursors, and cause leukemia in mice [9], [10], [11], [12], [13], [14]. Some of these effects require a functional homeodomain [4], [6], [11], [12], [13]. In human primary hematopoietic cells, NUP98-HOXA9 induces long-term proliferation and blocks differentiation [15], [16]. It is not known whether these effects are dependent on homeodomain-DNA binding. Wild-type HOXA9 overexpression is leukemogenic in mice [12]. It induces proliferation with a differentiation block in mouse hematopoietic cells *in vitro*, and these effects are dependent on the presence of a functional homeodomain [17]. However, the effects of HOXA9 on the proliferation and differentiation of primary human hematopoietic cells have not been reported.

Several lines of evidence suggest that NUP98-HOXA9 may have effects over and above those mediated by the homeodomain. For example, in mice, the leukemia induced by NUP98-HOXA9 is preceded by a myeloproliferative phase whereas leukemia caused by overexpression of wild-type HOXA9 is not preceded by myeloproliferation [12]; and the *in vitro* effects of NUP98-HOXA9 on the differentiation and proliferation of mouse hematopoietic precursors are more profound than those of HOXA9 [13]. In addition, NUP98-HOXA9 modulates the expression of a larger set of genes than HOXA9 in a myeloid cell line [6]. Finally, while all leukemogenic NUP98 fusions contain an almost identical N-terminal portion of NUP98, most of them lack a homeodomain [18], suggesting that homeodomain-independent mechanisms may contribute to leukemogenesis by NUP98 chimeras.

In a recent study, a mutation that abolishes DNA binding (N51S) was introduced into the homeodomain of another leukemogenic NUP98 fusion, NUP98-HOXD13, and its effect on the gene expression profile in mouse hematopoietic cells was examined. It was found that the homeodomain mutant was still capable of dysregulating a subset of the genes dysregulated by NUP98-HOXD13 [19] indicating that some of the aberrant gene expression is independent of DNA binding by the homeodomain. However, it is not clear whether this homeodomain-independent dysregulation occurs at the transcriptional level and whether it has any functional significance in terms of leukemic transformation. These are among the questions we sought to answer in the current study. We first introduced the N51S mutation into NUP98-HOXA9 and showed that the mutant was capable of regulating transcription in a myeloid cell line without significant binding to DNA. We then compared this mutant to another mutant lacking the NUP98 portion as well as to intact NUP98-HOXA9 and wild-type HOXA9 for their ability to transform primary human CD34+ hematopoietic precursors. The results show that most of the effects of NUP98-HOXA9 on differentiation are dependent on the NUP98 portion and are not abolished by the N51S mutation. Gene expression profiling in primary human CD34+ cells identified a subset of the NUP98-HOXA9 target genes whose dysregulation requires the NUP98 moiety and appears to be independent of the homeodomain. The possible role of these genes in the transformation of primary human hematopoietic cells by NUP98-HOXA9 is discussed.

## Results

### Two distinct modes of transcriptional regulation by NUP98-HOXA9

**Figure 1:**
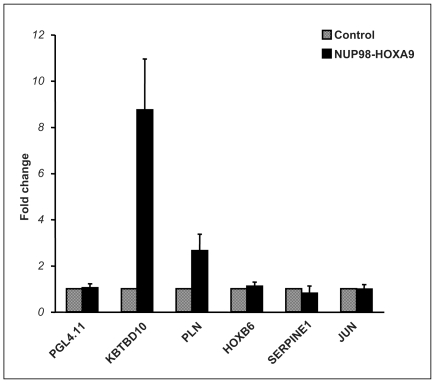
NUP98-HOXA9 regulates gene expression at the transcriptional level. K562 cells were transfected by electroporation with either empty pGL4.11 luciferase vector or with vector containing the promoter region of one of the five indicated genes (*KBTBD10*, kelch repeat and BTB (POZ) domain containing 10; *PLN*, phospholamban; *HOXB6*, homeobox B6; *SERPINE1*, serine proteinase inhibitor E1; and *JUN*, v-jun sarcoma virus 17 oncogene homolog) in combination with either empty pTracer/CMV-Bsd vector (Control) or vector expressing NUP98-HOXA9. Firefly luciferase activity was measured 48 h after transfection and normalized to a Renilla luciferase internal control. The numbers represent fold change over control (average of 3 independent experiments); error bars represent standard deviation.

To determine the role of homeodomain-DNA binding in the regulation of transcription by NUP98-HOXA9, luciferase reporter assays were carried out using the K562 human myeloid cell line. This cell line has been used successfully for analyzing transcriptional regulation by NUP98-HOXA9 [6], [20]. As the genes that NUP98-HOXA9 regulates in K562 cells are largely different from those it regulates in primary human CD34+ cells [6], [15], it was necessary to identify likely transcriptional targets of NUP98-HOXA9 in K562 cells. A microarray study was first undertaken to identify genes whose expression is modulated by NUP98-HOXA9 at an early time point (Table S1). The promoters of several of these genes were subcloned into the pGL4.11 vector upstream of luciferase. The luciferase reporter constructs were introduced into K562 cells along with a construct expressing NUP98-HOXA9. Two of these genes, *KBTBD10* and *PLN*, showed clear transactivation by NUP98-HOXA9 (Fig. 1).

**Figure 2:**
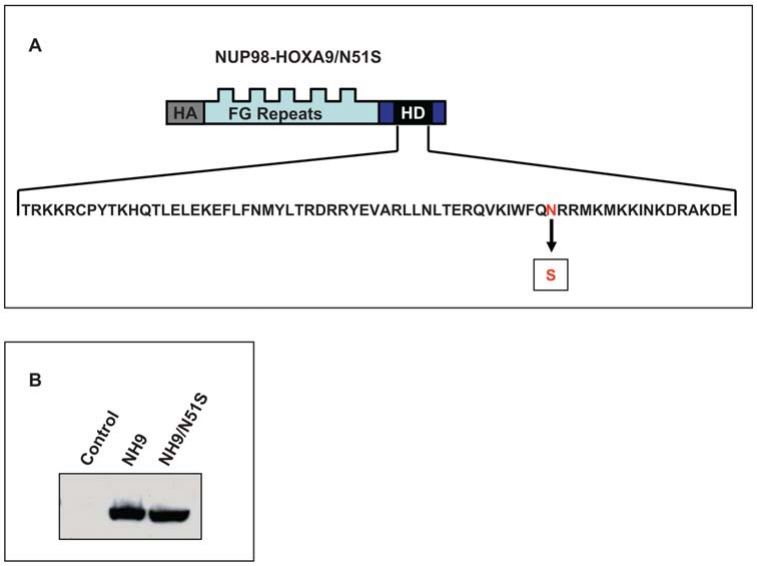
Expression of the NUP98-HOXA9/N51S mutant in K562 cells. (A) Schematic representation of the N51S mutation in the homeodomain of HA-tagged NUP98-HOXA9. FG Repeats: NUP98 FG repeat region; HD: HOXA9 homeodomain. (B) Immunoblotting using anti-HA antibody shows equivalent expression of NUP98-HOXA9 (NH9) and NUP98-HOXA9/N51S (NH9/N51S) in K562 cells.

**Figure 3:**
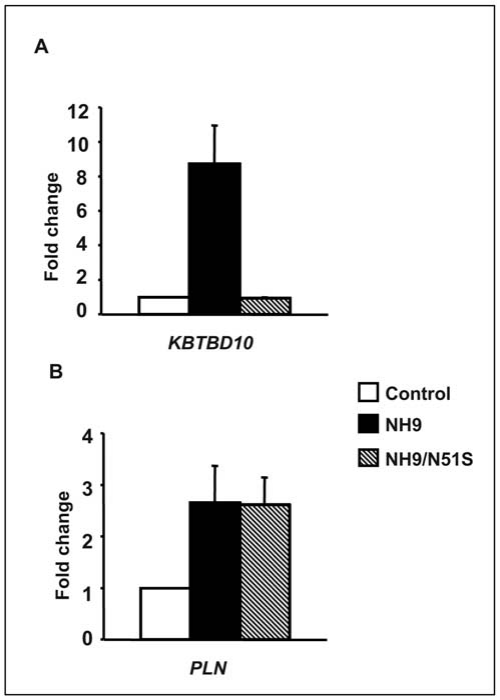
Transcriptional regulation by NUP98-HOXA9/N51S. (A) K562 cells were transfected by electroporation with a luciferase construct driven by the *KBTBD10* promoter and either empty pTracer/CMV-Bsd vector (Control) or vector expressing NUP98-HOXA9 (NH9) or NUP98-HOXA9/N51S (NH9/N51S). Firefly luciferase activity was measured 48 h after transfection and normalized to a Renilla luciferase internal control. The numbers represent fold change over control (average of 4 independent experiments); error bars represent standard deviation. (B) K562 cells were transfected by electroporation with a luciferase construct driven by the *PLN* promoter and either empty pTracer/CMV-Bsd vector (Control) or vector expressing NUP98-HOXA9 (NH9) or NUP98-HOXA9/N51S (NH9/N51S). Luciferase activity was measured and plotted as described in A above.

**Figure 4:**
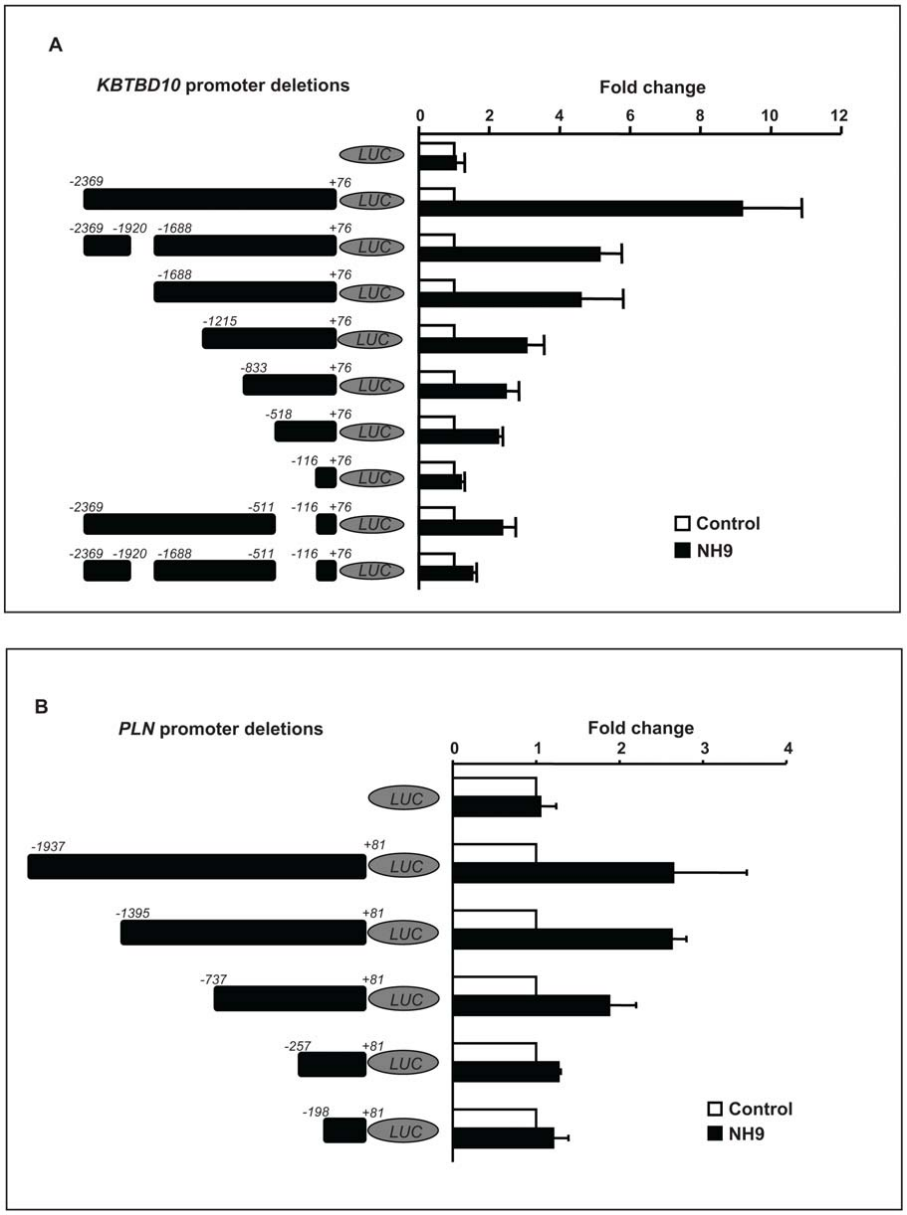
Identification of promoter sequences responsible for transactivation by NUP98-HOXA9. (A) The indicated segments of the *KBTBD10* promoter were subcloned into the pGL4.11 plasmid upstream of luciferase (LUC). K562 cells were transfected by electroporation with either empty pGL4.11 vector or with vector containing the indicated segments of the *KBTBD10* promoter along with either empty pTracer/CMV-Bsd vector (Control) or vector expressing NUP98-HOXA9 (NH9). Luciferase activity was measured and plotted as described in 3A above. The numbers represent the averages of 3 independent experiments. (B) Identification of *PLN* promoter sequences responsible for transactivation by NUP98-HOXA9. The indicated segments of the *PLN* promoter were subcloned into the pGL4.11 plasmid upstream of luciferase (LUC). K562 cells were transfected by electroporation with either empty pGL4.11 vector or with vector containing the indicated segments of the *PLN* promoter along with either empty pTracer/CMV-Bsd vector (Control) or vector expressing NUP98-HOXA9 (NH9). Luciferase activity was measured and plotted as described in 3A above. The numbers represent the averages of 3 independent experiments.

**Figure 5:**
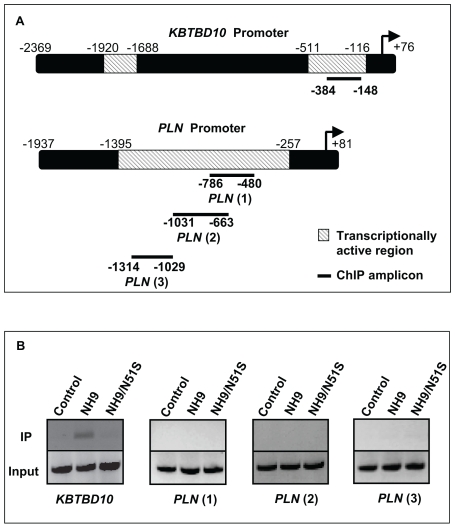
Chromatin immunoprecipitation with NUP98-HOXA9 and NUP98-HOXA9/N51S. (A) Schematic drawings of the *KBTBD10* and *PLN* promoters showing transcriptionally active regions identified in Fig. 4 above. The heavy lines indicate the segments amplified in the ChIP assay. (B) Chromatin was immunoprecipitated with anti-Flag antibody from K562 cells nucleofected with either pTracer/CMV-Bsd vector (control), or vector containing Flag-tagged NUP98-HOXA9 (NH9) or NUP98-HOXA9/N51S (NH9/N51S). Segments of the *KBTBD10* and *PLN* promoters (indicated in 5A above) were amplified by PCR. The upper lanes show immunoprecipitated chromatin (IP) and the lower lanes show input chromatin (Input).

To determine whether this transactivation is dependent on homeodomain-DNA binding, the N51S mutation was introduced into the homeodomain of NUP98-HOXA9 (Fig. 2A). The N51 residue of the homeodomain is very highly conserved among different homeobox proteins and is involved in direct homeodomain-DNA binding [21], [22]. Mutation of this residue has been well characterized and shown to abolish the ability of homeobox proteins to bind to DNA [10], [17], [19], [23]. The resulting mutant, NUP98-HOXA9/N51S, was expressed at a level equivalent to NUP98-HOXA9 in K562 cells (Fig. 2B). NUP98-HOXA9/N51S was tested for its effects on transcription from the *KBTBD10* and *PLN* promoters. NUP98-HOXA9/N51S did not transactivate the *KBTBD10* promoter (Fig. 3A); but surprisingly, it was able to transactivate the *PLN* promoter to the same extent as NUP98-HOXA9 (Fig. 3B). This suggests that NUP98-HOXA9 transactivates the *PLN* promoter by a mechanism that does not involve direct DNA binding. To confirm this, chromatin immunoprecipitation (ChIP) studies were carried out. First, deletion studies of the *KBTBD10* and *PLN* promoters were used to identify promoter regions responsible for transactivation by NUP98-HOXA9 (Fig. 4A, 4B, and 5A) and PCR primers were chosen for ChIP accordingly (Fig. 5A). Flag-tagged versions of NUP98-HOXA9 and NUP98-HOXA9/N51S were introduced into K562 cells by nucleofection. Chromatin was immunoprecipitated with anti-Flag antibody and the indicated segments of the *KBTBD10* and *PLN* promoters were amplified by PCR. NUP98-HOXA9 showed association with the *KBTBD10* promoter whereas NUP98-HOXA9/N51S did not show significant binding (Fig. 5B). Neither NUP98-HOXA9 nor NUP98-HOXA9/N51S showed significant binding to the segment of the *PLN* promoter that is transactivated by NUP98-HOXA9 (Fig. 5B). There may be very weak binding of NUP98-HOXA9 to the segment amplified by the PLN (3) primer set. However, unlike the binding to the *KBTBD10* promoter, this is difficult to distinguish from background and is not affected by the N51S mutation. Therefore it is unlikely to represent direct specific binding of the homeodomain to the promoter, but it could represent a very weak indirect interaction between NUP98-HOXA9 and the *PLN* promoter that is difficult to detect by our ChIP assays. Overall, the ChIP findings and the results of luciferase assays (Fig. 3A and 3B) show that transactivation of the *KBTBD10* promoter correlates with DNA binding whereas transactivation of the *PLN* promoter does not.

Taken together, these data suggest that NUP98-HOXA9 modulates transcription by at least two mechanisms: one, exemplified by the *KBTBD10* promoter, involves direct binding to the promoter through the homeodomain; while the other, exemplified by the *PLN* promoter, does not involve DNA binding.

### Disruption of the differentiation of primary human CD34+ cells by NUP98-HOXA9 does not require homeodomain-DNA binding

**Figure 6:**
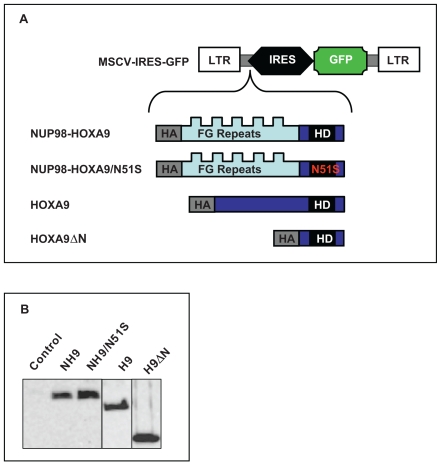
Retroviral transduction of primary human CD34+ cells. (A) HA-tagged NUP98-HOXA9, NUP98-HOXA9/N51S, HOXA9, or HOXA9ΔN open reading frames were subcloned into the MSCV-IRES-GFP vector. LTR: long terminal repeat; IRES: internal ribosomal entry site; FG Repeats: NUP98 FG repeat region; HD: HOXA9 homeodomain. (B) Primary human CD34+ cells were transduced with the retroviral vectors and sorted for GFP expression; protein expression was verified by immunoblotting with anti-HA antibody. NH9: NUP98-HOXA9; NH9/N51S: NUP98-HOXA9/N51S; H9: HOXA9; H9ΔN: HOXA9ΔN.

The data described above showed that NUP98-HOXA9 can disrupt gene expression by mechanisms that do not require DNA binding. It was not clear, however, whether such mechanisms contribute to leukemic transformation of human primary CD34+ cells by NUP98-HOXA9. We have previously shown that NUP98-HOXA9 interferes with both erythroid and myeloid differentiation of primary human CD34+ hematopoietic cells [15]. To determine the relative contributions of the NUP98 and HOXA9 moieties to disruption of hematopoietic differentiation of primary human CD34+ cells by NUP98-HOXA9, retroviral vectors were prepared that express NUP98-HOXA9, wild-type HOXA9, NUP98-HOXA9/N51S, and a mutant of NUP98-HOXA9 that lacks the NUP98 moiety (HOXA9ΔN) (Fig. 6A). All constructs expressed comparable protein levels in primary human CD34+ cells (Fig. 6B). The effects of NUP98-HOXA9, NUP98-HOXA9/N51S, HOXA9, and HOXA9ΔN on the differentiation of primary human CD34+ cells were compared to those of empty vector. Differentiation was assessed by colony-forming cell (CFC) assays combined with morphologic evaluation and flow cytometric immunophenotyping.

**Figure 7:**
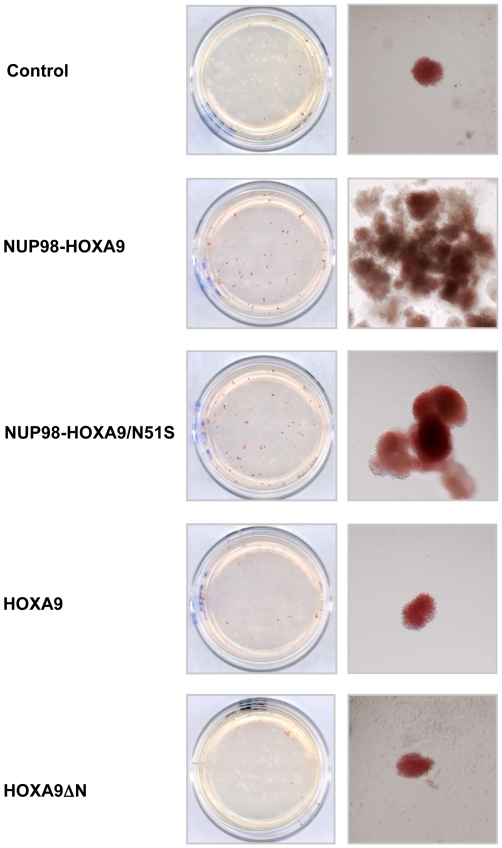
NUP98-HOXA9 and NUP98-HOXA9/N51S have similar effects on human CFC morphology. Primary human CD34+ cells were retrovirally transduced with either control MSCV-IRES-GFP vector or vector expressing NUP98-HOXA9, NUP98-HOXA9/N51S, HOXA9, or HOXA9ΔN, and cells were sorted for GFP positivity. One thousand cells were seeded into each of two duplicate plates for CFC assay and the experiment was repeated 3–4 independent times. Representative plates without magnification (left) and low power photomicrographs of representative erythroid colonies (right) are shown.

As previously reported [15], inspection of CFC plates without magnification showed many prominent large erythroid colonies in samples expressing NUP98-HOXA9 (Fig. 7). The large colonies included some with decreased hemoglobinization, indicating incomplete erythroid maturation. Cells expressing NUP98-HOXA9/N51S gave rise to a similar macroscopic appearance but with more fully hemoglobinized erythroid colonies (Fig. 7). In contrast, cells expressing either HOXA9 or HOXA9ΔN did not give rise to macroscopically prominent erythroid colonies (Fig. 7).

**Figure 8:**
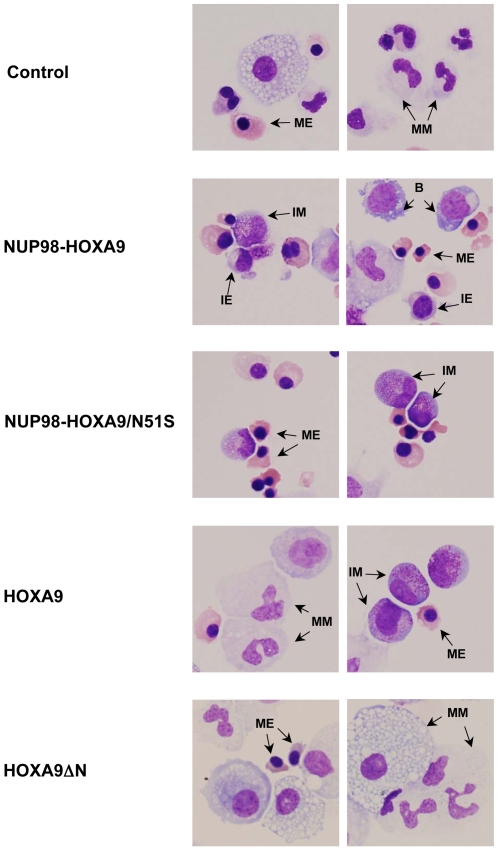
Cell morphology shows homeodomain-independent disruption of differentiation by NUP98-HOXA9. Cytospin smears were prepared from CFC plates (see Fig. 7 above) and stained with Giemsa. Photomicrographs were taken from representative fields with a 60×oil objective. For quantitation of the different cell types see Table 1. B: blast; MM: mature myeloid, IM: intermediate myeloid, ME: mature erythroid, IE: intermediate erythroid.

**Figure 9:**
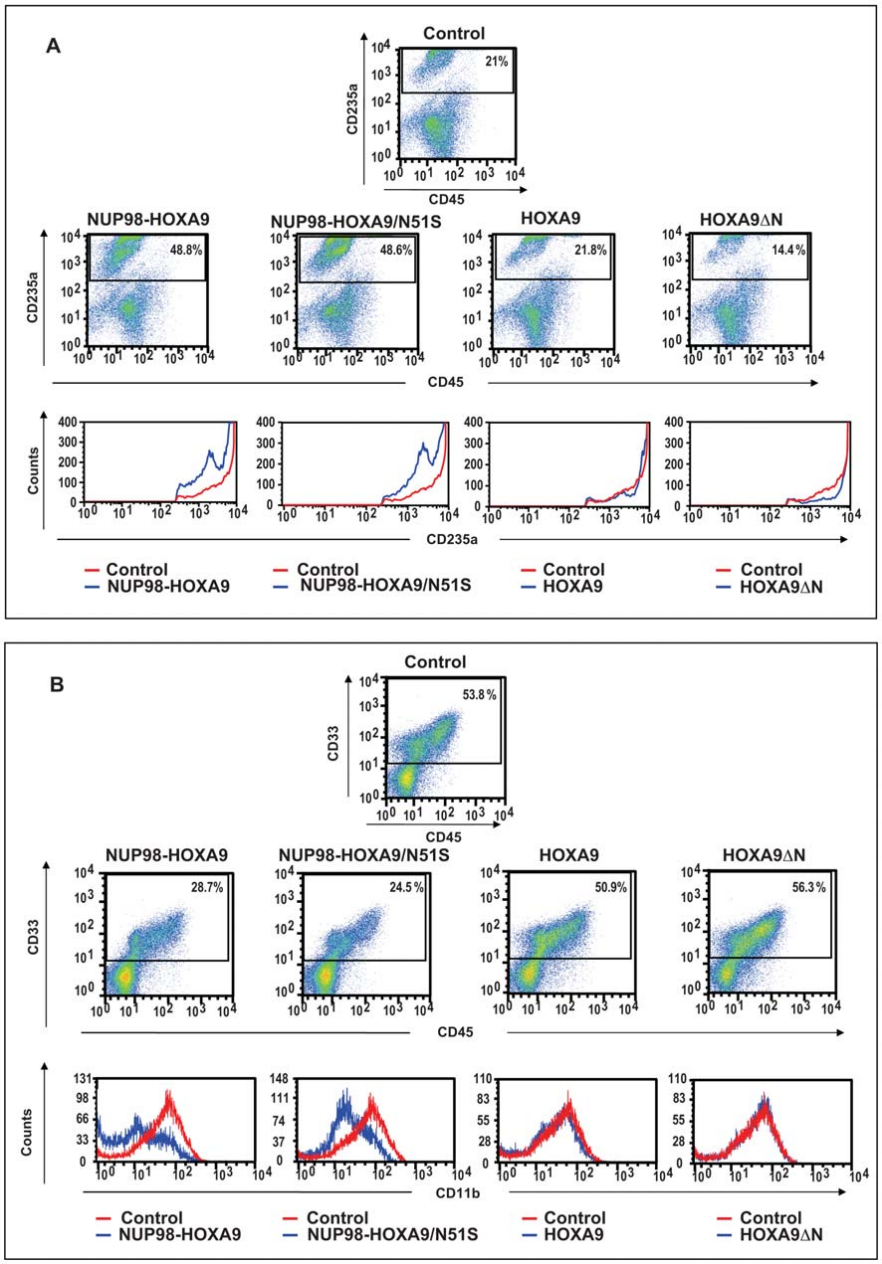
Flow cytometry shows homeodomain-independent disruption of human primary CD34+ cell differentiation by NUP98-HOXA9. (A) Flow cytometry for erythroid differentiation: Cells from the CFC plates (see Fig. 7 above) were harvested and stained with antibodies to CD45 and CD235a. The CD235a+ gate was plotted on a histogram (lower panels) to show the expression of CD235a relative to control cells. (B) Flow cytometry for myeloid differentiation: Cells from the CFC plates (see Fig. 7 above) were harvested and stained with CD45, CD33 and CD11b; the CD33+ gate was plotted on a histogram to show CD11b expression compared to control (lower panels). The percentages of cells falling within each gate are shown.

To further assess erythroid differentiation, cells were harvested from the CFC plates and subjected to morphologic evaluation on Giemsa-stained Cytospin preparations (Fig. 8) and to flow cytometric immunophenotyping (Fig. 9). The total number of cells per plate was increased in the NUP98-HOXA9 and the NUP98-HOXA9/N51S samples but not in the HOXA9 and HOXA9ΔN samples (Table 1). A 500-cell differential count was carried out on the Giemsa-stained slides from each sample; the average numbers from 3–4 independent experiments are shown in Table 1 and representative fields are shown in Fig. 8. There was a marked increase in the numbers of erythroid cells with a shift to immaturity in the NUP98-HOXA9 sample. Erythroid cells were also markedly increased in the NUP98-HOXA9/N51S sample, but without a statistically significant morphologic shift to immaturity. Erythroid cell number and morphology in the HOXA9 and HOXA9ΔN samples were not significantly different from the control sample.

**Table 1:** Dissection of the effects of NUP98-HOXA9 on hematopoietic differentiation based on cell morphology.

	Control	NUP98-HOXA9	NUP98-HOXA9/N51S	HOXA9	HOXA9ΔN
Primitive cells %	1.2±0.9	21.6±6.4**	5.2±3.0	7.0±3.7*	4.7±1.8
Intermediate erythroid %	2.8±0.8	16.9±2.1**	12.1±6.0	3.9±3.2	1.5±0.2
Mature erythroid %	23.7±7.9	33.8±10.2*	45.6±11.5*	14.3±12.5	6.5±2.4
Intermediate myeloid %	16.8±0.7	14.0±3.3	11.1±4.0*	33.6±7.2*	18.1±3.4
Mature myeloid %	55.6±6.2	13.7±3.9**	26.1±4.7**	41.3±6.4*	66.4±6.2
Total cells×10^6^	7.2±1.3	11.1±0.7**	9.7±2.4*	5.0±3.1	3.4±0.6

* *P*<0.05.

** *P*<0.01.

Averages from 3–4 independent experiments are shown; cells from 2 CFC plates for each experimental condition were harvested. The bottom line shows the average total cell numbers in the two plates±standard deviations. Cytospins smears were prepared and stained with Giemsa; a 500 cell differential count was performed. Cells with blast and promyelocyte morphology were counted as primitive; those with myelocyte/metamyelocyte morphology as intermediate myeloid; those with band, segmented neutrophil, monocyte, and macrophage morphology as mature myeloid; those with intermediate hemoglobinization as intermediate erythroid; and those with full hemoglobinization as mature erythroid. The first 5 rows show average percentages±standard deviations. The *P* value was obtained by comparing to control using a paired two-tailed distribution t-test.

To evaluate erythroid differentiation by flow cytometry, erythroid cells were gated based on CD235a (Glycophorin A) positivity, and plotted on a histogram to show the level of CD235a expression relative to control cells (Fig. 9A). The expression level of CD235a was decreased in cells expressing NUP98-HOXA9 and NUP98-HOXA9/N51S, indicating disrupted erythroid differentiation. In contrast, no significant reduction in CD235a expression was seen in cells expressing HOXA9 and HOXA9ΔN.

Myeloid differentiation was also assessed by morphologic evaluation of Giemsa-stained Cytospin preparations and flow cytometric immunophenotyping. The results of 500-cell differential counts are shown in Table 1 and representative fields from the Giemsa-stained slides are shown in Fig. 8. The percentage of mature myeloid cells was markedly decreased in the NUP98-HOXA9 samples, moderately decreased in the NUP98-HOXA9/N51S samples, and mildly decreased in the HOXA9 samples (Table 1). Primitive cells were markedly increased in the NUP98-HOXA9 samples and mildly increased in the HOXA9 samples. The latter also showed a significant increase in the numbers of myeloid cells with intermediate differentiation. NUP98-HOXA9/N51S caused a mild increase in the numbers of primitive cells, but the change did not reach statistical significance. HOXA9ΔN did not cause a significant change in myeloid cell morphology.

For flow cytometric evaluation of myeloid differentiation, myeloid cells (CD33+) were gated and the level of CD11b expression was compared to control cells on a histogram (Fig. 9B). Both NUP98-HOXA9 and NUP98-HOXA9/N51S caused a prominent decrease in the expression of CD11b, consistent with a block in myeloid differentiation. In contrast, HOXA9 and HOXA9ΔN did not significantly alter the expression of CD11b.

The effects of NUP98-HOXA9, NUP98-HOXA9/N51S, HOXA9, and HOXA9ΔN on the differentiation of primary human CD34+ cells are summarized in Table 2. The ability of NUP98-HOXA9/N51S to recapitulate most of the effects of NUP98-HOXA9 suggests that the disruption of hematopoietic differentiation by NUP98-HOXA9 is largely attributable to the NUP98 moiety and does not require DNA binding by the homeodomain. The fact that the isolated HOXA9 moiety (HOXA9ΔN) had no effect on differentiation shows that the NUP98 moiety is necessary for disruption of human hematopoietic differentiation. This is further confirmed by the observation that wild-type HOXA9 had only a mild effect on differentiation in spite of containing both the homeodomain and a transactivating domain.

**Table 2:** Summary of the effects of NUP98-HOXA9, NUP98-HOXA9/N51S, HOXA9, and HOXA9ΔN on the differentiation and proliferation of primary human CD34+ cells.

	NUP98-HOXA9	NUP98-HOXA9/N51S	HOXA9	HOXA9ΔN
**Erythroid differentiation**				
Morphologic maturity	↓↓↓	↓	NC	NC
CD235a	↓↓	↓↓	NC	NC
Erythroid cell No.	↑↑	↑↑	NC	NC
**Myeloid differentiation**				
Morphologic maturity	↓↓	↓	↓	NC
CD11b	↓↓	↓↓	NC	NC
Myeloid cell No.	↓↓	↓↓	NC	NC
**Proliferation**				
Liquid culture	↑↑↑	↑	↑	NC
LTC-IC	↑↑	↓	NC	NC
CFC cell number	↑↑	↑	NC	NC

NC indicates no change.

### The two moieties of NUP98-HOXA9 act synergistically to induce proliferation of primary human CD34+ cells

**Figure 10:**
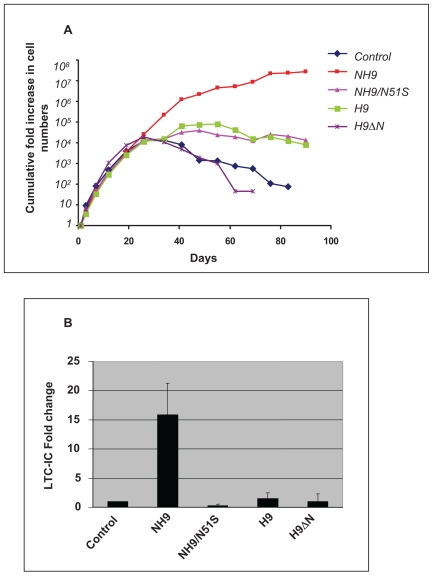
The two moieties of NUP98-HOXA9 synergistically increase proliferation of primary human CD34+ cells. Primary human CD34+ cells were retrovirally transduced with either control MSCV-IRES-GFP vector or vector expressing NUP98-HOXA9 (NH9), NUP98-HOXA9/N51S (NH9/N51S), HOXA9 (H9), or HOXA9ΔN (H9ΔN), and cells were sorted for GFP positivity. (A) The cumulative fold increase in cell numbers compared to day 0 is plotted on a logarithmic scale against time. The experiment was repeated 3 times; results from a representative experiment are shown. (B) LTC-IC assay showing the average fold change compared to control from 3 independent experiments. The error bars represent standard deviations.

NUP98-HOXA9 induces long-term proliferation of primary human CD34+ cells in liquid culture [15], [16]. In order to assess the relative contributions of the NUP98 and HOXA9 moieties to this proliferative effect, NUP98-HOXA9, NUP98-HOXA9/N51S, wild-type HOXA9, and HOXA9ΔN were introduced into primary human CD34+ hematopoietic cells by retroviral transduction, with empty retroviral vector as a control. Cells expressing the transduced constructs were selected by sorting for GFP and were cultured in liquid media with a cytokine cocktail as previously described [15] with periodic feeding and counting (Fig. 10A). The different samples showed similar growth profiles for approximately the first month, after which the numbers of control cells and cells expressing HOXA9ΔN began to decline. Cells expressing HOXA9 or NUP98-HOXA9/N51S showed a modest degree of growth for another few weeks before beginning to decline as well. As expected, NUP98-HOXA9 induced a marked increase in the number of cells in long-term liquid culture that was several orders of magnitude more than that of control cells.

Long-term proliferation of primary human CD34+ cells in liquid culture is usually associated with an increase in the numbers of primitive self-renewing cells. These cells are quantitated by the long-term culture-initiating cell (LTC-IC) assay [24]. As previously shown [15], NUP98-HOXA9 induced an increase in the numbers of LTC-ICs (Fig. 10B). On the other hand, NUP98-HOXA9/N51S, HOXA9, and HOXA9ΔN did not cause an increase in the numbers of LTC-ICs. Although HOXA9 is leukemogenic in mice [12] and immortalizes primary mouse hematopoietic cells *in vitro* [17], our data suggest that it does not have a similar effect on primary human hematopoietic cells.

The effects of NUP98-HOXA9, NUP98-HOXA9/N51S, HOXA9, and HOXA9ΔN on the proliferation of primary human CD34+ cells are summarized in Table 2. The modest increase in cell numbers induced by NUP98-HOXA9/N51S and HOXA9 in long-term culture compared to controls may reflect increased cell survival with a slight increase in proliferation. The data suggest that the effect of NUP98-HOXA9 on cell numbers is partially dependent on classical transactivation involving DNA binding and partially on mechanisms other than DNA binding. The two mechanisms appear to act synergistically since the effect of NUP98-HOXA9 on long-term proliferation and the number of LTC-ICs is more than the sum of the separate effects of HOXA9 and NUP98-HOXA9/N51S.

### Homeodomain-independent gene regulation by NUP98-HOXA9 in primary human CD34+ cells

**Figure 11:**
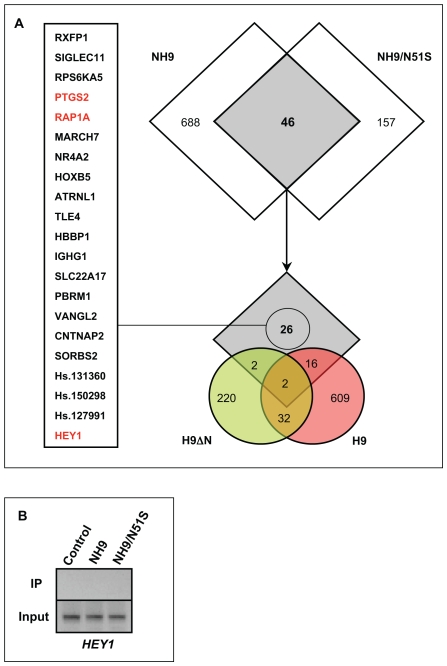
Homeodomain-independent gene regulation by NUP98-HOXA9 in primary human CD34+ cells. (A) Primary human CD34+ cells were retrovirally transduced with either control MSCV-IRES-GFP vector or vector expressing NUP98-HOXA9 (NH9), NUP98-HOXA9/N51S (NH9/N51S), HOXA9 (H9), or HOXA9ΔN (H9ΔN). Cells were sorted for GFP positivity and total RNA was subjected to microarray analysis. The experiment was performed two independent times and only genes that showed up- or down-regulation by 1.74 fold or more compared to control in both experiments were considered dysregulated. As shown in the upper Venn diagram, 46 probe sets showed dysregulation by both NUP98-HOXA9 (NH9) and NUP98-HOXA9/N51S (NH9/N51S). Of these, 26 did not show dysregulation by either HOXA9ΔN (H9ΔN) or HOXA9 (H9) and were considered homeodomain-independent (lower Venn diagram). These 26 probe sets represent 23 unique genes listed on the left. Genes with a potential role in homeodomain-independent transforming functions of NUP98-HOXA9 are highlighted in red and are discussed in the text. (B) Chromatin was immunoprecipitated with anti-Flag antibody from K562 cells nucleofected with either pTracer/CMV-Bsd vector (control), or vector containing Flag-tagged NUP98-HOXA9 (NH9) or NUP98-HOXA9/N51S (NH9/N51S). A segment of the *HEY1* promoter was amplified by PCR (see Materials and Methods for primer sequences). The upper lanes show immunoprecipitated chromatin (IP) and the lower lanes show input chromatin (Input).

The data described above show that disruption of the differentiation of primary human CD34+ cells by NUP98-HOXA9 is to a large extent independent of DNA binding by the homeodomain. To identify changes in gene expression that underlie this disrupted differentiation, expression microarray analysis was performed. Primary human CD34+ cells were transduced with retroviral vectors expressing NUP98-HOXA9, NUP98-HOXA9/N51S, wild-type HOXA9, and HOXA9ΔN as well as control empty vector, and were sorted for GFP expression. RNA was isolated from the cells and analyzed using the Affymetrix Human genome U133+2.0 array. The experiment was performed two independent times, and lists of probe sets showing a difference of 1.74-fold or more compared to control in both experiments were compiled (Tables S2, S3, S4, S5). As shown in Fig. 11A, 46 of the probe sets showed dysregulation by both NUP98-HOXA9 and NUP98-HOXA9/N51S. Some of these overlap probe sets showed dysregulation by HOXA9 and HOXA9ΔN (Fig. 11A) suggesting dysregulation by mechanisms involving homeodomain sequences (see Discussion). The remaining 26 probe sets represent 23 unique genes are dysregulated by mechanisms that require the NUP98 moiety but are apparently independent of the homeodomain (see Discussion). One of these genes, *HEY1*, is also dysregulated in K562 cells by retrovirally transduced NUP98-HOXA9 [6]. A ChIP assay was performed on the *HEY1* promoter [25], [26] in K562 cells expressing either NUP98-HOXA9 or its N51S mutant. As shown in Fig. 11B, neither NUP98-HOXA9 nor its N51S mutant bound to the *HEY1* promoter, supporting the notion that its transactivation is independent of direct homeodomain-DNA interaction. Among the genes listed in Fig. 11A, a few are notable for functions that may explain the transforming activities shared by NUP98-HOXA9 and NUP98-HOXA9/N51S:

#### RAP1A

This gene was downregulated by NUP98-HOXA9 and NUP98-HOXA9/N51S. It encodes a small GTPase of the Ras family that was identified as a suppressor of the transforming activity of oncogenic Ras in NIH/3T3 cells [27]. It was subsequently found to potentiate the functions of Ras in some cells and to antagonize them in others [28]. RAP1A functions in integrin-mediated adhesion and signaling and in the MAP kinase cascade [28]. It plays various roles in the proliferation and differentiation of hematopoietic cells [29]. For example, megakaryocytic, monocytic, and lymphoid differentiation of cell lines is associated with RAP1A induction [30], [31]. Maturation of megakaryocytes derived from human cord blood cells is also associated with induction of RAP1A [32]. Thrombopoietin-induced megakaryocytic differentiation is mediated by sustained activation of the ERK/MAPK pathway mediated by RAP1A activation and inhibition of megakaryocytic differentiation by stromal contact is associated with a block in RAP1A activation [31], [33]. Finally, RAP1A is activated by erythropoietin [34], raising the possibility that it plays a role in erythroid differentiation. Based on these data, the repression of RAP1A expression by both NUP98-HOXA9 and NUP98-HOXA9/N51S may play a role in their inhibition of both erythroid and myeloid differentiation.

#### PTGS2

This gene was upregulated by NUP98-HOXA9 and NUP98-HOXA9/N51S. It encodes prostaglandin-endoperoxide synthase 2 (prostaglandin G/H synthase 2), better known as cyclooxygenase-2 (COX-2). It is one of two isozymes, COX-1 and COX-2, that are responsible for the conversion of arachidonic acid to prostaglandins G_2_ and H_2_, which in turn give rise to a number of different prostaglandins [35]. COX-1 is constitutively expressed, whereas COX-2 is inducible and is overexpressed in many tumors resulting in overproduction of prostaglandins, including PGE2, which appears to play an important role in carcinogenesis [35]. Many types of cancer are known to overexpress COX-2 and there is a great deal of evidence that COX-2 inhibition with drugs such as aspirin can be used in the prevention and treatment of cancer [36]. A number of hematopoietic malignancies, including chronic myleogenous leukemia, chronic lymphocytic leukemia, lymphomas, and myeloma, have been shown to overexpress COX-2, which is associated with a worse prognosis [37]. There is evidence that the use of aspirin and other non-steroidal anti-inflammatory drugs (NSAIDs) is associated with a lower incidence of lymphomas and acute leukemia [38]. NSAID use has been found to reduce the risk of AML, particularly the FAB M2 subtype (AML with maturation) [39], which is the most common subtype in cases with NUP98 gene rearrangements [18]. Our data suggest that the induction of *PTGS2* expression by NUP98-HOXA9 is mediated by the NUP98 moiety and may contribute to the worse prognosis of AML patients with NUP98 gene rearrangements. It is possible that COX-2 blockade could help in the treatment of these leukemias.

#### HEY1

This gene (also known as *HERP2*) was upregulated by NUP98-HOXA9 and NUP98-HOXA9/N51S. It is a b-HLH protein that acts as a transcriptional repressor. It is an important mediator in the Notch signaling pathway [40] and has been implicated in the pathogenesis of several types of cancer including tumors of the brain, lung, pancreas, bone, skin, and kidney [41], [42], [43], [44], [45], [46]. Notch signaling is thought to play an important role in the self-renewal of hematopoietic stem cells [47] and plays an important role in the pathogenesis of hematologic malignancies, particularly T-lymphoblastic leukemia [48]. While the role of Notch signaling in AML is not well established, these data suggest that upregulation of HEY1 may contribute to the long-term proliferation and increased numbers of LTC-ICs induced by NUP98-HOXA9. There is evidence for involvement of HEY1 in erythroid differentiation: in a mouse cell line, Notch signaling associated with HEY1 upregulation resulted in increased the numbers of erythroid cells [49], whereas in primary human hematopoietic cells upregulation of HEY1 by JUN was associated with a block in erythroid differentiation [50]. The somewhat different results obtained in these two studies may reflect the different experimental systems used, but the data nevertheless suggest that the increased numbers of erythroid cells and the disrupted erythroid differentiation that we observed in primary human cells expressing NUP98-HOXA9 or NUP98-HOXA9/N51S may be mediated at least in part by upregulation of HEY1.

## Discussion

The luciferase reporter and ChIP data in K562 cells demonstrate at least two modes by which the NUP98-HOXA9 oncogene can dysregulate gene transcription: one that correlates with DNA-binding through the HOXA9 homeodomain and another that does not. The data from primary human CD34+ cells show that the latter mode is necessary and sufficient for most of the effects of NUP98-HOXA9 on differentiation.

The mechanisms by which NUP98-HOXA9/N51S modulates gene expression remain to be determined. An isolated NUP98 moiety (NUP98ΔC) that comprises the FG repeat region of NUP98 caused a mild disruption of myeloid differentiation, but did not have a significant effect on erythroid differentiation or proliferation (Fig. S1). This suggests that the FG repeat region may not be entirely sufficient for the disruption of differentiation. However, nucleoporin FG repeat regions are inherently disordered [51], [52] and it is likely that NUP98ΔC would misfold, mislocalize, and/or lose important protein-protein interactions making the results difficult to interpret. Based on gene expression analysis, a group of 46 probe sets were identified that show similar dysregulation by NUP98-HOXA9 and NUP98-HOXA9/N51S (Fig. 11A and Tables S2–S5) and therefore presumably do not require direct DNA binding by the homeodomain for their dysregulation. Some of these genes are also dysregulated by HOXA9 and/or HOXA9ΔN (Fig. 11A), suggesting that their dysregulation is mediated by non-DNA-binding parts of the homeodomain such as those that mediate dimerization with other proteins [53]. One possibility is that the homeodomain would interact with promoters indirectly by dimerizing with another DNA-binding factor. Such indirect binding may be difficult to detect with our ChIP assays and cannot be entirely excluded as a mechanism for the transactivation of the *PLN* promoter. Another possibility is that the homeodomain may disrupt transcription by titrating away transcription factors through dimerization. The remaining 26 probes represent 23 genes that appear to be homeodomain-independent (Fig. 11A) and their dysregulation requires the NUP98 moiety of NUP98-HOXA9. Based on the functions of the NUP98 moiety, an intriguing possibility is that expression of these genes may be dysregulated by a disruption in nucleocytoplasmic transport. The NUP98 portion of NUP98 fusions contains most or all of its FG repeats, which interact with nuclear transport carriers that mediate the nucleocytoplasmic transport of proteins [54], [55], [56]. It also contains the GLEBS motif that plays a role in RNA export by interacting with the mRNA export factor RAE1 [57]. Therefore homeodomain-independent functions of NUP98-HOXA9 (and other NUP98 fusions) may be mediated by disruption of the nucleocytoplasmic transport of transcription factors or RNAs that are important in myeloid differentiation and proliferation. Future experiments directed at identifying proteins that interact with NUP98-HOXA9 and examining its effects on nucleocytoplasmic transport may elucidate the homeodomain-independent mechanisms that contribute to the leukemogenic functions of NUP98 fusion proteins.

We also report here for the first time the effects of wild-type HOXA9 on primary human hematopoietic cells. HOXA9 is leukemogenic in mice, and it causes proliferation and blocks differentiation in primary mouse hematopoietic cells *in vitro* [12], [17]. While wild-type HOXA9 is overexpressed in a subset of human AML [58], [59], [60], [61], its effects on primary human hematopoietic cell differentiation and proliferation have not been previously reported, and its contribution to human myeloid leukemogenesis is not clear. In contrast to findings in mice, our data show that HOXA9 has modest effects on the proliferation and differentiation of primary human CD34+ cells. It causes a mild increase in the numbers of cells in long-term liquid culture and a mild block in myeloid maturation that is best appreciated on morphologic examination of Giemsa-stained cells (Tables 1 and 2). Microarray analysis of primary human CD34+ cells shows that HOXA9 dysregulates genes recognized by 659 probe sets compared to only 203 for NUP98-HOXA9/N51S (Fig. 11). It is therefore remarkable that NUP98-HOXA9/N51S causes a much more pronounced disruption of differentiation than HOXA9.

It is of interest to note that different differentiation markers did not give identical estimates of the extent of differentiation in our samples. For example, HOXA9 expression clearly decreased the numbers of mature myeloid cells and increased the numbers of less mature myeloid cells as judged by morphologic examination of Giemsa-stained Cytospin smears (Fig. 8 and Table 1). These findings are consistent with a block in myeloid maturation; yet flow cytometry did not reveal a significant difference in the expression of CD11b between HOXA9-expressing cells and controls (Fig. 9B). On the other hand, cells expressing NUP98-HOXA9/N51S did not show morphologic evidence of erythroid immaturity in Giemsa-stained preparations (Fig. 8 and Table 1); yet a clear block in erythroid differentiation was observed by flow cytometry as evidenced by decreased expression of CD235a (Fig. 9A). Thus, a combination of morphology and immunophenotyping can uncover subtle defects in hematopoietic differentiation that may not be otherwise obvious.

## Materials and Methods

### Plasmid construction

The pTracer-CMV/Bsd construct expressing HA-tagged NUP98-HOXA9 was previously described [15]. The N51S mutation was created in this construct by replacing a *Bgl*II/*Xba*I fragment with a synthetic fragment containing an A to G substitution in the 51^st^ codon of the homeodomain. The mutation was confirmed by sequencing. MSCV-IRES-GFP constructs expressing HA-tagged NUP98-HOXA9, and HOXA9 have been described [6]. NUP98-HOXA9/N51S and HOXA9ΔN [4] were subcloned upstream of IRES into MSCV-IRES-GFP. For luciferase constructs, the following promoter regions were amplified from human genomic DNA (Roche, Basel, Switzerland) by PCR using PfuUltra high-fidelity DNA polymerase (Stratagene, La Jolla, CA, USA): *HOXB6* from −1934 to +81; *JUN* from −2013 to +89; *KBTBD10* from −2369 to +76; *PLN* from −1937 to +81; and *SERPINE1* from −2012 to +74. PCR products were subcloned into pGL4.11 (Promega, Madison, WI, USA) upstream of the luciferase gene using the *Nhe*I/*Eco*RV sites for *HOXB6*, *JUN*, and *KBTBD10* promoters; the *Kpn*I/*Xho*I sites for the *PLN* promoter; and the *Xho*I/*Eco*RV sites for *SERPINE1* promoter. Deletions of the *KBTBD10* and *PLN* promoters are shown in Fig. 4A and 4B; they were similarly subcloned into pGL4.11. All sequences generated by PCR were confirmed by DNA sequencing.

### Retrovirus production

NUP98-HOXA9 retrovirus was previously described [15]. For the remaining retroviruses, GP293 cells were transiently transfected with 4.4 µg of retroviral vector and 1.1 µg of pVSV-G expression vector using Lipofectamine Plus reagent (Invitrogen, Carlsbad, CA, USA). After 48 h, the culture supernatant, containing VSV-pseudotyped retrovirus, was collected and used for transduction of PG13 packaging cells by spinoculation in the presence of 8 µg/mL polybrene (Hexadimethrine Bromide; Sigma-Aldrich Corp., St. Louis, MO, USA). The PG13 culture supernatant containing GaLV-pseudotyped retrovirus was used for transduction of CD34+ primary cells.

### Retroviral transduction and analysis of primary human CD34+ cells

Frozen human CD34+ cells purified from mobilized peripheral blood of two healthy volunteers were obtained from StemCell Technologies (Vancouver, British Columbia, Canada) and the Fred Hutchinson Cancer Research Center (Seattle, WA, USA); and of a deceased autologous transplant donor through the Bone Marrow Transplant Laboratory at Northwestern Memorial Hospital after Institutional Review Board approval. Cells were prestimulated and transduced with retrovirus as described [15]. After 46 h, GFP positive cells were isolated using a MoFlo high-speed sorter (Dako, Glostrup, Denmark) and expression of the transfected gene was confirmed by immunoblotting with anti-HA tag antibody. Long-term liquid culture of primary cells was performed in the presence of a cytokine cocktail as previously described [15]. Colony-forming cell (CFC) and long-term culture-initiating cell (LTC-IC) assays were performed as previously described [15]. Cytospin preparations of cells harvested from the CFC plates were stained with Giemsa and a 500-cell differential count was performed using an Olympus BX51 microscope (Olympus America, Center Valley, PA, USA). Cells with blast and promyelocyte morphology were counted as primitive; those with myelocyte/metamyelocyte morphology as intermediate myeloid; those with band, segmented neutrophil, monocyte, and macrophage morphology as mature myeloid; those with intermediate hemoglobinization as intermediate erythroid; and those with full hemoglobinization as mature erythroid. Photomicrographs were taken with an Olympus DP71 camera with a 60×oil objective.

### Flow cytometry

Flow cytometry was performed on a FACScan flow cytometer (BD Biosciences, San Jose, CA, USA) upgraded to 5 colors and two lasers, and analyzed using FCS3 Express (De Novo Software, Thornhill, Ontario, Canada) and FlowJO v8.6.3 (Tree Star, Inc., Ashland, OR) software. The antibodies used for these studies were CD11b (phycoerythrin-conjugated clone ICRF44) from eBioscience (San Diego, CA, USA); CD235a (allophycocyanin-conjugated clone GA-R2) from BD (Franklin Lakes, NJ, USA); and CD33 (allophycocyanin-conjugated clone D3HL60.251) and CD45 (phycoerythrin-Cy7-conjugated clone J.33) from Beckman Coulter (Miami, FL, USA).

### Microarray analysis of primary human CD34+ cells

Sorted GFP-positive cells were snap-frozen 3 days after transduction and submitted to the Siteman Cancer Center Laboratory for Clinical Genomics where total RNA was isolated and target preparation and microarray hybridization were performed. Labeled targets were hybridized to Affymetrix HG-U133 Plus 2.0 GeneChip microarrays. Data were merged with the updated gene annotation data for each probe set on the array using Spotfire DecisionSite 9.1.1 for Functional Genomics (Spotfire, Somerville, MA). Probe sets were filtered according to present/absent calls and the sets that were absent across all chips were filtered from the analysis. The fold change of each probe set was calculated by dividing the absolute signal intensity from NUP98-HOXA9, NUP98-HOXA9/N51S, HOXA9, and HOXA9ΔN samples by that of the control sample (vector only). Probes scored as increasing and absent in the numerator, and those scored as decreasing and absent in the denominator were also filtered out. The experiment was performed 2 independent times. Probe sets induced or repressed by 1.74-fold or greater in both experiments were considered differentially expressed. Gene intersection lists were generated using Spotfire DecisionSite 9.1.1 for Functional Genomics. All microarray data reported in the manuscript are described in accordance with MIAME guidelines.

### Luciferase assay

Cells were transfected by electroporation using a Bio-Rad GenePulser with 10 µg pGL4.11 vector or pGL4.11 driven by the indicated promoters and 20 µg of either empty pTracer-CMV/Bsd vector, or vector expressing HA-tagged NUP98-HOXA9 or NUP98-HOXA9/N51S. To control for efficiency of transfection, 0.5 µg of pRL-TK (Promega), which expresses Renilla luciferase, was included. Ten million cells were incubated with the DNA for 10 min at room temperature (RT) before electroporation and 10 min after electroporation, and were cultured in 20 mL IMDM media with 10% FBS, 2 mM L-glutamine, and 100 units/mL pencillin/streptomycin. Luciferase activity was measured 48 h after electroporation using the Dual Luciferase Reporter Assay System (Promega) and the results were normalized to Renilla luciferase.

### Chromatin Immunoprecipitation

K562 (10^7^) cells were nucleofected using a Nucleofector device (Lonza, Cologne, Germany) with either empty pTracer-CMV/Bsd vector or vector containing Flag-tagged NUP98-HOXA9 or NUP98-HOXA9/N51S and collected after 12 h. After crosslinking with 1% formaldehyde and quenching with 0.375 M glycine, cells were washed twice with ice-cold TBS. Nuclei were isolated by washing with 10 mM Tris-HCl, pH 7.5, 10 mM NaCl, 3 mM MgCl_2_, 0.5% NP-40 three times and were freeze-thawed. Nuclei were lysed by adding 200 µL of micrococcal nuclease buffer (10 mM Tris-HCl, pH 7.5, 200 mM NaCl, 3 mM MgCl_2_, 1 mM CaCl_2_, 4% NP-40, 3 mM EGTA, 1% SDS) and 1 mL FA lysis buffer (50 mM HEPES-NaOH, pH 7.5, 150 mM NaCl, 1 mM EDTA, 1% Triton X-100, 0.1% sodium deoxycholate, 0.1% SDS) with 2 mM PMSF and complete protease inhibitor cocktail (Roche). The lysate was sonicated and centrifuged at 17,000×g for 30 min at 4°C. The supernatant was precleared with Sepharose 4B beads (GE Healthcare, Piscataway, NJ, USA) for 2 h at 4°C and incubated with anti-Flag agarose M2 antibody (Sigma) overnight at 4°C. The beads were washed twice with FA lysis buffer, once with of FA lysis buffer with 500 mM NaCl, once with ChIP wash buffer (10 mM Tris-HCl, pH 7.5, 0.25 M LiCl, 1 mM EDTA, 0.5% NP-40, 0.5% sodium deoxycholate), and once with TE (10 mM Tris-HCl pH 7.5, 1 mM EDTA). The beads were eluted twice at 65°C for 10 min with a total of 250 µL of 1% SDS in TE. After adding 250 µl TE and 100 µg proteinase K, the eluate was incubated at 37°C for 2 h. Crosslinks were reversed at 65°C overnight followed by phenol chloroform extraction and DNA precipitation with 100 µL 4M LiCl and 1 mL ethanol. *KBTBD10*, *PLN*, and *HEY1* promoter regions were amplified by PCR (35 cycles) using the following primer pairs:

*KBTBD10:*

Forward: 5′CACATCTCATATTCTTGACCTTTGC3′

Reverse: 5′CTGGAACTTTTAATCACTAGGGAAAC3′

*PLN* (1):

Forward: 5′CCATAAAGTTGCCCTTAATACTGC3′

Reverse: 5′CTTGTGTGTATGGGAGAAGG3′

*PLN* (2):

Forward: 5′CCCGGCTTTACAAAAAAATTATTTCC3′

Reverse: 5′CACCCCCAAAATAGCAATATGC3′

*PLN* (3):

Forward:5′CAAATGATTTGCTCTAGAGATGGATTTTC3′

Reverse: 5′CGGGCATCATTCTCCTC3′

*HEY1*:

Forward: 5′TCACGTGGCACCCAGTACAC3′

Reverse: 5′GCATCTCATTTCCGGGGAAG3′

PCR products were resolved on 2% agarose gels and imaged with a Chemidoc XRS system (Bio-Rad).

## Supporting Information

Table S1 Early NUP98-HOXA9 target genes in K562 cells identified by microarray analysis. K562 cells were nucleofected with either control pTracer-CMV/Bsd plasmid or with plasmid expressing NUP98-HOXA9 followed by sorting for GFP expression. Cells were harvested 8 h after nucleofection and RNA was subjected to microarray analysis using Affymetrix HG-U133 Plus 2.0 GeneChip microarrays. The experiment was performed 2 independent times and genes showing 2-fold or higher dysregulation compared to control in both experiments are listed in the table. The fold change shown is the average of the 2 experiments. In cases where more than one probe corresponds to the same gene, the number shown represents the average fold change shown by all the probes representing that gene in the two experiments. Dysregulation of several genes was confirmed by quantitative RT-PCR. Reverse transcription was performed with the SuperScript III kit (Invitrogen), according to the manufacturer's protocol. Quantitative RT-PCR was performed using the GeneAmp 5700 sequence Detection System using iQ SYBR Green Supermix (Bio Rad). The signal intensities were normalized against glyceraldehyde-3-phosphate dehydrogenase (GAPDH); the numbers shown represent fold change compared to control.
(0.01 MB PDF)

Table S2 Genes dysregulated by NUP98-HOXA9. Primary human CD34+ cells were retrovirally transduced with either control MSCV-IRES-GFP vector or vector expressing NUP98-HOXA9. Cells were sorted for GFP positivity and total RNA was subjected to microarray analysis. The experiment was performed two independent times and only genes that showed up- or down-regulation by 1.74 fold or more compared to control in both experiments were considered dysregulated.
(0.07 MB PDF)

Table S3 Genes dysregulated by NUP98-HOXA9/N51S. Primary human CD34+ cells were retrovirally transduced with either control MSCV-IRES-GFP vector or vector expressing NUP98-HOXA9/N51S. Cells were sorted for GFP positivity and total RNA was subjected to microarray analysis. The experiment was performed two independent times and only genes that showed up- or down-regulation by 1.74 fold or more compared to control in both experiments were considered dysregulated.
(0.02 MB PDF)

Table S4 Genes dysregulated by wild-type HOXA9. Primary human CD34+ cells were retrovirally transduced with either control MSCV-IRES-GFP vector or vector expressing wild-type HOXA9. Cells were sorted for GFP positivity and total RNA was subjected to microarray analysis. The experiment was performed two independent times and only genes that showed up- or down-regulation by 1.74 fold or more compared to control in both experiments were considered dysregulated.
(0.06 MB PDF)

Table S5 Genes dysregulated by HOXA9ΔN. Primary human CD34+ cells were retrovirally transduced with either control MSCV-IRES-GFP vector or vector expressing HOXA9ΔN. Cells were sorted for GFP positivity and total RNA was subjected to microarray analysis. The experiment was performed two independent times and only genes that showed up- or down-regulation by 1.74 fold or more compared to control in both experiments were considered dysregulated.
(0.03 MB PDF)

Figure S1 The FG repeat region of NUP98-HOXA9 has a partial effect on differentiation. The NUP98 portion of NUP98-HOXA9 (NUP98ΔC) was subcloned into the MSCV-IRES-GFP vector. Primary human CD34+ cells were retrovirally transduced with either control MSCV-IRES-GFP vector or vector expressing NUP98ΔC, and were sorted for GFP positivity. (A) Flow cytometry for myeloid differentiation: Cells from CFC plates were harvested and stained with CD45, CD33 and CD11b; the CD33+ gate was plotted on a histogram to show CD11b expression compared to control (lower panel). The data show a mild decrease in the number of myeloid cells with shift to immaturity in cells expressing NUP98ΔC. (B) Flow cytometry for erythroid differentiation: Cells from CFC plates were harvested and stained with antibodies to CD45 and CD235a. The CD235a+ gate was plotted on a histogram (lower panel) to show the expression of CD235a relative to control cells. (C) Sorted cells were grown in liquid culture in the presence of cytokines and the cumulative fold increase in cell numbers compared to day 0 is plotted on a logarithmic scale against time.
(0.88 MB TIF)
