# Supplementary material for: Dissection of the Transformation of Primary Human Hematopoietic Cells by the Oncogene NUP98-HOXA9
Source: PLoS One. 2009 Aug 21;4(8):e6719. doi: 10.1371/journal.pone.0006719 (PMC2725295; doi:10.1371/journal.pone.0006719)
Supplement: Table S1 — Early NUP98-HOXA9 target genes in K562 cells identified by microarray analysis. K562 cells were nucleofected with either control pTracer-CMV/Bsd plasmid or with plasmid expressing NUP98-HOXA9 followed by sorting for GFP expression. Cells were harvested 8 h after nucleofection and RNA was subjected to microarray analysis using Affymetrix HG-U133 Plus 2.0 GeneChip microarrays. The experiment was performed 2 independent times and genes showing 2-fold or higher dysregulation compared to control in both experiments are listed in the table. The fold change shown is the average of the 2 experiments. In cases where more than one probe corresponds to the same gene, the number shown represents the average fold change shown by all the probes representing that gene in the two experiments. Dysregulation of several genes was confirmed by quantitative RT-PCR. Reverse transcription was performed with the SuperScript III kit (Invitrogen), according to the manufacturer's protocol. Quantitative RT-PCR was performed using the GeneAmp 5700 sequence Detection System using iQ SYBR Green Supermix (Bio Rad). The signal intensities were normalized against glyceraldehyde-3-phosphate dehydrogenase (GAPDH); the numbers shown represent fold change compared to control. (0.01 MB PDF) [file pone.0006719.s001.pdf]

Table S1. Early NUP98-HOXA9 target genes in K562 cells identified by microarray analysis

Gene Symbol	Fold Change	Accession Number	RT-PCR
KBTBD10	6.83	NM_006063	4.3
HOXB6	3.38	NM_018952.4	2.3
PLN	3.26	NM_002667.2	3.2
HPGD	2.94	J05594.1	2.9
HOXC6	2.83	NM_004503.3	2.7
DHRS9	2.55	AF240698.1	
CDKN2C	2.32	U17074.1	
EREG	2.32	NM_001432.2	
SGK	2.22	NM_005627.3	
FLJ21918	2.15	NM_024939.2	
ATP1B1	2.07	BC000006.2	
ZFPM2	2.00	NM_012082.2	
VMP1	-2.00	NM_030938.2	
PHLDA1	-2.00	NM_007350.3	
USP53	-2.00	NM_019050.2	
ATF3	-2.07	AB066566.1	
CDKN1A	-2.07	NM_000389.2	
DUSP1	-2.14	NM_004417.2	
KLF6	-2.15	AB017493.1	
COL1A1	-2.23	NM_000088.3	
AREG	-2.39	NM_001657	
JUN	-2.55	NM_002228.3	-2.4
SERPINE1	-2.93	NM_000602.1	-3.3
LOC387763	-3.88	XM_941665.2	
