# Supplementary material for: Dissection of the Transformation of Primary Human Hematopoietic Cells by the Oncogene NUP98-HOXA9
Source: PLoS One. 2009 Aug 21;4(8):e6719. doi: 10.1371/journal.pone.0006719 (PMC2725295; doi:10.1371/journal.pone.0006719)
Supplement: Table S2 — Genes dysregulated by NUP98-HOXA9. Primary human CD34+ cells were retrovirally transduced with either control MSCV-IRES-GFP vector or vector expressing NUP98-HOXA9. Cells were sorted for GFP positivity and total RNA was subjected to microarray analysis. The experiment was performed two independent times and only genes that showed up- or down-regulation by 1.74 fold or more compared to control in both experiments were considered dysregulated. (0.07 MB PDF) [file pone.0006719.s002.pdf]

Table S2. Genes dysregulated by NUP98-HOXA9

Probeset I.D.	Fold Change		Gene Name	Accession Number	Gene Symbol
	Exp.1	*Exp.2*			
227449_at	*212.00*	*9.03*	*EPH receptor A4*	*NM_004438*	*EPHA4*
204533_at	*140.19*	*21.16*	*chemokine (C-X-C motif) ligand 10*	*NM_001565*	*CXCL10*
206367_at	*124.48*	*191.00*	*renin*	*NM_000537*	*REN*
205552_s_at	*104.89*	*122.91*	*2',5'-oligoadenylate*	*NM_001032409*	*OAS1*
206987_x_at	*71.84*	*7.09*	*fibroblast growth factor 18*	*NM_003862*	*FGF18*
203153_at	*60.32*	*238.44*	*interferon-induced protein with tetratricopeptide repeats 1*	*NM_001001887*	*IFIT1*
204439_at	*56.10*	*150.47*	*interferon-induced protein 44-like*	*NM_006820*	*IFI44L*
202086_at	*52.00*	*135.85*	*myxovirus (influenza virus) resistance 1, interferon-inducible protein p78 (mouse)*	*NM_002462*	*MX1*
202411_at	*50.80*	*92.80*	*interferon, alpha-inducible protein 27*	*NM_005532*	*IFI27*
202869_at	*50.34*	*16.96*	*2',5'-oligoadenylate*	*NM_001032409*	*OAS1*
205984_at	*46.65*	*14.70*	*corticotropin releasing hormone binding protein*	*NM_001882*	*CRHBP*
212077_at	*42.42*	*7.22*	*caldesmon 1*	*NM_004342 /// NM_033138 /// NM_033139 /// NM_033140 /// NM_033157*	*CALD1*
228230_at	*31.51*	*3.14*		*NM_001037335 /// NM_033405*	
236892_s_at	*29.82*	*14.48*			
229057_at	*28.67*	*5.40*	*sodium channel, voltage-gated, type II, alpha subunit*	*NM_001040142 /// NM_001040143 /// NM_021007*	*SCN2A*
229441_at	*23.28*	*7.62*	*protease, serine, 23*	*NM_007173*	*PRSS23*
219932_at	*21.22*	*7.49*	*solute carrier family 27 (fatty acid transporter), member 6*	*NM_001017372 /// NM_014031*	*SLC27A6*
237261_at	*20.81*	*2.91*			
1554601_at	*19.96*	*1.97*	*Na+/K+ transporting*	*NM_001040214*	*NKAIN2*
207161_at	*18.84*	*7.35*	*KIAA0087*		*KIAA0087*
234563_at	*18.25*	*52.51*	*phosphodiesterase 3A, cGMP-inhibited*	*NM_000921*	*PDE3A*
210029_at	*17.58*	*7.44*	*indoleamine-pyrrole 2,3 dioxygenase*	*NM_002164*	*INDO*

220117_at	17.24	3.97	zinc finger protein 385D	NM_024697	ZNF385D
237496_at	17.12	7.66	3'-phosphoadenosine 5'-phosphosulfate synthase 2	NM_001015880 /// NM_004670	PAPSS2
213268_at	16.98	3.80	calmodulin binding	NM_015215	CAMTA1
239979_at	16.30	9.48	epithelial stromal interaction 1 (breast)	NM_001002264 /// NM_033255	EPSTI1
242002_at	15.42	8.17	Na+/K+ transporting	NM_001040214	NKAIN2
207952_at	15.15	156.82	interleukin 5 (colony-stimulating factor, eosinophil)	NM_000879	IL5
208425_s_at	14.28	3.27	tetratricopeptide repeat, ankyrin repeat and coiled-coil containing 2	XM_371074 /// XM_934927 /// XM_942334 /// XM_945909	TANC2
214059_at	14.26	9.51	interferon-induced protein 44	NM_006417	IFI44
226279_at	13.88	6.69	protease, serine, 23	NM_007173	PRSS23
204415_at	13.82	15.87	interferon, alpha-inducible protein 6	NM_002038 /// NM_022872 /// NM_022873	IFI6
229450_at	13.39	15.27			
226757_at	12.87	23.19	interferon-induced protein with tetratricopeptide repeats 2	NM_001547	IFIT2
205483_s_at	12.67	17.73	ISG15 ubiquitin-like modifier	NM_005101	ISG15
204747_at	12.61	18.14	interferon-induced protein with tetratricopeptide repeats 3	NM_001031683 /// NM_001549	IFIT3
227609_at	12.11	12.33	epithelial stromal interaction 1 (breast)	NM_001002264 /// NM_033255	EPSTI1
236407_at	11.73	2.82	potassium voltage-gated channel, Isk-related family, member 1	NM_000219	KCNE1
214453_s_at	11.45	11.09	interferon-induced protein 44	NM_006417	IFI44
1567878_at	11.38	12.00	defensin, beta 114	NM_001037499	DEFB114
205694_at	11.22	6.67	tyrosinase-related protein 1	NM_000550	TYRP1
235276_at	10.93	18.15			
221349_at	10.77	5.16	pre-B lymphocyte gene 1	NM_007128	VPREB1
242528_at	10.56	1.78		XM_929330 /// XM_939041	

208436_s_at	10.32	11.99	interferon regulatory factor 7	NM_001572 /// NM_004029 /// NM_004031	IRF7
1556209_at	10.17	3.92	C-type lectin domain family 2, member B	NM_005127	CLEC2B
242625_at	9.96	5.78	radical S-adenosyl methionine domain containing 2	NM_080657	RSAD2
204972_at	9.91	19.67	2'-5'-oligoadenylate	NM_001032731	OAS2
240561_at	9.66	2.01	muscleblind-like 3 (Drosophila)	NM_018388 /// NM_133486	MBNL3
228507_at	9.52	6.67			
205660_at	9.33	8.59	2'-5'-oligoadenylate synthetase-like	NM_003733 /// NM_198213	OASL
218400_at	9.11	10.49	2'-5'-oligoadenylate synthetase 3, 100kDa	NM_006187	OAS3
207836_s_at	9.03	6.66	RNA binding protein with multiple splicing	NM_001008710 /// NM_001008711 /// NM_001008712 /// NM_006867	RBPMS
228617_at	8.85	8.59	XIAP associated factor 1	NM_017523 /// NM_199139	XAF1
213797_at	8.37	7.69	radical S-adenosyl methionine domain containing 2	NM_080657	RSAD2
211597_s_at	8.19	7.84	HOP homeobox	NM_032495 /// NM_139211 /// NM_139212	HOPX
215571_at	8.09	30.60	neural cell adhesion molecule 2	NM_004540	NCAM2
241897_at	8.02	8.22	RNA binding protein with multiple splicing	NM_001008710 /// NM_001008711 /// NM_001008712 /// NM_006867	RBPMS
209487_at	7.73	6.83	RNA binding protein with multiple splicing	NM_001008710 /// NM_001008711 /// NM_001008712 /// NM_006867	RBPMS
219737_s_at	7.71	3.74	protocadherin 9	NM_020403 /// NM_203487	PCDH9
212097_at	7.64	2.40	caveolin 1, caveolae protein, 22kDa	NM_001753	CAV1
232979_at	7.56	3.22	homeobox B6	NM_018952	HOXB6

210797_s_at	*7.50*	*6.66*	*2'-5'-oligoadenylate synthetase-like*	*NM_003733 /// NM_198213*	*OASL*
205844_at	*7.23*	*6.68*	*vanin 1*	*NM_004666*	*VNN1*
209498_at	*7.14*	*2.08*	*carcinoembryonic antigen-related cell adhesion molecule 1 (biliary glycoprotein)*	*NM_001024912 /// NM_001712*	*CEACAM1*
220784_s_at	*7.11*	*56.99*	*urotensin 2*	*NM_006786 /// NM_021995*	*UTS2*
230047_at	*6.98*	*1.78*		*XM_370651 /// XM_925815 /// XM_937450 /// XM_943308*	
205572_at	*6.94*	*18.60*	*angiopoietin 2*	*NM_001147*	*ANGPT2*
238919_at	*6.93*	*3.47*	*protocadherin 9*	*NM_020403 /// NM_203487*	*PCDH9*
235521_at	*6.89*	*4.60*	*homeobox A3*	*NM_030661 /// NM_153631 /// NM_153632*	*HOXA3*
213183_s_at	*6.87*	*1.92*	*cyclin-dependent kinase inhibitor 1C (p57, Kip2)*	*NM_000076*	*CDKN1C*
243342_at	*6.81*	*6.15*	*inositol 1,4,5-*	*NM_002223*	*ITPR2*
201601_x_at	*6.78*	*7.97*	*interferon induced transmembrane protein 2 (1-8D)*	*NM_003641*	*IFITM2*
219677_at	*6.53*	*2.35*	*splA/ryanodine receptor domain and SOCS box containing 1*	*NM_025106*	*SPSB1*
237058_x_at	*6.47*	*6.46*	*solute carrier family 6 (neurotransmitter transporter, GABA), member 13*	*NM_016615*	*SLC6A13*
32128_at	*6.19*	*5.79*	*chemokine (C-C motif) ligand 18 (pulmonary and activation-regulated)*	*NM_002988*	*CCL18*
243271_at	*6.12*	*6.15*	*sterile alpha motif*	*NM_152703*	*SAMD9L*
202458_at	*6.09*	*4.43*	*protease, serine, 23*	*NM_007173*	*PRSS23*
207802_at	*5.98*	*51.90*	*cysteine-rich secretory protein 3*	*NM_006061*	*CRISP3*
215071_s_at	*5.95*	*6.67*	*histone cluster 1, H2ac*	*NM_003512*	*HIST1H2AC*
204994_at	*5.94*	*9.53*	*myxovirus (influenza virus) resistance 2 (mouse)*	*NM_002463*	*MX2*
242234_at	*5.94*	*6.74*	*XIAP associated factor 1*	*NM_017523 /// NM_199139*	*XAF1*
233072_at	*5.93*	*2.42*	*netrin G2*	*NM_032536*	*NTNG2*
237009_at	*5.90*	*4.51*	*CD69 molecule*	*NM_001781*	*CD69*
213369_at	*5.89*	*2.62*	*protocadherin 21*	*NM_033100*	*PCDH21*
1554524_a_at	*5.85*	*2.79*	*olfactomedin 3*	*NM_058170*	*OLFM3*

216020_at	5.80	3.76	interferon induced with helicase C domain 1	NM_022168	IFIH1
205513_at	5.76	5.11	transcobalamin I (vitamin B12 binding protein, R binder family)	NM_001062	TCN1
209795_at	5.74	4.45	CD69 molecule	NM_001781	CD69
233944_at	5.69	9.30	contactin associated protein-like 2	NM_014141	CNTNAP2
230036_at	5.66	5.98	sterile alpha motif	NM_152703	SAMD9L
219211_at	5.62	12.48	ubiquitin specific peptidase 18	NM_017414	USP18
209488_s_at	5.57	5.59	RNA binding protein with multiple splicing	NM_001008710 /// NM_001008711 /// NM_001008712 /// NM_006867	RBPMS
208557_at	5.56	2.46	homeobox A6	NM_024014	HOXA6
226145_s_at	5.53	8.92	Fraser syndrome 1	NM_025074	FRAS1
214341_at	5.43	3.15	adaptor-related protein complex 1, gamma 2 subunit	NM_003917 /// NM_080545	AP1G2
206382_s_at	5.22	9.88	brain-derived neurotrophic factor	NM_001709 /// NM_170731 /// NM_170732 /// NM_170733 /// NM_170734 /// NM_170735	BDNF
213348_at	5.22	3.61	cyclin-dependent kinase inhibitor 1C (p57, Kip2)	NM_000076	CDKN1C
228708_at	5.22	2.40	RAB27B, member RAS oncogene family	NM_004163	RAB27B
236300_at	5.22	7.49			
204684_at	5.21	1.90	neuronal pentraxin I	NM_002522	NPTX1
205749_at	5.18	6.53	cytochrome P450, family 1, subfamily A, polypeptide 1	NM_000499	CYP1A1
206133_at	5.11	5.50	XIAP associated factor 1	NM_017523 /// NM_199139	XAF1
230383_x_at	5.07	5.49			
226725_at	5.06	4.28			
232252_at	5.02	1.99	dual specificity phosphatase 27 (putative)	XM_043739 /// XM_935434 /// XM_935435 /// XM_942428 /// XM_945966 /// XM_945967	DUSP27
231804_at	5.02	1.74	relaxin/insulin-like family	NM_021634	RXFP1
213844_at	5.00	4.38	homeobox A5	NM_019102	HOXA5

206655_s_at	*4.98*	*1.89*	*glycoprotein Ib (platelet), beta polypeptide*	*NM_000407 /// NM_002688*	*GP1BB*
216215_s_at	*4.93*	*1.93*	*ribosomal protein L41*	*NM_001035267 /// NM_021104*	*RPL41*
226603_at	*4.90*	*5.58*	*sterile alpha motif*	*NM_152703*	*SAMD9L*
231382_at	*4.85*	*12.25*	*fibroblast growth factor 18*	*NM_003862*	*FGF18*
232757_at	*4.80*	*2.36*	*metastasis suppressor 1*	*NM_014751*	*MTSS1*
228531_at	*4.79*	*4.97*	*sterile alpha motif domain containing 9*	*NM_017654*	*SAMD9*
202350_s_at	*4.78*	*3.60*	*matrilin 2*	*NM_002380 /// NM_030583*	*MATN2*
1552715_a_at	*4.74*	*1.82*	*relaxin/insulin-like family*	*NM_021634*	*RXFP1*
218943_s_at	*4.74*	*4.26*	*DEAD (Asp-Glu-Ala-Asp) box polypeptide 58*	*NM_014314*	*DDX58*
205600_x_at	*4.68*	*3.52*	*homeobox B5*	*NM_002147*	*HOXB5*
219106_s_at	*4.63*	*3.26*	*kelch repeat and BTB (POZ) domain containing 10*	*NM_006063*	*KBTBD10*
242416_at	*4.55*	*2.19*	*protein tyrosine phosphatase, receptor type, G*	*NM_002841*	*PTPRG*
203596_s_at	*4.53*	*20.02*	*interferon-induced protein with tetratricopeptide repeats 5*	*NM_012420*	*IFIT5*
244503_at	*4.51*	*9.32*	*brain-derived neurotrophic factor*	*NM_001709 /// NM_170731 /// NM_170732 /// NM_170733 /// NM_170734 /// NM_170735*	*BDNF*
210834_s_at	*4.50*	*2.23*	*prostaglandin E receptor 3 (subtype EP3)*	*NM_000957 /// NM_198712 /// NM_198713 /// NM_198714 /// NM_198715 /// NM_198716 /// NM_198717 /// NM_198718 /// NM_198719*	*PTGER3*
223565_at	*4.40*	*2.18*		*NM_016459*	
AFFX-HUMRGE/M10098_M_at	*4.36*	*1.84*			
214022_s_at	*4.32*	*5.56*	*interferon induced transmembrane protein 2 (1-8D)*	*NM_003641*	*IFITM2*
206446_s_at	*4.29*	*3.10*		*NM_033440*	

233888_s_at	4.28	3.74	SLIT-ROBO Rho GTPase activating protein 1	NM_020762	SRGAP1
203789_s_at	4.22	3.50	sema domain, immunoglobulin domain (Ig), short basic domain, secreted, (semaphorin) 3C	NM_006379	SEMA3C
1554314_at	4.18	2.43	chromosome 6 open reading frame 141	NM_153344	C6orf141
242907_at	4.17	6.21	guanylate binding protein 2, interferon-inducible	NM_004120	GBP2
232088_x_at	4.15	1.77			
206553_at	4.14	7.67	2'-5'-oligoadenylate	NM_001032731	OAS2
204748_at	4.08	3.47	prostaglandin-endoperoxide synthase 2 (prostaglandin G/H synthase and cyclooxygenase)	NM_000963	PTGS2
218986_s_at	4.08	6.10	DEAD (Asp-Glu-Ala-Asp) box polypeptide 60	NM_017631	DDX60
212942_s_at	4.07	2.97	KIAA1199	NM_018689	KIAA1199
212224_at	4.05	4.22	aldehyde dehydrogenase 1 family, member A1	NM_000689	ALDH1A1
1552908_at	4.04	2.01	chromosome 1 open reading frame 150	NM_145278	C1orf150
218330_s_at	3.96	2.18	neuron navigator 2	NM_145117 /// NM_182964	NAV2
219352_at	3.96	3.42	hect domain and RLD 6	NM_017912	HERC6
201508_at	3.93	1.84	insulin-like growth factor binding protein 4	NM_001552	IGFBP4
202145_at	3.87	3.64	lymphocyte antigen 6 complex, locus E	NM_002346	LY6E
213362_at	3.85	2.03	protein tyrosine phosphatase, receptor type, D	NM_001040712 /// NM_002839 /// NM_130391 /// NM_130392 /// NM_130393	PTPRD
244764_at	3.84	5.14	human immunodeficiency virus type I enhancer binding protein 3	NM_024503	HIVEP3
210666_at	3.79	1.94	iduronate 2-sulfatase (Hunter syndrome)	NM_000202 /// NM_006123	IDS
230000_at	3.79	4.10	ring finger protein 213	NM_020914	RNF213
1553204_at	3.77	2.35	chromosome 20 open reading frame 200	NM_152757	C20orf200

244625_at	3.77	1.93	arginine-glutamic acid dipeptide (RE) repeats	NM_001042681 /// NM_001042682 /// NM_012102	RERE
238649_at	3.75	3.03	phosphatidylinositol transfer protein, cytoplasmic 1	NM_012417 /// NM_181671	PITPNC1
219478_at	3.74	4.73	WAP four-disulfide core domain 1	NM_021197	WFDC1
1561292_at	3.72	2.24			
AFFX-HUMISGF3A/M97935_5_at	3.71	4.90	signal transducer and activator of transcription 1, 91kDa	NM_007315 /// NM_139266	STAT1
238575_at	3.71	1.98	oxysterol binding protein-like 6	NM_032523 /// NM_145739	OSBPL6
219684_at	3.68	5.40	receptor (chemosensory) transporter protein 4	NM_022147	RTP4
236949_at	3.67	9.32	RAR-related orphan receptor A	NM_002943 /// NM_134260 /// NM_134261 /// NM_134262	RORA
243923_at	3.63	2.14			
228607_at	3.62	8.52	2'-5'-oligoadenylate synthetase 2, 69/71kDa	NM_001032731 /// NM_002535 /// NM_016817	OAS2
200887_s_at	3.60	4.07	signal transducer and activator of transcription 1, 91kDa	NM_007315 /// NM_139266	STAT1
214596_at	3.60	5.95			
229199_at	3.58	3.18			
1559037_a_at	3.57	5.51	HEG homolog 1	XM_087386	HEG1
201693_s_at	3.57	1.76	early growth response 1	NM_001964	EGR1
222793_at	3.57	3.24	DEAD (Asp-Glu-Ala-Asp) box polypeptide 58	NM_014314	DDX58
206493_at	3.57	2.19	integrin, alpha 2b (platelet glycoprotein IIb of IIb/IIIa complex, antigen CD41)	NM_000419	ITGA2B
44783_s_at	3.56	7.08	hairy/enhancer-of-split related with YRPW motif 1	NM_001040708 /// NM_012258	HEY1
209969_s_at	3.56	4.87	signal transducer and activator of transcription 1, 91kDa	NM_007315 /// NM_139266	STAT1
223220_s_at	3.54	4.85	poly (ADP-ribose) polymerase family, member 9	NM_031458	PARP9
242488_at	3.52	3.41			

227809_at	*3.52*	*2.26*	*zinc finger CCCH-type containing 6*	*NM_198581*	*ZC3H6*
211889_x_at	*3.50*	*2.57*	*carcinoembryonic antigen-related cell adhesion molecule 1 (biliary glycoprotein)*	*NM_001024912 /// NM_001712*	*CEACAM1*
212062_at	*3.48*	*2.45*	*ATPase, class II, type 9A*	*NM_006045*	*ATP9A*
235643_at	*3.48*	*4.31*	*sterile alpha motif*	*NM_152703*	*SAMD9L*
210831_s_at	*3.48*	*8.86*	*prostaglandin E receptor 3 (subtype EP3)*	*NM_000957 /// NM_198712 /// NM_198713 /// NM_198714 /// NM_198715 /// NM_198716 /// NM_198717 /// NM_198718 /// NM_198719*	*PTGER3*
228904_at	*3.45*	*3.07*	*homeobox B3*	*NM_002146*	*HOXB3*
220298_s_at	*3.43*	*2.13*	*spermatogenesis associated 6*	*NM_019073*	*SPATA6*
213058_at	*3.41*	*2.05*	*tetratricopeptide repeat domain 28*	*XM_929318 /// XM_939083*	*TTC28*
244605_at	*3.41*	*3.19*			
239555_at	*3.39*	*2.01*	*v-yes-1 Yamaguchi sarcoma viral related oncogene homolog*	*NM_002350*	*LYN*
202526_at	*3.39*	*2.97*	*SMAD family member 4*	*NM_005359*	*SMAD4*
215146_s_at	*3.39*	*2.73*	*tetratricopeptide repeat domain 28*	*XM_929318 /// XM_939083*	*TTC28*
204981_at	*3.37*	*2.57*	*solute carrier family 22, member 18*	*NM_002555 /// NM_183233*	*SLC22A18*
228152_s_at	*3.36*	*5.69*	*DEAD (Asp-Glu-Ala-Asp) box polypeptide 60-like*	*XM_037817 /// XM_930964 /// XM_930969 /// XM_930988 /// XM_930996 /// XM_931005 /// XM_940236 /// XM_944501 /// XM_944502 /// XM_944508 /// XM_944511 /// XM_944514*	*DDX60L*
219534_x_at	*3.36*	*2.67*	*cyclin-dependent kinase inhibitor 1C (p57, Kip2)*	*NM_000076*	*CDKN1C*
203882_at	*3.32*	*4.86*	*interferon regulatory factor 9*	*NM_006084*	*IRF9*
206289_at	*3.31*	*2.05*	*homeobox A4*	*NM_002141*	*HOXA4*
243846_x_at	*3.31*	*5.59*		*XM_926804*	

336_at	3.31	1.96	thromboxane A2 receptor	NM_001060 /// NM_201636	TBXA2R
231365_at	3.28	2.19			
204187_at	3.27	2.41	guanosine monophosphate reductase	NM_006877	GMPR
1552783_at	3.25	3.31	zinc finger protein 417	NM_152475	ZNF417
1559315_s_at	3.25	2.11		XM_931697 ///	
226065_at	3.25	2.58	prickle homolog 1 (Drosophila)	NM_153026	PRICKLE1
204918_s_at	3.24	2.02	myeloid/lymphoid or mixed-lineage leukemia (trithorax homolog, Drosophila); translocated to, 3	NM_004529	MLLT3
226189_at	3.24	8.17	integrin, beta 8	NM_002214	ITGB8
235885_at	3.23	64.82	purinergic receptor P2Y, G-protein coupled, 12	NM_022788 /// NM_176876	P2RY12
209732_at	3.22	3.35	C-type lectin domain family 2, member B	NM_005127	CLEC2B
206847_s_at	3.21	1.86	homeobox A7	NM_006896	HOXA7
202241_at	3.19	2.05	tribbles homolog 1 (Drosophila)	NM_025195	TRIB1
205352_at	3.18	2.31	serpin peptidase inhibitor, clade I (neuroserpin), member 1	NM_005025	SERPINI1
37802_r_at	3.18	2.39	family with sequence similarity 63, member B	NM_001040450 /// NM_001040453	FAM63B
228886_at	3.18	1.75	leucine rich repeat containing 27	NM_030626	LRRC27
223805_at	3.18	2.72	oxysterol binding protein-like 6	NM_032523 /// NM_145739	OSBPL6
235157_at	3.17	6.30	poly (ADP-ribose) polymerase family, member 14	NM_017554	PARP14
229309_at	3.14	3.31			
238430_x_at	3.14	2.67	schlafen family member 5	NM_144975	SLFN5
239698_at	3.13	2.74			
204044_at	3.11	2.39	quinolinate phosphoribosyltransferase (nicotinate-nucleotide pyrophosphorylase (carboxylating))	NM_014298	QPRT
212274_at	3.06	2.10	lipin 1	NM_145693	LPIN1
206447_at	3.06	1.76		NM_033440	
209417_s_at	3.05	4.69	interferon-induced protein 35	NM_005533	IFI35

227195_at	*3.05*	*6.86*	*zinc finger protein 503*	*NM_032772*	*ZNF503*
1554319_at	*3.05*	*6.18*	*ribosomal protein S6 kinase, 90kDa, polypeptide 5*	*NM_004755 /// NM_182398*	*RPS6KA5*
231956_at	*3.03*	*2.55*	*KIAA1618*	*NM_020954*	*KIAA1618*
219209_at	*3.02*	*2.94*	*interferon induced with helicase C domain 1*	*NM_022168*	*IFIH1*
213182_x_at	*3.01*	*2.28*	*cyclin-dependent kinase inhibitor 1C (p57, Kip2)*	*NM_000076*	*CDKN1C*
223980_s_at	*2.99*	*3.11*	*SP110 nuclear body protein*	*NM_004509 /// NM_004510 /// NM_080424*	*SP110*
240539_at	*2.99*	*2.46*	*autism susceptibility candidate 2*	*NM_015570*	*AUTS2*
204205_at	*2.98*	*3.07*	*apolipoprotein B mRNA editing enzyme, catalytic polypeptide-like 3G*	*NM_021822*	*APOBEC3G*
203485_at	*2.97*	*2.89*	*reticulon 1*	*NM_021136 /// NM_206852 /// NM_206857*	*RTN1*
237483_at	*2.97*	*3.12*	*pleckstrin homology domain containing, family A member 5*	*NM_019012*	*PLEKHA5*
209374_s_at	*2.95*	*3.58*	*immunoglobulin heavy constant mu*		*IGHM*
231577_s_at	*2.95*	*4.29*	*guanylate binding protein 1, interferon-inducible, 67kDa*	*NM_002053*	*GBP1*
244579_at	*2.95*	*2.13*	*trichorhinophalangeal syndrome I*	*NM_014112*	*TRPS1*
219863_at	*2.95*	*3.21*	*hect domain and RLD 5*	*NM_016323*	*HERC5*
209905_at	*2.92*	*3.58*	*homeobox A9*	*NM_152739*	*HOXA9*
212820_at	*2.92*	*2.80*	*Dmx-like 2*	*NM_015263*	*DMXL2*
230008_at	*2.91*	*2.16*	*thrombospondin, type I, domain containing 7A*	*XM_371877 /// XM_374404 /// XM_928187 /// XM_932697 /// XM_935904 /// XM_935905 /// XM_937164 /// XM_943202 /// XM_943205*	*THSD7A*
202269_x_at	*2.91*	*3.30*	*guanylate binding protein 1, interferon-inducible, 67kDa*	*NM_002053*	*GBP1*
242961_x_at	*2.90*	*3.50*	*DEAD (Asp-Glu-Ala-Asp) box polypeptide 58*	*NM_014314*	*DDX58*
203373_at	*2.89*	*1.81*	*suppressor of cytokine signaling 2*	*NM_003877*	*SOCS2*

209870_s_at	2.89	2.25	amyloid beta (A4) precursor protein-binding, family A, member 2 (X11-like)	NM_005503	APBA2
1570623_at	2.89	5.43			
243473_at	2.89	1.93		NM_025164	
215446_s_at	2.89	6.33	lysyl oxidase	NM_002317	LOX
1562863_at	2.88	2.18	exostoses (multiple) 1	NM_000127	EXT1
244424_at	2.88	2.00			
227703_s_at	2.87	2.41	synaptotagmin-like 4 (granuphilin-a)	NM_080737	SYTL4
1554568_at	2.87	2.52	dynein, light chain, roadblock-type 1	NM_014183	DYNLRB1
209640_at	2.87	1.96	promyelocytic leukemia	NM_002675 /// NM_033238 /// NM_033239 /// NM_033240 /// NM_033244 /// NM_033246 /// NM_033247 /// NM_033249 /// NM_033250 /// XM_941774	PML
1552480_s_at	2.87	2.05	protein tyrosine phosphatase, receptor type, C	NM_002838 /// NM_080921 /// NM_080922 /// NM_080923	PTPRC
218675_at	2.86	2.52	solute carrier family 22, member 17	NM_016609 /// NM_020372	SLC22A17
1562235_s_at	2.85	3.18	pre-B-cell leukemia	NM_002585	PBX1
227273_at	2.84	3.27			
241347_at	2.81	2.94	KIAA1618	NM_020954	KIAA1618
1558871_at	2.80	2.15			
212698_s_at	2.80	2.39	septin 10	NM_144710 /// NM_178584	10-Sep
207018_s_at	2.80	2.05	RAB27B, member RAS oncogene family	NM_001001713 /// NM_004163 /// NM_007341	RAB27B
1558990_at	2.79	2.22	chromosome 18 open reading frame 21	NM_031446	C18orf21
1569652_at	2.79	2.61	myeloid/lymphoid or mixed-lineage leukemia (trithorax homolog, Drosophila); translocated to, 3	NM_004529	MLLT3
1561181_at	2.78	2.52	AT rich interactive domain 5B (MRF1-like)	NM_032199	ARID5B
216956_s_at	2.78	1.90	integrin, alpha 2b (platelet glycoprotein IIb of IIb/IIIa complex, antigen CD41)	NM_000419	ITGA2B

218543_s_at	2.77	2.83	poly (ADP-ribose) polymerase family, member 12	NM_022750	PARP12
218999_at	2.77	3.12	transmembrane protein 140	NM_018295	TMEM140
232610_at	2.77	2.75	poly (ADP-ribose) polymerase family, member 14	NM_017554	PARP14
205856_at	2.72	48.06	solute carrier family 14 (urea transporter), member 1 (Kidd blood group)	NM_015865	SLC14A1
203595_s_at	2.71	6.04	interferon-induced protein with tetratricopeptide repeats 5	NM_012420	IFIT5
206831_s_at	2.71	4.80	arylsulfatase D	NM_001669 /// NM_009589	ARSD
34408_at	2.70	2.35	reticulon 2	NM_005619 /// NM_206900 /// NM_206901 /// NM_206902	RTN2
204141_at	2.70	2.11	tubulin, beta 2A	NM_001069	TUBB2A
1565821_at	2.69	2.32			
242172_at	2.69	1.81	Meis homeobox 1	NM_002398	MEIS1
206310_at	2.68	2.75	serine peptidase inhibitor, Kazal type 2 (acrosin-trypsin inhibitor)	NM_021114	SPINK2
237409_at	2.67	2.67	death-associated protein kinase 1	NM_004938	DAPK1
212203_x_at	2.67	2.69	interferon induced transmembrane protein 3 (1-8U)	NM_021034	IFITM3
239336_at	2.66	2.04	thrombospondin 1	NM_003246	THBS1
235046_at	2.66	1.90			
236034_at	2.66	17.67			
213578_at	2.65	2.37	bone morphogenetic protein receptor, type IA	NM_004329	BMPR1A
241978_at	2.65	1.88	aldo-keto reductase family 1, member A1 (aldehyde reductase)	NM_006066 /// NM_153326	AKR1A1
235185_s_at	2.64	2.67	sequestosome 1	NM_003900	SQSTM1
223179_at	2.64	2.76	yippee-like 3 (Drosophila)	NM_031477	YPEL3
240439_at	2.63	4.08			
226702_at	2.62	2.59	cytidine monophosphate (UMP-CMP) kinase 2, mitochondrial	NM_207315	CMPK2

203304_at	*2.62*	*2.35*	*BMP and activin membrane-bound inhibitor homolog (Xenopus laevis)*	*NM_012342*	*BAMBI*
1562591_a_at	*2.62*	*2.23*	*orofacial cleft 1*	*NM_153003*	*OFCC1*
224559_at	*2.61*	*2.10*	*metastasis associated lung adenocarcinoma transcript 1 (non-protein coding)*	*NR_002819*	*MALAT1*
241501_at	*2.61*	*2.19*	*Rho GTPase activating protein 25*	*NM_001007231 /// NM_014882*	*ARHGAP25*
219235_s_at	*2.61*	*1.81*	*phosphatase and actin regulator 4*	*NM_023923*	*PHACTR4*
1554205_s_at	*2.60*	*2.67*	*islet cell autoantigen*	*NM_138468 ///*	*ICA1L*
225929_s_at	*2.60*	*4.10*	*ring finger protein 213*	*NM_020914*	*RNF213*
1556568_a_at	*2.60*	*1.98*	*nemo-like kinase*	*NM_016231*	*NLK*
210461_s_at	*2.60*	*2.06*	*actin binding LIM protein 1*	*NM_001003407 /// NM_001003408 /// NM_002313 /// NM_006720*	*ABLIM1*
225239_at	*2.59*	*1.81*			
209859_at	*2.58*	*4.84*	*tripartite motif-containing 9*	*NM_015163 /// NM_052978*	*TRIM9*
206381_at	*2.58*	*2.30*	*sodium channel, voltage-gated, type II, alpha subunit*	*NM_001040142 /// NM_001040143 /// NM_021007*	*SCN2A*
1569238_a_at	*2.57*	*2.05*	*Rap guanine nucleotide exchange factor (GEF) 2*	*XM_376350 /// XM_930387 /// XM_934710 /// XM_934712 /// XM_934714 /// XM_934715 /// XM_934716 /// XM_934717 /// XM_934718 /// XM_939972 /// XM_944390 /// XM_944396 /// XM_944398 /// XM_944400 /// XM_944402 /// XM_944403 /// XM_944410 /// XM_944412*	*RAPGEF2*
206478_at	*2.57*	*1.94*	*KIAA0125*	*NM_014792*	*KIAA0125*
227034_at	*2.55*	*2.48*	*ankyrin repeat domain 57*	*NM_023016*	*ANKRD57*

237727_at	*2.55*	*2.34*	*FRAS1 related extracellular matrix 1*	*NM_144966*	*FREM1*
1553705_a_at	*2.55*	*2.78*			
235175_at	*2.55*	*3.47*	*guanylate binding protein 4*	*NM_052941*	*GBP4*
228935_at	*2.54*	*2.00*	*solute carrier family 4, sodium bicarbonate cotransporter, member 8*	*NM_001039960 /// NM_004858*	*SLC4A8*
227062_at	*2.53*	*2.00*		*NR_002802*	
1553729_s_at	*2.53*	*2.15*	*leucine rich repeat*	*NM_152759*	*LRRC43*
210129_s_at	*2.51*	*2.02*	*tubulin tyrosine ligase-like family, member 3*	*NM_001025930 /// NM_015644*	*TTLL3*
225636_at	*2.51*	*2.86*	*signal transducer and activator of transcription 2, 113kDa*	*NM_005419*	*STAT2*
229461_x_at	*2.51*	*2.97*	*neuronal growth regulator 1*	*NM_173808*	*NEGR1*
203662_s_at	*2.50*	*3.65*	*tropomodulin 1*	*NM_003275*	*TMOD1*
202748_at	*2.50*	*3.45*	*guanylate binding protein 2, interferon-inducible*	*NM_004120*	*GBP2*
225540_at	*2.50*	*8.28*	*microtubule-associated protein 2*	*NM_001039538 /// NM_002374 /// NM_031845 /// NM_031847*	*MAP2*
243999_at	*2.49*	*22.29*	*schlafen family member 5*	*NM_144975*	*SLFN5*
212827_at	*2.49*	*4.32*	*immunoglobulin heavy constant mu*		*IGHM*
201236_s_at	*2.49*	*2.90*	*BTG family, member 2*	*NM_006763*	*BTG2*
235478_at	*2.48*	*1.91*	*DNA cross-link repair 1C (PSO2 homolog, S. cerevisiae)*	*NM_001033855 /// NM_001033857 /// NM_001033858 /// NM_022487*	*DCLRE1C*
204069_at	*2.48*	*1.85*	*Meis homeobox 1*	*NM_002398*	*MEIS1*
AFFX-HUMISGF3A/ M97935_3_at	*2.46*	*3.61*	*signal transducer and activator of transcription 1, 91kDa*	*NM_007315 /// NM_139266*	*STAT1*
240867_at	*2.46*	*2.14*	*regulatory factor X, 3 (influences HLA class II expression)*	*NM_002919 /// NM_134428*	*RFX3*
219691_at	*2.45*	*4.25*	*sterile alpha motif domain containing 9*	*NM_017654*	*SAMD9*
201242_s_at	*2.45*	*2.40*	*ATPase, Na+/K+ transporting, beta 1 polypeptide*	*NM_001001787 /// NM_001677*	*ATP1B1*

224403_at	*2.45*	*13.91*	*Fc receptor-like 4*	*NM_031282*	*FCRL4*
213294_at	*2.45*	*2.73*			
207095_at	*2.44*	*3.41*	*solute carrier family 10 (sodium/bile acid cotransporter family), member 2*	*NM_000452*	*SLC10A2*
201641_at	*2.44*	*3.35*	*bone marrow stromal cell antigen 2*	*NM_004335*	*BST2*
208012_x_at	*2.44*	*3.17*	*SP110 nuclear body protein*	*NM_004509 /// NM_004510 /// NM_080424*	*SP110*
218821_at	*2.43*	*2.50*	*aminopeptidase-like 1*	*NM_024663*	*NPEPL1*
204082_at	*2.42*	*3.20*	*pre-B-cell leukemia homeobox 3*	*NM_006195*	*PBX3*
209459_s_at	*2.42*	*2.21*	*4-aminobutyrate aminotransferase*	*NM_000663 /// NM_020686*	*ABAT*
227839_at	*2.41*	*1.92*	*methyl-CpG binding domain protein 5*	*NM_018328*	*MBD5*
1562876_s_at	*2.41*	*1.93*		*XR_001013*	
234033_at	*2.41*	*2.12*	*Rap guanine nucleotide exchange factor (GEF) 2*	*XM_376350 /// XM_930387 /// XM_934710 /// XM_934712 /// XM_934714 /// XM_934715 /// XM_934716 /// XM_934717 /// XM_934718 /// XM_939972 /// XM_944390 /// XM_944396 /// XM_944398 /// XM_944400 /// XM_944402 /// XM_944403 /// XM_944410 /// XM_944412*	*RAPGEF2*
232517_s_at	*2.40*	*2.25*		*NM_001037335 /// NM_033405*	
1557403_s_at	*2.40*	*1.75*	*Rho guanine nucleotide exchange factor (GEF) 12*	*NM_015313*	*ARHGEF12*
1564473_at	*2.40*	*2.03*	*establishment of cohesion 1 homolog 2 (S. cerevisiae)*	*NM_001017420*	*ESCO2*
AFFX-HUMISGF3A/M97935_MA_at	*2.40*	*3.53*	*signal transducer and activator of transcription 1, 91kDa*	*NM_007315 /// NM_139266*	*STAT1*
1559413_at	*2.38*	*3.82*	*t-complex 11 (mouse)-like 2*	*NM_152772*	*TCP11L2*

227807_at	*2.38*	*2.63*	*poly (ADP-ribose) polymerase family, member 9*	*NM_031458*	*PARP9*
225415_at	*2.38*	*2.69*	*deltex 3-like (Drosophila)*	*NM_138287*	*DTX3L*
238210_at	*2.38*	*1.80*	*RYK receptor-like tyrosine kinase*	*NM_001005861 /// NM_002958*	*RYK*
238176_at	*2.38*	*2.16*	*Rap guanine nucleotide exchange factor (GEF) 2*	*XM_376350 /// XM_930387 /// XM_934710 /// XM_934712 /// XM_934714 /// XM_934715 /// XM_934716 /// XM_934717 /// XM_934718 /// XM_939972 /// XM_944390 /// XM_944396 /// XM_944398 /// XM_944400 /// XM_944402 /// XM_944403 /// XM_944410 /// XM_944412*	*RAPGEF2*
204529_s_at	*2.36*	*4.03*	*thymocyte selection-associated high mobility group box*	*NM_014729*	*TOX*
1555474_at	*2.36*	*1.80*	*tubulin tyrosine ligase-like family, member 3*	*NM_001025930 /// NM_015644*	*TTLL3*
242079_at	*2.35*	*1.86*	*regulator of G-protein signaling 12*	*NM_002926 /// NM_198227 /// NM_198229*	*RGS12*
213293_s_at	*2.34*	*2.60*	*tripartite motif-containing 22*	*NM_006074*	*TRIM22*
241508_at	*2.34*	*1.80*	*ankyrin repeat domain 12*	*NM_015208*	*ANKRD12*
232375_at	*2.34*	*3.53*	*signal transducer and activator of transcription 1, 91kDa*	*NM_007315 /// NM_139266*	*STAT1*
232213_at	*2.34*	*2.15*	*pellino homolog 1 (Drosophila)*	*NM_020651*	*PELI1*
53720_at	*2.33*	*3.18*		*NM_018381*	
209392_at	*2.33*	*2.29*	*ectonucleotide pyrophosphatase/phosphodiesterase 2 (autotaxin)*	*NM_001040092 /// NM_006209*	*ENPP2*

229280_s_at	2.33	2.31		XM_379398 /// XM_932815 /// XM_932820 /// XM_944201 /// XM_944203 /// XM_944207	
204567_s_at	2.33	1.78	ATP-binding cassette, sub-family G (WHITE), member 1	NM_004915 /// NM_016818 /// NM_207174 /// NM_207627 /// NM_207628 /// NM_207629 /// NM_207630	ABCG1
1561158_at	2.33	1.76			
1556462_a_at	2.33	1.95	Kruppel-like factor 12	NM_007249 ///	KLF12
222134_at	2.32	1.94	D-aspartate oxidase	NM_003649 /// NM_004032	DDO
213933_at	2.32	2.73	prostaglandin E receptor 3 (subtype EP3)	NM_000957 /// NM_198712 /// NM_198713 /// NM_198714 /// NM_198715 /// NM_198716 /// NM_198717 /// NM_198718 /// NM_198719	PTGER3
243902_at	2.32	2.10	SEC22 vesicle trafficking protein homolog B (S. cerevisiae)	NM_004892	SEC22B
212614_at	2.32	1.98	AT rich interactive domain 5B (MRF1-like)	NM_032199	ARID5B
209762_x_at	2.32	2.61	SP110 nuclear body protein	NM_004509 /// NM_004510 /// NM_080424	SP110
AFFX-HUMISGF3A/M97935_MB_at	2.32	3.44	signal transducer and activator of transcription 1, 91kDa	NM_007315 /// NM_139266	STAT1
202949_s_at	2.32	1.85	four and a half LIM domains 2	NM_001039492 /// NM_001450 /// NM_201555 /// NM_201557	FHL2
213358_at	2.32	1.74	KIAA0802	NM_001006114 /// NM_015210 /// NM_058181	KIAA0802
209118_s_at	2.32	2.21	tubulin, alpha 1a	NM_006009	TUBA1A
1570198_x_at	2.31	2.48	bone marrow stromal cell antigen 2	NM_004335	BST2

238583_at	*2.31*	*2.60*	*methionine sulfoxide reductase B3*	*NM_001031679 /// NM_198080*	*MSRB3*
208392_x_at	*2.31*	*3.15*	*SP110 nuclear body protein*	*NM_004509 /// NM_004510 /// NM_080424*	*SP110*
210145_at	*2.31*	*2.68*	*phospholipase A2, group IVA (cytosolic, calcium-dependent)*	*NM_024420*	*PLA2G4A*
239999_at	*2.28*	*1.77*	*chromosome 21 open reading frame 34*	*NM_001005732 /// NM_001005733 /// NM_001005734*	*C21orf34*
232803_at	*2.28*	*1.85*			
202820_at	*2.28*	*2.89*	*aryl hydrocarbon receptor*	*NM_001621*	*AHR*
44673_at	*2.28*	*1.87*	*sialic acid binding Ig-like lectin 1, sialoadhesin*	*NM_023068*	*SIGLEC1*
241497_at	*2.27*	*5.29*			
214651_s_at	*2.27*	*2.84*	*homeobox A9*	*NM_152739*	*HOXA9*
206181_at	*2.27*	*3.88*	*signaling lymphocytic activation molecule family member 1*	*NM_003037*	*SLAMF1*
214290_s_at	*2.27*	*2.01*		*NM_001040874 /// NM_003516 /// XM_928387*	
214688_at	*2.26*	*2.13*	*transducin-like enhancer of split 4 (E(sp1) homolog, Drosophila)*	*NM_007005*	*TLE4*
216813_at	*2.26*	*2.50*			
218429_s_at	*2.25*	*2.69*		*NM_018381*	
219049_at	*2.24*	*1.74*	*chondroitin sulfate N-acetylgalactosaminyltransferase 1*	*NM_018371*	*CSGALNACT1*
1559949_at	*2.23*	*2.71*	*trichorhinophalangeal syndrome I*	*NM_014112*	*TRPS1*
222146_s_at	*2.23*	*3.07*	*transcription factor 4*	*NM_003199*	*TCF4*
201015_s_at	*2.23*	*2.38*	*junction plakoglobin*	*NM_002230 /// NM_021991*	*JUP*
233152_x_at	*2.23*	*1.79*	*tankyrase, TRF1-interacting ankyrin-related ADP-ribose polymerase*	*NM_003747*	*TNKS*
209761_s_at	*2.21*	*3.07*	*SP110 nuclear body protein*	*NM_004509 /// NM_004510 /// NM_080424*	*SP110*
242197_x_at	*2.21*	*2.05*	*CD36 molecule*	*NM_000072 ///*	*CD36*
241238_at	*2.20*	*3.11*			

1554712_a_at	*2.20*	*4.17*	*glycine-N-*	*NM_145016*	*GLYATL2*
228108_at	*2.20*	*3.77*			
233465_at	*2.20*	*1.86*	*sprouty-related, EVH1*	*NM_152594*	*SPRED1*
223044_at	*2.20*	*1.77*	*solute carrier family 40 (iron-regulated transporter), member 1*	*NM_014585*	*SLC40A1*
241202_at	*2.19*	*2.10*	*UDP-N-acetyl-alpha-D-galactosamine:polypeptide N-acetylgalactosaminyltransferase 10 (GalNAc-T10)*	*NM_017540 /// NM_198321*	*GALNT10*
239587_at	*2.19*	*1.92*			
233887_at	*2.19*	*4.63*	*G protein-coupled receptor 126*	*NM_001032394 /// NM_001032395 /// NM_020455 /// NM_198569*	*GPR126*
234284_at	*2.18*	*2.86*	*guanine nucleotide binding protein (G protein), gamma 8*	*NM_033258*	*GNG8*
202074_s_at	*2.18*	*1.96*	*optineurin*	*NM_001008211 /// NM_001008212 /// NM_001008213 /// NM_021980*	*OPTN*
209806_at	*2.17*	*1.80*	*histone cluster 1, H2bk*	*NM_080593*	*HIST1H2BK*
235884_at	*2.17*	*1.74*			
226069_at	*2.17*	*7.59*	*prickle homolog 1 (Drosophila)*	*NM_153026*	*PRICKLE1*
210375_at	*2.17*	*2.50*	*prostaglandin E receptor 3 (subtype EP3)*	*NM_000957 /// NM_198712 /// NM_198713 /// NM_198714 /// NM_198715 /// NM_198716 /// NM_198717 /// NM_198718 /// NM_198719*	*PTGER3*
228678_at	*2.17*	*1.96*	*cytochrome P450, family 2, subfamily U, polypeptide 1*	*NM_001001794 /// NM_183075*	*CYP2U1*
1559633_a_at	*2.17*	*1.77*	*cholinergic receptor,*	*NM_000740*	*CHRM3*
213931_at	*2.16*	*1.77*	*inhibitor of DNA binding 2, dominant negative helix-loop-helix protein*	*NM_001039082 /// NM_002166*	*ID2*

217788_s_at	*2.16*	*1.95*	*UDP-N-acetyl-alpha-D-galactosamine:polypeptide N-acetylgalactosaminyltransferase 2 (GalNAc-T2)*	*NM_004481*	*GALNT2*
240633_at	*2.15*	*2.48*	*docking protein 7*	*NM_173660*	*DOK7*
226756_at	*2.15*	*2.87*			
1560396_at	*2.15*	*1.77*	*kelch-like 6 (Drosophila)*	*NM_130446*	*KLHL6*
201334_s_at	*2.14*	*1.99*	*Rho guanine nucleotide exchange factor (GEF) 12*	*NM_015313*	*ARHGEF12*
213261_at	*2.14*	*1.77*		*XM_047357 /// XM_940627*	
229956_at	*2.13*	*2.12*			
201315_x_at	*2.13*	*2.46*	*interferon induced transmembrane protein 2 (1-8D)*	*NM_006435*	*IFITM2*
209763_at	*2.12*	*2.60*	*chordin-like 1*	*NM_145234*	*CHRDL1*
1562484_at	*2.12*	*1.79*		*NM_001033659*	
241478_at	*2.12*	*1.86*	*MICAL-like 2*	*NM_024723 /// NM_182924*	*MICALL2*
208886_at	*2.12*	*2.22*	*H1 histone family, member 0*	*NM_005318*	*H1F0*
1294_at	*2.12*		*ubiquitin-like modifier*	*NM_003335*	*UBA7*
239307_at	*2.12*	*1.79*			
209327_s_at	*2.12*	*1.96*		*NM_016391*	
233880_at	*2.11*	*2.25*	*ring finger protein 213*	*NM_020914*	*RNF213*
232155_at	*2.11*	*2.71*	*KIAA1618*	*NM_020954*	*KIAA1618*
242681_at	*2.11*	*3.59*	*NAD(P)H dehydrogenase, quinone 1*	*NM_000903 /// NM_001025433 /// NM_001025434*	*NQO1*
213388_at	*2.11*	*2.59*	*phosphodiesterase 4D interacting protein (myomegalin)*	*NM_001002810 /// NM_001002811 /// NM_001002812 /// NM_014644 /// NM_022359*	*PDE4DIP*
203815_at	*2.11*	*1.89*	*glutathione S-transferase theta 1*	*NM_000853*	*GSTT1*
1557113_at	*2.11*	*2.51*			
217787_s_at	*2.10*	*2.33*	*UDP-N-acetyl-alpha-D-galactosamine:polypeptide N-acetylgalactosaminyltransferase 2 (GalNAc-T2)*	*NM_004481*	*GALNT2*

213069_at	*2.10*	*1.94*	*HEG homolog 1 (zebrafish)*	*XM_087386*	*HEG1*
241803_s_at	*2.10*	*1.94*			
222142_at	*2.09*	*3.23*	*cylindromatosis (turban tumor syndrome)*	*NM_001042355 /// NM_001042412 /// NM_015247*	*CYLD*
208782_at	*2.09*	*2.47*	*follistatin-like 1*	*NM_007085*	*FSTL1*
219383_at	*2.09*	*1.96*		*NM_024841*	
242405_at	*2.09*	*1.93*	*mastermind-like 2 (Drosophila)*	*NM_032427*	*MAML2*
236948_x_at	*2.09*	*2.74*	*splicing factor, arginine/serine-rich 11*	*NM_004768*	*SFRS11*
230906_at	*2.08*	*2.07*	*UDP-N-acetyl-alpha-D-galactosamine:polypeptide N-acetylgalactosaminyltransferase 10 (GalNAc-T10)*	*NM_017540 /// NM_198321*	*GALNT10*
204635_at	*2.08*	*2.13*	*ribosomal protein S6 kinase, 90kDa, polypeptide 5*	*NM_004755 /// NM_182398*	*RPS6KA5*
240271_at	*2.07*	*1.83*	*myotubularin related protein 3*	*NM_021090 /// NM_153050 /// NM_153051*	*MTMR3*
244404_at	*2.07*	*3.02*	*syntaxin binding protein 4*	*NM_178509*	*STXBP4*
233377_at	*2.07*	*2.64*	*AT rich interactive domain 5B (MRF1-like)*	*NM_032199*	*ARID5B*
243952_at	*2.07*	*2.25*			
226420_at	*2.07*	*2.86*	*ecotropic viral integration site 1*	*NM_005241*	*EVI1*
212822_at	*2.07*	*2.57*	*HEG homolog 1 (zebrafish)*	*XM_087386*	*HEG1*
238297_at	*2.07*	*2.16*	*phosphatase and actin regulator 1*	*NM_030948*	*PHACTR1*
225875_s_at	*2.06*	*1.77*	*NIPA-like domain containing 3*	*NM_020448*	*NPAL3*
1562403_a_at	*2.06*	*1.78*	*solute carrier family 8 (sodium-calcium exchanger), member 3*	*NM_033262 /// NM_058240 /// NM_182932 /// NM_182936 /// NM_183002*	*SLC8A3*
239600_at	*2.06*	*3.34*	*signal-induced proliferation-associated 1 like 1*	*NM_015556*	*SIPA1L1*
221286_s_at	*2.05*	*2.31*		*NM_016459*	
241736_at	*2.05*	*5.55*	*F-box and WD repeat*	*NM_012164*	*FBXW2*
203476_at	*2.05*	*3.55*	*trophoblast glycoprotein*	*NM_006670*	*TPBG*
225344_at	*2.05*	*2.25*	*nuclear receptor coactivator 7*	*NM_181782*	*NCOA7*

205801_s_at	2.05	2.02	RAS guanyl releasing protein 3 (calcium and DAG-regulated)	NM_170672	RASGRP3
200923_at	2.05	3.52	lectin, galactoside-binding, soluble, 3 binding protein	NM_005567	LGALS3BP
208891_at	2.04	1.97	dual specificity phosphatase 6	NM_001946 /// NM_022652	DUSP6
204546_at	2.04	3.59	KIAA0513	NM_014732	KIAA0513
203657_s_at	2.04	2.25	cathepsin F	NM_003793	CTSF
202270_at	2.04	2.24	guanylate binding protein 1, interferon-inducible, 67kDa	NM_002053	GBP1
230064_at	2.03	1.85		XM_926797	
203217_s_at	2.03	2.91	ST3 beta-galactoside alpha-2,3-sialyltransferase 5	NM_001042437 /// NM_003896	ST3GAL5
210084_x_at	2.03	1.91	tryptase alpha/beta 1	NM_003294	TPSAB1
231310_at	2.03	3.27			
212385_at	2.03	1.91			
1552323_s_at	2.03	2.73	family with sequence similarity 122C	NM_138819	FAM122C
204547_at	2.02	2.31	RAB40B, member RAS oncogene family	NM_006822	RAB40B
223000_s_at	2.02	1.78	F11 receptor	NM_016946 /// NM_144502 /// NM_144503 /// NM_144504	F11R
1554997_a_at	2.02	3.48	prostaglandin-endoperoxide synthase 2 (prostaglandin G/H synthase and cyclooxygenase)	NM_000963	PTGS2
204217_s_at	2.02	4.36	reticulon 2	NM_005619 /// NM_206900 /// NM_206901 /// NM_206902	RTN2
65438_at	2.02	2.18	KIAA1609	NM_020947	KIAA1609
231412_at	2.02	2.04			
202687_s_at	2.01	2.02	tumor necrosis factor (ligand) superfamily, member 10	NM_003810	TNFSF10
212276_at	2.01	2.09	lipin 1	NM_145693	LPIN1
207837_at	2.01	5.01	RNA binding protein with multiple splicing	NM_001008710 /// NM_001008711 /// NM_001008712 /// NM_006867	RBPMS
203281_s_at	2.01	2.22	ubiquitin-like modifier	NM_003335	UBA7
227235_at	2.01	1.88	guanylate cyclase 1, soluble, alpha 3	NM_000856	GUCY1A3

230708_at	*2.00*	*2.01*	*prickle homolog 1 (Drosophila)*	*NM_153026*	*PRICKLE1*
243476_at	*2.00*	*1.82*			
215288_at	*2.00*	*1.87*	*transient receptor potential cation channel,*	*NR_002720*	*TRPC2*
1570375_at	*2.00*	*2.57*	*protein tyrosine phosphatase, receptor type, N polypeptide 2*	*NM_002847 /// NM_130842 /// NM_130843*	*PTPRN2*
216278_at	*2.00*	*2.67*		*NM_014701*	
225273_at	*1.99*	*1.97*	*WWC family member 3*	*NM_015691*	*WWC3*
200884_at	*1.99*	*1.80*	*creatine kinase, brain*	*NM_001823*	*CKB*
208965_s_at	*1.99*	*2.69*	*interferon, gamma-inducible protein 16*	*NM_005531*	*IFI16*
220030_at	*1.99*	*4.72*	*serine/threonine/tyrosine kinase 1*	*NM_018423*	*STYK1*
1559375_s_at	*1.98*	*2.03*	*ATPase, H+ transporting, lysosomal V0 subunit a1*	*NM_005177*	*ATP6V0A1*
210377_at	*1.98*	*2.08*	*acyl-CoA synthetase medium-chain family member 3*	*NM_005622 /// NM_202000*	*ACSM3*
241446_at	*1.98*	*2.67*	*ADAM metallopeptidase domain 28*	*NM_014265 /// NM_021777*	*ADAM28*
215615_x_at	*1.98*	*3.18*	*arginine-glutamic acid dipeptide (RE) repeats*	*NM_001042681 /// NM_001042682 /// NM_012102*	*RERE*
238164_at	*1.98*	*1.77*	*USP6 N-terminal like*	*XM_374768 /// XM_927409 /// XM_932055 /// XM_938665 /// XM_943796 /// XM_943800*	*USP6NL*
232370_at	*1.97*	*2.25*			
1561627_at	*1.97*	*2.58*	*fragile X mental retardation 1*	*NM_002024*	*FMR1*
207056_s_at	*1.97*	*2.24*	*solute carrier family 4, sodium bicarbonate cotransporter, member 8*	*NM_001039960 /// NM_004858*	*SLC4A8*
242216_at	*1.96*	*1.86*	*DnaJ (Hsp40) homolog, subfamily C, member 1*	*NM_022365*	*DNAJC1*
226823_at	*1.96*	*2.14*	*phosphatase and actin regulator 4*	*NM_023923*	*PHACTR4*
235761_at	*1.96*	*1.80*			
243386_at	*1.96*	*3.97*		*NM_001039183*	
1570021_at	*1.96*	*2.55*			

204622_x_at	1.96	4.62	nuclear receptor subfamily 4, group A, member 2	NM_006186 /// NM_173171 /// NM_173172 /// NM_173173	NR4A2
215342_s_at	1.96	2.33	RAB GTPase activating protein 1-like	NM_001035230 /// NM_014857	RABGAP1L
211526_s_at	1.96	2.90	regulator of telomere	NM_016434 ///	RTEL1
235094_at	1.96	1.83	tropomyosin 4	NM_003290	TPM4
1568592_at	1.96	2.47	tripartite motif-containing 69	NM_080745 /// NM_182985	TRIM69
238771_at	1.96	2.32	fibrosin	NM_022452	FBRS
226677_at	1.95	1.75	zinc finger protein 521	NM_015461	ZNF521
240180_at	1.94	2.33			
223849_s_at	1.94	1.79	Mov10, Moloney leukemia virus 10, homolog (mouse)	NM_020963	MOV10
230624_at	1.94	2.09	solute carrier family 25, member 27	NM_004277	SLC25A27
209576_at	1.93	2.31	guanine nucleotide binding protein (G protein), alpha inhibiting activity polypeptide 1	NM_002069	GNAI1
235072_s_at	1.93	1.88			
207111_at	1.92	2.46	egf-like module containing, mucin-like, hormone receptor-like 1	NM_001974	EMR1
239278_at	1.92	2.80			
231557_at	1.92	8.72			
202708_s_at	1.92	2.91	histone cluster 2, H2be	NM_003528	HIST2H2BE
219304_s_at	1.92	1.79	platelet derived growth factor D	NM_025208 /// NM_033135	PDGFD
220777_at	1.92	3.06	kinesin family member 13A	NM_022113	KIF13A
241982_at	1.91	1.87	TAF1 RNA polymerase II, TATA box binding protein (TBP)-associated factor, 250kDa	NM_004606 /// NM_138923	TAF1
206965_at	1.91	2.26	Kruppel-like factor 12	NM_007249 /// NM_016285	KLF12
226382_at	1.91	2.25			
242414_at	1.91	1.92	quinolinate phosphoribosyltransferase (nicotinate-nucleotide pyrophosphorylase (carboxylating))	NM_014298	QPRT

242407_at	1.91	2.75	arginine-glutamic acid dipeptide (RE) repeats	NM_001042681 /// NM_001042682 /// NM_012102	RERE
223991_s_at	1.90	1.83	UDP-N-acetyl-alpha-D-galactosamine:polypeptide N-acetylgalactosaminyltransferase 2 (GalNAc-T2)	NM_004481	GALNT2
205888_s_at	1.90	1.96	myelin transcription factor 1-like	NM_014790 /// NM_015025	MYT1L
204755_x_at	1.90	1.77	hepatic leukemia factor	NM_002126	HLF
213982_s_at	1.90	1.76	RAB GTPase activating protein 1-like	NM_001035230 /// NM_014857	RABGAP1L
203569_s_at	1.90	1.88	oral-facial-digital syndrome 1	NM_003611	OFD1
1557718_at	1.90	1.80	protein phosphatase 2, regulatory subunit B', gamma isoform	NM_002719 /// NM_178586 /// NM_178587 /// NM_178588	PPP2R5C
204633_s_at	1.89	2.21	ribosomal protein S6 kinase, 90kDa, polypeptide 5	NM_004755 /// NM_182398	RPS6KA5
202255_s_at	1.89	2.37	signal-induced proliferation-associated 1 like 1	NM_015556	SIPA1L1
236565_s_at	1.89	2.04	La ribonucleoprotein domain family, member 6	NM_018357 /// NM_197958	LARP6
218718_at	1.89	1.96	platelet derived growth factor C	NM_016205	PDGFC
226808_at	1.89	1.81		XM_376720 /// XM_935653 /// XM_937178	
243928_s_at	1.88	1.95	ATP-binding cassette, sub-family C (CFTR/MRP), member 4	NM_005845	ABCC4
201649_at	1.88	2.01	ubiquitin-conjugating enzyme E2L 6	NM_004223 /// NM_198183	UBE2L6
202769_at	1.88	2.09	cyclin G2	NM_004354	CCNG2
212592_at	1.88	5.89	immunoglobulin J polypeptide, linker protein for immunoglobulin alpha and mu polypeptides	NM_144646	IGJ
232636_at	1.88	4.63	SLIT and NTRK-like family, member 4	NM_173078	SLITRK4

234276_at	*1.88*	*2.15*	*USP6 N-terminal like*	*XM_374768 /// XM_927409 /// XM_932055 /// XM_938665 /// XM_943796 /// XM_943800*	*USP6NL*
240399_at	*1.87*	*4.53*	*triple functional domain (PTPRF interacting)*	*NM_007118*	*TRIO*
1561763_at	*1.87*	*1.78*	*KIAA2026*	*NM_001017969*	*KIAA2026*
212256_at	*1.87*	*1.85*	*UDP-N-acetyl-alpha-D-galactosamine:polypeptide N-acetylgalactosaminyltransferase 10 (GalNAc-T10)*	*NM_017540 /// NM_198321*	*GALNT10*
212387_at	*1.87*	*1.86*			
1564468_at	*1.87*	*2.29*		*NM_017884*	
244716_x_at	*1.86*	*2.15*	*transmembrane and immunoglobulin domain containing 2*	*NM_144615*	*TMIGD2*
235538_at	*1.86*	*1.85*	*pre-B-cell leukemia homeobox 1*	*NM_002585*	*PBX1*
203372_s_at	*1.86*	*1.80*	*suppressor of cytokine signaling 2*	*NM_003877*	*SOCS2*
216474_x_at	*1.85*	*1.88*	*tryptase alpha/beta 1*	*NM_003294*	*TPSAB1*
213894_at	*1.85*	*2.14*	*thrombospondin, type I, domain containing 7A*	*XM_371877 /// XM_374404 /// XM_928187 /// XM_932697 /// XM_935904 /// XM_935905 /// XM_937164 /// XM_943202 /// XM_943205*	*THSD7A*
224701_at	*1.85*	*2.64*	*poly (ADP-ribose) polymerase family, member 14*	*NM_017554*	*PARP14*
202988_s_at	*1.85*	*4.87*	*regulator of G-protein signaling 1*	*NM_002922*	*RGS1*
1553155_x_at	*1.85*	*2.41*	*ATPase, H+ transporting, lysosomal 38kDa, V0 subunit d2*	*NM_152565*	*ATP6V0D2*
215017_s_at	*1.85*	*1.79*	*formin binding protein 1-like*	*NM_001024948 /// NM_017737*	*FNBP1L*
1563573_at	*1.85*	*2.02*	*Rho guanine nucleotide exchange factor (GEF) 3*	*NM_019555*	*ARHGEF3*
205942_s_at	*1.85*	*1.88*	*acyl-CoA synthetase medium-chain family member 3*	*NM_005622 /// NM_202000*	*ACSM3*

218501_at	*1.84*	*2.05*	*Rho guanine nucleotide exchange factor (GEF) 3*	*NM_019555*	*ARHGEF3*
239300_at	*1.84*	*1.90*	*phosphoinositide-3-kinase, class 3*	*NM_002647*	*PIK3C3*
206385_s_at	*1.84*	*3.50*	*ankyrin 3, node of Ranvier (ankyrin G)*	*NM_001149 /// NM_020987*	*ANK3*
221816_s_at	*1.84*	*1.88*		*NM_001017435 /// NM_001040443 /// NM_001040444 /// NM_152582*	
243277_x_at	*1.84*	*2.23*	*ecotropic viral integration site 1*	*NM_005241*	*EVI1*
202124_s_at	*1.83*	*1.89*	*trafficking protein, kinesin binding 2*	*NM_015049*	*TRAK2*
218085_at	*1.83*	*1.98*	*chromatin modifying protein 5*	*NM_016410*	*CHMP5*
209426_s_at	*1.83*	*1.84*	*alpha-methylacyl-CoA racemase*	*NM_014324 /// NM_203382*	*AMACR*
236285_at	*1.83*	*2.63*			
204612_at	*1.83*	*1.84*	*protein kinase (cAMP-dependent, catalytic) inhibitor alpha*	*NM_006823 /// NM_181839*	*PKIA*
207017_at	*1.83*	*3.01*	*RAB27B, member RAS oncogene family*	*NM_001001713 /// NM_004163 /// NM_007341*	*RAB27B*
229739_s_at	*1.83*	*2.42*	*family with sequence similarity 116, member B*	*NM_001001794*	*FAM116B*
201243_s_at	*1.83*	*2.19*	*ATPase, Na+/K+ transporting, beta 1 polypeptide*	*NM_001001787 /// NM_001677*	*ATP1B1*
215881_x_at	*1.83*	*3.49*	*synovial sarcoma, X breakpoint 3*	*NM_003147 /// NM_005636 /// NM_021014 /// NM_173358 /// NM_174962 /// NM_175698 /// NM_175711 /// NM_175729 /// XM_925889 /// XM_930735 /// XM_937466 /// XM_941508 /// XM_942177*	*SSX3*
44790_s_at	*1.83*	*4.69*	*chromosome 13 open reading frame 18*	*NM_025113*	*C13orf18*
227243_s_at	*1.83*	*2.96*	*early B-cell factor 3*	*NM_001005463*	*EBF3*

228003_at	1.81	1.85	RAB30, member RAS oncogene family	NM_014488	RAB30
241421_at	1.81	2.34			
203921_at	1.81	2.11	carbohydrate (N-acetylglucosamine-6-O) sulfotransferase 2	NM_004267	CHST2
202808_at	1.80	1.81			
209636_at	1.80	6.14	nuclear factor of kappa light polypeptide gene enhancer in B-cells 2 (p49/p100)	NM_002502	NFKB2
212599_at	1.80	2.13	autism susceptibility candidate 2	NM_015570	AUTS2
1565723_at	1.80	1.80	LSM domain containing 1	NM_032356	LSMD1
1554335_at	1.80	2.12	pleckstrin homology, Sec7 and coiled-coil domains 4	NM_013385	PSCD4
237124_at	1.79	2.59			
1560994_x_at	1.79	2.83		XR_000581 /// XR_000654	
1564567_at	1.79	2.14			
204530_s_at	1.79	2.81	thymocyte selection-associated high mobility group box	NM_014729	TOX
229530_at	1.79	1.80	guanylate cyclase 1, soluble, alpha 3	NM_000856	GUCY1A3
241216_at	1.79	3.12	kinesin family member 1B	NM_015074 /// NM_183416	KIF1B
229506_at	1.78	2.43			
214860_at	1.78	2.82	solute carrier family 9 (sodium/hydrogen exchanger), member 7	NM_032591	SLC9A7
205091_x_at	1.78	1.80	RecQ protein-like (DNA helicase Q1-like)	NM_002907 /// NM_032941	RECQL
1555122_at	1.78	2.16	G protein-coupled receptor 125	NM_145290	GPR125
241762_at	1.77	32.63	F-box protein 32	NM_058229 /// NM_148177	FBXO32
226675_s_at	1.77	1.76	metastasis associated lung adenocarcinoma transcript 1 (non-protein coding)	NR_002819	MALAT1
212045_at	1.77	1.79	golgi apparatus protein 1	NM_012201	GLG1
1569540_at	1.77	2.01			
202889_x_at	1.77	1.83	microtubule-associated protein 7	NM_003980	MAP7

244689_at	1.76	2.25	peroxisome proliferator-activated receptor alpha	NM_001001928 /// NM_001001929 /// NM_001001930 /// NM_005036 /// NM_032644	PPARA
1553611_s_at	1.76	3.32		NM_001039548	
241818_at	1.76	2.47	cadherin 3, type 1, P-cadherin (placental)	NM_133458	CDH3
201468_s_at	1.76	1.84	NAD(P)H dehydrogenase, quinone 1	NM_000903 /// NM_001025433 /// NM_001025434	NQO1
202600_s_at	1.76	1.75	nuclear receptor interacting protein 1	NM_003489	NRIP1
201418_s_at	1.76	1.77	SRY (sex determining region Y)-box 4	NM_003107	SOX4
243463_s_at	1.76	1.81	Ras-like without CAAX 1	NM_006912	RIT1
1561310_at	1.75	2.23			
241750_x_at	1.75	1.79	USP6 N-terminal like	XM_374768 /// XM_927409 /// XM_932055 /// XM_938665 /// XM_943796 /// XM_943800	USP6NL
209060_x_at	1.75	2.14	nuclear receptor coactivator 3	NM_006534 /// NM_181659	NCOA3
1556682_s_at	1.75	3.03	autism susceptibility	NM_015570	AUTS2
228243_at	1.75	2.04	PAX interacting (with transcription-activation domain) protein 1	NM_007349	PAXIP1
205411_at	1.75	1.97	serine/threonine kinase 4	NM_006282	STK4
223595_at	1.75	7.26	transmembrane protein 133	NM_032021	TMEM133
244611_at	1.75	1.74	mediator complex subunit 13	NM_005121	MED13
224378_x_at	1.75	1.95	microtubule-associated protein 1 light chain 3 alpha	NM_032514 /// NM_181509	MAP1LC3A
235508_at	1.75	1.91	promyelocytic leukemia	NM_002675 /// NM_033238 /// NM_033239 /// NM_033240 /// NM_033244 /// NM_033246 /// NM_033247 /// NM_033249 /// NM_033250	PML

226029_at	*1.75*	*4.33*	*vang-like 2 (van gogh, Drosophila)*	*NM_020335*	*VANGL2*
235173_at	*1.74*	*1.87*		*XM_379228 /// XM_944985*	
213665_at	*1.74*	*2.69*	*SRY (sex determining region Y)-box 4*	*NM_003107*	*SOX4*
208268_at	*1.74*	*2.70*	*ADAM metallopeptidase domain 28*	*NM_014265 /// NM_021777*	*ADAM28*
237100_at	*1.74*	*2.50*	*janus kinase and microtubule interacting protein 2*	*NM_014790*	*JAKMIP2*
216129_at	*1.74*	*2.58*	*ATPase, class II, type 9A*	*NM_006045*	*ATP9A*
1554768_a_at	*-1.74*	*-2.12*	*MAD2 mitotic arrest*	*NM_002358*	*MAD2L1*
217948_at	*-1.74*	*-4.36*			
241846_at	*-1.75*	*-2.05*	*HLA complex group 18*	*XM_933653 /// XM_936164 /// XM_944855*	*HCG18*
1554892_a_at	*-1.75*	*-2.68*	*membrane-spanning 4-domains, subfamily A,*	*NM_001031666 ///*	*MS4A3*
1553849_at	*-1.76*	*-2.23*	*coiled-coil domain containing 26*		*CCDC26*
1562940_at	*-1.76*	*-3.50*			
230266_at	*-1.77*	*-1.85*	*RAB7B, member RAS oncogene family*	*NM_177403*	*RAB7B*
1569189_at	*-1.78*	*-2.60*	*tetratricopeptide repeat domain 9C*	*NM_173810*	*TTC9C*
222462_s_at	*-1.78*	*-1.75*	*beta-site APP-cleaving enzyme 1*	*NM_012104 /// NM_138971 /// NM_138972 /// NM_138973*	*BACE1*
244049_at	*-1.78*	*-1.75*			
220926_s_at	*-1.80*	*-1.88*		*NM_025191 /// XM_930112 /// XM_940489*	
218445_at	*-1.80*	*-2.04*	*H2A histone family, member Y2*	*NM_018649*	*H2AFY2*
211745_x_at	*-1.80*	*-1.87*	*hemoglobin, alpha 1*	*NM_000558*	*HBA1*
204446_s_at	*-1.80*	*-2.48*	*arachidonate 5-lipoxygenase*	*NM_000698*	*ALOX5*
209201_x_at	*-1.81*	*-1.92*	*chemokine (C-X-C motif) receptor 4*	*NM_001008540 /// NM_003467*	*CXCR4*
1553725_s_at	*-1.81*	*-1.88*	*zinc finger protein 644*	*NM_016620 ///*	*ZNF644*
213744_at	*-1.83*	*-2.85*	*attractin-like 1*	*NM_207303*	*ATRNL1*
223452_s_at	*-1.84*	*-1.81*		*NM_015459*	
1554676_at	*-1.84*	*-2.04*	*serglycin*	*NM_002727*	*SRGN*
209458_x_at	*-1.84*	*-1.99*	*hemoglobin, alpha 1*	*NM_000517 /// NM_000558*	*HBA1*
214627_at	*-1.84*	*-2.74*	*eosinophil peroxidase*	*NM_000502*	*EPX*

212478_at	*-1.86*	*-1.94*	*required for meiotic nuclear division 5 homolog A (S. cerevisiae)*	*NM_022780*	*RMND5A*
217369_at	*-1.86*	*-14.55*	*immunoglobulin heavy constant gamma 1 (G1m marker)*	*XM_370973*	*IGHG1*
230645_at	*-1.86*	*-2.01*	*FERM domain containing 3*	*NM_174938*	*FRMD3*
206034_at	*-1.86*	*-2.37*	*serpin peptidase inhibitor, clade B (ovalbumin), member 8*	*NM_001031848 /// NM_002640 /// NM_198833*	*SERPINB8*
238596_at	*-1.87*	*-1.84*	*chromosome 10 open reading frame 4*	*NM_145246 /// NM_203438 /// NM_203439 /// NM_203440 /// NM_203441*	*C10orf4*
231234_at	*-1.87*	*-2.07*	*cathepsin C*	*NM_001814 /// NM_148170*	*CTSC*
1569362_at	*-1.88*	*-2.81*	*activated leukocyte cell*	*NM_001627*	*ALCAM*
1569114_at	*-1.89*	*-1.83*			
217414_x_at	*-1.89*	*-1.85*	*hemoglobin, alpha 1*	*NM_000517 /// NM_000558*	*HBA1*
205786_s_at	*-1.89*	*-1.75*	*integrin, alpha M (complement component 3 receptor 3 subunit)*	*NM_000632*	*ITGAM*
229485_x_at	*-1.89*	*-3.19*	*shisa homolog 3 (Xenopus laevis)*	*XM_496688 /// XM_941105*	*SHISA3*
206277_at	*-1.90*	*-1.87*	*purinergic receptor P2Y, G-protein coupled, 2*	*NM_002564 /// NM_176071 /// NM_176072*	*P2RY2*
224799_at	*-1.90*	*-2.82*	*Nedd4 family interacting protein 2*	*NM_019080*	*NDFIP2*
1558230_at	*-1.91*	*-1.75*	*splicing factor 3b, subunit 2, 145kDa*	*NM_006842*	*SF3B2*
1569629_x_at	*-1.91*	*-2.04*		*XM_928240 /// XM_942518*	
214414_x_at	*-1.92*	*-2.26*	*hemoglobin, alpha 2*	*NM_000517*	*HBA2*
213174_at	*-1.92*	*-1.86*	*tetratricopeptide repeat domain 9*	*XM_027236 /// XM_938197*	*TTC9*
207219_at	*-1.92*	*-1.99*	*zinc finger protein 643*	*NM_023070*	*ZNF643*
1556735_at	*-1.93*	*-8.56*			
234624_at	*-1.93*	*-2.03*	*HERV-H LTR-associating 2*	*NM_007072*	*HHLA2*
230170_at	*-1.93*	*-1.88*	*oncostatin M*	*NM_020530*	*OSM*
236199_at	*-1.93*	*-2.79*	*arachidonate 5-lipoxygenase*	*NM_000698*	*ALOX5*
201404_x_at	*-1.96*	*-1.91*	*proteasome (prosome, macropain) subunit, beta type, 2*	*NM_002794*	*PSMB2*

235220_at	-1.97	-1.78	Yip1 domain family, member 4	NM_032312	YIPF4
242170_at	-1.97	-2.68	zinc finger protein 154		ZNF154
223758_s_at	-1.98	-1.85	general transcription factor IIH, polypeptide 2, 44kDa	NM_001515	GTF2H2
1557775_a_at	-1.98	-3.48	RAN binding protein 17	NM_022897	RANBP17
236655_at	-1.99	-2.01	tumor protein D52	NM_001025252 /// NM_001025253 /// NM_005079	TPD52
1558807_at	-1.99	-2.25	ATPase family, AAA	XM_039676 ///	ATAD2B
229352_at	-1.99	-16.50	NADPH oxidase, EF-hand calcium binding domain 5	NM_145658	NOX5
213561_at	-2.01	-1.88	ASF1 anti-silencing function 1 homolog A (S. cerevisiae)	NM_014034	ASF1A
1554367_at	-2.01	-2.10	family with sequence similarity 26, member C	NM_001001412	FAM26C
231992_x_at	-2.02	-1.82		NR_002933	
234807_x_at	-2.03	-1.97			
205919_at	-2.05	-5.67	hemoglobin, epsilon 1	NM_005330	HBE1
217364_x_at	-2.05	-1.95	eukaryotic translation initiation factor 3, subunit J	NM_003758	EIF3J
1560001_at	-2.06	-28.04		NM_001012988	
214575_s_at	-2.06	-1.94	azurocidin 1 (cationic antimicrobial protein 37)	NM_001700	AZU1
238407_at	-2.07	-4.82	capping protein (actin filament) muscle Z-line, alpha 1	NM_006135	CAPZA1
1563445_x_at	-2.07	-2.07	cathepsin L-like 3		CTSLL3
220859_at	-2.08	-2.77			
211298_s_at	-2.08	-9.06	albumin	NM_000477	ALB
238748_at	-2.09	-9.20	RAD18 homolog (S. cerevisiae)	NM_020165	RAD18
1555963_x_at	-2.09	-2.17	UDP-GlcNAc:betaGal beta-1,3-N-acetylglucosaminyltransferase 7	NM_145236	B3GNT7
212009_s_at	-2.10	-1.83	stress-induced-phosphoprotein 1 (Hsp70/Hsp90-organizing protein)	NM_006819	STIP1
1560774_at	-2.10	-1.91	ribosomal protein SA	NM_001012321 /// NM_002295	RPSA

230931_at	-2.12	-26.11			
210870_s_at	-2.13	-2.66	epilepsy, progressive myoclonus type 2A, Lafora disease (laforin)	NM_001018041 /// NM_005670	EPM2A
226171_at	-2.15	-1.92		XM_933464 /// XM_944968	
230147_at	-2.15	-2.62	coagulation factor II (thrombin) receptor-like 2	NM_004101	F2RL2
244009_at	-2.16	-17.43	calcium modulating ligand	NM_001745	CAMLG
217696_at	-2.17	-1.96	fucosyltransferase 7 (alpha (1,3) fucosyltransferase)	NM_004479	FUT7
221212_x_at	-2.22	-1.74	polybromo 1	NM_018165 /// NM_018313 /// NM_181042	PBRM1
1567081_x_at	-2.24	-1.89	calmodulin-like 4	NM_017882	CALML4
232589_at	-2.24	-2.09			
230659_at	-2.25	-2.00	ER degradation enhancer, mannosidase alpha-like 1	NM_014674	EDEM1
1559293_x_at	-2.28	-1.85	chromosome 9 open reading frame 14	XM_376821 /// XM_938938	C9orf14
214366_s_at	-2.29	-2.95	arachidonate 5-lipoxygenase	NM_000698	ALOX5
242630_at	-2.30	-1.83	coiled-coil domain containing 138	NM_144978	CCDC138
212230_at	-2.30	-2.42	phosphatidic acid phosphatase type 2B	NM_003713 /// NM_177414	PPAP2B
1557703_at	-2.31	-4.22	membrane-associated ring finger (C3HC4) 7	NM_022826	7-Mar
1558014_s_at	-2.34	-2.20	male sterility domain	NM_032228	MLSTD2
228268_at	-2.35	-2.46	flavin containing monooxygenase 2 (non-functional)	NM_001460	FMO2
216063_at	-2.36	-3.07	hemoglobin, beta pseudogene 1	NR_001589	HBBP1
208116_s_at	-2.41	-1.84	mannosidase, alpha, class 1A, member 1	NM_005907	MAN1A1
206871_at	-2.46	-2.42	elastase 2, neutrophil	NM_001972	ELA2
207247_s_at	-2.47	-2.09	zinc finger protein, X-linked	NM_003410 /// NM_003411	ZFX
243948_at	-2.56	-2.43			
1562904_s_at	-2.58	-1.91		XM_496963 ///	
1555970_at	-2.59	-2.15			
204848_x_at	-2.60	-2.28	hemoglobin, gamma A	NM_000184 /// NM_000559	HBG1
204419_x_at	-2.63	-2.31	hemoglobin, gamma A	NM_000184 /// NM_000559	HBG1
1555775_a_at	-2.65	-1.76	zygote arrest 1	NM_175619 ///	ZAR1

214462_at	-2.71	-1.91	suppressor of cytokine signaling 6	NM_004232	SOCS6
221634_at	-2.73	-1.82	ribosomal protein L23a pseudogene 7	NR_000029	RPL23AP7
241060_x_at	-2.75	-1.93	tetraspanin 5	NM_005723	TSPAN5
213515_x_at	-2.76	-2.02	hemoglobin, gamma A	NM_000184 /// NM_000559	HBG1
214539_at	-2.79	-6.70	serpin peptidase inhibitor, clade B (ovalbumin), member 10	NM_005024	SERPINB10
219915_s_at	-2.91	-1.80	solute carrier family 16, member 10 (aromatic amino acid transporter)	NM_018593	SLC16A10
1552910_at	-2.94	-2.71	sialic acid binding Ig-like lectin 11	NM_052884	SIGLEC11
206390_x_at	-3.01	-3.06	platelet factor 4 (chemokine (C-X-C motif) ligand 4)	NM_002619	PF4
220811_at	-3.22	-20.39	proteoglycan 3	NM_006093	PRG3
235754_at	-3.34	-3.42	hemochromatosis	NM_000410 /// NM_139002 /// NM_139003 /// NM_139004 /// NM_139005 /// NM_139006 /// NM_139007 /// NM_139008 /// NM_139009 /// NM_139010 /// NM_139011	HFE
242753_x_at	-3.37	-2.46	chromosome 4 open reading frame 16	NM_018569	C4orf16
242284_at	-3.50	-3.48		XM_378866 /// XM_945515	
205987_at	-3.62	-2.15	CD1c molecule	NM_001765	CD1C
207717_s_at	-3.72	-3.22	plakophilin 2	NM_001005242 /// NM_004572	PKP2
215784_at	-3.87	-1.85	CD1e molecule	NM_001042583 /// NM_001042584 /// NM_001042585 /// NM_001042586 /// NM_001042587 /// NM_030893	CD1E
239751_at	-3.91	-2.57			
220726_at	-3.97	-3.16			

205863_at	*-4.02*	*-2.46*	*S100 calcium binding protein A12*	*NM_005621*	*S100A12*
241005_at	*-4.29*	*-7.03*			
226188_at	*-4.34*	*-2.51*		*NM_014181*	
240609_at	*-4.48*	*-1.95*			
206682_at	*-4.69*	*-1.99*	*C-type lectin domain*	*NM_006344 ///*	*CLEC10A*
242618_at	*-4.76*	*-2.09*	*HLA complex group 18*	*XM_933653 /// XM_936164 /// XM_944855*	*HCG18*
206647_at	*-4.84*	*-27.94*	*hemoglobin, zeta*	*NM_005332*	*HBZ*
216317_x_at	*-5.39*	*-8.92*	*Rh blood group, CcEe antigens*	*NM_020485 /// NM_138616 /// NM_138617 /// NM_138618*	*RHCE*
222943_at	*-5.50*	*-14.07*	*glucosidase, beta, acid 3 (cytosolic)*	*NM_020973*	*GBA3*
237835_at	*-5.56*	*-3.11*			
217537_x_at	*-5.56*	*-2.98*			
215415_s_at	*-5.66*	*-1.78*	*lysosomal trafficking regulator*	*NM_000081 /// NM_001005736*	*LYST*
207096_at	*-6.31*	*-1.78*	*serum amyloid A4, constitutive*	*NM_006512*	*SAA4*
241104_at	*-6.41*	*-8.64*	*sorbin and SH3 domain containing 2*	*NM_003603 /// NM_021069*	*SORBS2*
232546_at	*-6.85*	*-2.03*	*tumor protein p73*	*NM_005427*	*TP73*
230503_at	*-7.69*	*-1.80*	*sterile alpha motif domain containing 4A*	*NM_015589*	*SAMD4A*
226697_at	*-9.76*	*-2.61*	*family with sequence similarity 114, member A1*	*NM_138389*	*FAM114A1*
1555340_x_at	*-10.49*	*-11.86*	*RAP1A, member of RAS*	*NM_001010935*	*RAP1A*
211551_at	*-10.70*	*-8.95*	*epidermal growth factor receptor (erythroblastic leukemia viral (v-erb-b) oncogene homolog, avian)*	*NM_005228 /// NM_201282 /// NM_201283 /// NM_201284*	*EGFR*
244762_at	*-10.71*	*-2.10*	*Down syndrome critical region gene 3*	*NM_006052*	*DSCR3*
1555339_at	*-11.40*	*-13.48*	*RAP1A, member of RAS*	*NM_001010935*	*RAP1A*
242860_at	*-13.59*	*-1.81*			
224533_s_at	*-14.25*	*-4.84*			
1561340_at	*-16.13*	*-1.89*			
1569786_at	*-45.59*	*-4.94*			
