# Supplementary material for: Dissection of the Transformation of Primary Human Hematopoietic Cells by the Oncogene NUP98-HOXA9
Source: PLoS One. 2009 Aug 21;4(8):e6719. doi: 10.1371/journal.pone.0006719 (PMC2725295; doi:10.1371/journal.pone.0006719)
Supplement: Table S3 — Genes dysregulated by NUP98-HOXA9/N51S. Primary human CD34+ cells were retrovirally transduced with either control MSCV-IRES-GFP vector or vector expressing NUP98-HOXA9/N51S. Cells were sorted for GFP positivity and total RNA was subjected to microarray analysis. The experiment was performed two independent times and only genes that showed up- or down-regulation by 1.74 fold or more compared to control in both experiments were considered dysregulated. (0.02 MB PDF) [file pone.0006719.s003.pdf]

Table S3. Genes dysregulated by NUP98-HOXA9/N51S

Probeset I.D.	Fold Change		Gene Name	Accession Number	Gene Symbol
	Exp.1	*Exp.2*			
241676_x_at	*35.43*	*4.27*			
1565920_at	*17.29*	*2.42*			
221577_x_at	*15.65*	*3.15*	*growth differentiation factor 15*	*NM_004864*	*GDF15*
227834_at	*14.84*	*70.14*	*taxilin beta*	*NM_153235*	*TXLNB*
240878_at	*12.89*	*7.20*	*fibroblast growth factor 11*	*NM_199339*	*FGF11*
1558404_at	*9.67*	*4.75*		*XM_927425 /// XM_939517 /// XM_939537*	
209841_s_at	*7.13*	*2.65*	*leucine rich repeat neuronal 3*	*NM_018334*	*LRRN3*
211341_at	*6.26*	*1.84*	*POU class 4 homeobox 1*	*NM_006237*	*POU4F1*
236892_s_at	*6.03*	*2.48*			
1557647_a_at	*5.75*	*2.29*			
1556842_at	*5.14*	*2.15*			
207589_at	*4.71*	*2.79*	*adrenergic, alpha-1B-, receptor*	*NM_000679*	*ADRA1B*
226279_at	*4.23*	*1.96*	*protease, serine, 23*	*NM_007173*	*PRSS23*
229309_at	*4.11*	*2.57*			
224156_x_at	*4.06*	*4.07*	*interleukin 17 receptor B*	*NM_018725*	*IL17RB*
1560723_at	*3.81*	*3.58*			
234520_at	*3.71*	*14.41*			
1569551_at	*3.66*	*2.41*	*GRAM domain containing 1C*	*NM_017577*	*GRAMD1C*
204748_at	*3.62*	*4.16*	*prostaglandin-endoperoxide synthase 2 (prostaglandin G/H synthase and cyclooxygenase)*	*NM_000963*	*PTGS2*
231909_x_at	*3.61*	*1.74*	*outer dense fiber of sperm tails 2-like*	*NM_001007022 /// NM_020729*	*ODF2L*
209870_s_at	*3.52*	*2.10*	*amyloid beta (A4) precursor protein-binding, family A, member 2 (X11-like)*	*NM_005503*	*APBA2*
233701_at	*3.38*	*1.92*	*neuropilin 1*	*NM_001024628 /// NM_001024629 /// NM_003873*	*NRP1*
223900_s_at	*3.34*	*3.27*	*polybromo 1*	*NM_018165 /// NM_018313 /// NM_181042*	*PBRM1*

229756_at	3.32	2.51	inhibitor of DNA binding 2, dominant negative helix-loop-helix protein	NM_002166	ID2
1561486_at	3.29	1.89			
206962_x_at	3.23	6.27			
216692_at	3.20	2.34	zinc finger protein 337	NM_015655	ZNF337
1554997_a_at	3.13	4.59	prostaglandin-endoperoxide synthase 2 (prostaglandin G/H synthase and cyclooxygenase)	NM_000963	PTGS2
211269_s_at	3.10	2.60	interleukin 2 receptor, alpha	NM_000417	IL2RA
225056_at	3.08	2.32	signal-induced proliferation-associated 1 like 2	NM_020808	SIPA1L2
231473_at	3.02	3.22			
233944_at	2.97	4.36	contactin associated protein-like 2	NM_014141	CNTNAP2
232499_at	2.90	1.76	inositol 1,4,5-trisphosphate 3-kinase B	NM_002221	ITPKB
238301_at	2.87	2.36			
215368_at	2.86	2.58	nebulin	NM_004543	NEB
231804_at	2.81	2.15	relaxin/insulin-like family peptide receptor 1	NM_021634	RXFP1
223884_at	2.75	1.80	opticin	NM_014359	OPTC
206401_s_at	2.69	2.84	microtubule-associated protein tau	NM_005910 /// NM_016834 /// NM_016835 /// NM_016841	MAPT
205600_x_at	2.63	1.88	homeobox B5	NM_002147	HOXB5
238733_at	2.59	2.74	carboxypeptidase M	NM_001005502 /// NM_001874 /// NM_198320	CPM
1561405_s_at	2.59	1.98	cation channel, sperm associated 2	NM_054020 /// NM_172095 /// NM_172097	CATSPER2
208436_s_at	2.56	2.02	interferon regulatory factor 7	NM_001572 /// NM_004029 /// NM_004031	IRF7
204622_x_at	2.56	2.77	nuclear receptor subfamily 4, group A, member 2	NM_006186 /// NM_173171 /// NM_173172 /// NM_173173	NR4A2
213010_at	2.55	2.32	protein kinase C, delta binding protein	NM_145040	PRKCDBP
205749_at	2.55	2.31	cytochrome P450, family 1, subfamily A, polypeptide 1	NM_000499	CYP1A1
1561740_at	2.54	2.23			
227174_at	2.54	2.89	WD repeat domain 72	NM_182758	WDR72

216823_at	2.52	1.94		XM_495839 /// XM_926528 /// XM_931362 /// XM_937069 /// XM_943171 /// XM_943177	
219049_at	2.51	4.00	chondroitin sulfate N-acetylgalactosaminyltransferase 1	NM_018371	CSGALNACT1
222227_at	2.45	3.94	zinc finger protein 236	NM_007345	ZNF236
204811_s_at	2.44	3.23	calcium channel, voltage-dependent, alpha 2/delta subunit 2	NM_001005505 /// NM_006030	CACNA2D2
1552669_at	2.44	7.88	protein phosphatase 1, regulatory (inhibitor) subunit 3B	NM_024607	PPP1R3B
202284_s_at	2.41	2.87	cyclin-dependent kinase inhibitor 1A (p21, Cip1)	NM_000389 /// NM_078467	CDKN1A
239036_at	2.40	1.85	eukaryotic translation initiation factor 2C, 1	NM_012199	EIF2C1
242002_at	2.38	1.95	Na+/K+ transporting ATPase interacting 2	NM_001040214	NKAIN2
228766_at	2.35	1.86			
203158_s_at	2.32	2.05	glutaminase	NM_014905	GLS
214180_at	2.30	1.77	mannosidase, alpha, class 1C, member 1	NM_020379	MAN1C1
1555122_at	2.28	2.05	G protein-coupled receptor 125	NM_145290	GPR125
226232_at	2.22	1.89			
1552715_a_at	2.22	4.34	relaxin/insulin-like family peptide receptor 1	NM_021634	RXFP1
220014_at	2.21	2.16	proline rich 16	NM_016644	PRR16
244235_at	2.18	2.07	influenza virus NS1A binding protein	NM_006469 /// NM_016389	IVNS1ABP
230594_at	2.18	3.06	leucine-rich PPR-motif containing	NM_133259	LRPPRC
211000_s_at	2.17	1.79	interleukin 6 signal transducer (gp130, oncostatin M receptor)	NM_002184 /// NM_175767	IL6ST
242805_at	2.16	1.99	cyclin C	NM_001013399 /// NM_005190	CCNC
227553_at	2.16	2.77	nudE nuclear distribution gene E homolog (A. nidulans)-like 1	NM_001025579 /// NM_030808	NDEL1
217581_at	2.13	24.28	pyruvate kinase, muscle	NM_002654 /// NM_182470 /// NM_182471	PKM2
204439_at	2.13	6.80	interferon-induced protein 44-like	NM_006820	IFI44L

211597_s_at	*2.13*	*3.53*	*HOP homeobox*	*NM_032495 /// NM_139211 /// NM_139212*	*HOPX*
240728_at	*2.12*	*4.06*	*phospholipase C, beta 4*	*NM_000933 /// NM_182797*	*PLCB4*
242134_at	*2.11*	*2.16*			
214688_at	*2.09*	*2.82*	*transducin-like enhancer of split 4 (E(sp1) homolog, Drosophila)*	*NM_007005*	*TLE4*
1568889_at	*2.09*	*1.76*	*Fanconi anemia,*	*NM_001018115*	*FANCD2*
241604_at	*2.09*	*6.31*	*ATPase, class VI, type 11A*	*NM_015205 /// NM_032189*	*ATP11A*
214888_at	*2.08*	*2.58*	*calpain 2, (m/II) large subunit*	*NM_001748*	*CAPN2*
209555_s_at	*2.07*	*2.42*	*CD36 molecule (thrombospondin receptor)*	*NM_000072 /// NM_001001547 /// NM_001001548*	*CD36*
218675_at	*2.07*	*3.04*	*solute carrier family 22, member 17*	*NM_016609 /// NM_020372*	*SLC22A17*
230011_at	*2.06*	*2.14*	*meiosis inhibitor 1*	*NM_152513*	*MEI1*
211722_s_at	*2.06*	*1.99*	*histone deacetylase 6*	*NM_006044*	*HDAC6*
233587_s_at	*2.06*	*2.31*	*signal-induced proliferation-associated 1 like 2*	*NM_020808*	*SIPA1L2*
1569688_at	*2.05*	*2.07*	*flavin containing monooxygenase 5*	*NM_001461*	*FMO5*
224895_at	*2.05*	*3.37*	*Yes-associated protein 1, 65kDa*	*NM_006106*	*YAP1*
231412_at	*2.05*	*1.84*			
219434_at	*2.04*	*2.81*	*triggering receptor expressed on myeloid cells 1*	*NM_018643*	*TREM1*
211753_s_at	*2.04*	*2.47*	*relaxin 1*	*NM_006911*	*RLN1*
1556212_x_at	*2.03*	*1.94*		*XM_929541 /// XM_941868*	
1563166_at	*2.02*	*1.96*	*geranylgeranyl diphosphate synthase 1*	*NM_001037277 /// NM_001037278 /// NM_004837*	*GGPS1*
236799_at	*2.01*	*1.89*		*NM_020961*	
221696_s_at	*2.01*	*1.86*	*serine/threonine/tyrosine kinase 1*	*NM_018423*	*STYK1*
243846_x_at	*2.01*	*1.96*		*XM_926804*	
1561328_at	*1.99*	*11.50*			
233541_at	*1.97*	*2.51*		*XM_934514 /// XM_934516 /// XM_945782 /// XM_945783*	

230942_at	*1.96*	*1.93*	*CKLF-like MARVEL transmembrane domain containing 5*	*NM_001037288 /// NM_138460*	*CMTM5*
1553080_at	*1.94*	*9.88*	*casein alpha s2-like A*	*XM_379270 /// XM_934777 /// XM_943519 /// XM_943523*	*CSN1S2A*
221679_s_at	*1.92*	*2.11*	*abhydrolase domain containing 6*	*NM_020676*	*ABHD6*
206341_at	*1.91*	*2.10*	*interleukin 2 receptor, alpha*	*NM_000417*	*IL2RA*
207486_x_at	*1.90*	*3.50*	*chimerin (chimaerin) 2*	*NM_001039936 /// NM_004067*	*CHN2*
44783_s_at	*1.88*	*2.60*	*hairy/enhancer-of-split related with YRPW motif 1*	*NM_001040708 /// NM_012258*	*HEY1*
1559573_at	*1.86*	*2.15*			
211959_at	*1.85*	*1.82*	*insulin-like growth factor binding protein 5*	*NM_000599*	*IGFBP5*
206932_at	*1.85*	*3.33*	*cholesterol 25-hydroxylase*	*NM_003956*	*CH25H*
224240_s_at	*1.85*	*1.97*	*chemokine (C-C motif) ligand 28*	*NM_148672*	*CCL28*
226029_at	*1.85*	*2.59*	*vang-like 2 (van gogh, Drosophila)*	*NM_020335*	*VANGL2*
240038_at	*1.84*	*2.79*	*elongation factor, RNA polymerase II, 2*	*NM_012081*	*ELL2*
210200_at	*1.84*	*1.78*	*WW domain containing E3 ubiquitin protein ligase 2*	*NM_007014 /// NM_199423 /// NM_199424*	*WWP2*
234486_at	*1.84*	*2.15*	*olfactory receptor, family 51, subfamily B, member 2*	*NM_033180*	*OR51B2*
235684_s_at	*1.83*	*1.75*	*sestrin 3*	*NM_144665*	*SESN3*
1566363_at	*1.82*	*2.04*	*deoxynucleotidyltransferase, terminal*	*NM_001017520 /// NM_004088*	*DNTT*
216813_at	*1.81*	*1.76*			
206579_at	*1.80*	*2.04*	*zinc finger protein 192*	*NM_006298*	*ZNF192*
202086_at	*1.80*	*5.88*	*myxovirus (influenza virus) resistance 1, interferon-inducible protein p78 (mouse)*	*NM_002462*	*MX1*
1557120_at	*1.80*	*7.21*	*eukaryotic translation elongation factor 1 alpha 1*	*NM_001402*	*EEF1A1*
206488_s_at	*1.78*	*2.41*	*CD36 molecule (thrombospondin receptor)*	*NM_000072 /// NM_001001547 /// NM_001001548*	*CD36*
1562103_at	*1.78*	*2.36*	*Janus kinase 1 (a protein tyrosine kinase)*	*NM_002227*	*JAK1*

1568876_a_at	1.77	3.68			
242938_s_at	1.77	1.79	forkhead box K2	NM_004514	FOXK2
204735_at	1.76	2.07	phosphodiesterase 4A, cAMP-specific (phosphodiesterase E2 dunce homolog, Drosophila)	NM_006202	PDE4A
244150_at	1.76	2.68	structural maintenance of chromosomes 1A	NM_006306	SMC1A
215071_s_at	1.76	2.42	histone cluster 1, H2ac	NM_003512	HIST1H2AC
1561181_at	1.75	2.18	AT rich interactive domain 5B (MRF1-like)	NM_032199	ARID5B
1554319_at	1.75	3.43	ribosomal protein S6 kinase, 90kDa, polypeptide 5	NM_004755 /// NM_182398	RPS6KA5
237009_at	1.75	1.78	CD69 molecule	NM_001781	CD69
237891_at	1.75	1.96	Mdm2 p53 binding protein homolog (mouse)	NM_002392 /// NM_006878 /// NM_006879 /// NM_006881 /// NM_006882	MDM2
222802_at	1.74	4.68	endothelin 1	NM_001955	EDN1
243724_at	-1.75	-2.17			
219788_at	-1.75	-1.77	paired immunoglobin-like type 2 receptor alpha	NM_013439 /// NM_178272 /// NM_178273	PILRA
238607_at	-1.76	-11.64	zinc finger protein 342	NM_145288	ZNF342
204848_x_at	-1.76	-2.34	hemoglobin, gamma A	NM_000184 /// NM_000559	HBG1
1555205_at	-1.77	-2.00			
244190_at	-1.79	-1.77	THAP domain containing 5	NM_182529	THAP5
239602_at	-1.80	-1.93			
232760_at	-1.80	-1.864	testis expressed 15	NM_031271	TEX15
220843_s_at	-1.81	-2.57	WD repeats and SOF1 domain containing	NM_015420	WDSOF1
238726_at	-1.81	-1.97	ubiquitin specific peptidase 46	NM_022832	USP46
1569022_a_at	-1.82	-2.10	phosphoinositide-3-kinase, class 2, alpha polypeptide	NM_002645	PIK3C2A
205025_at	-1.82	-1.89	zinc finger and BTB domain containing 48	NM_005341	ZBTB48
235675_at	-1.82	-2.13	dihydrofolate reductase-like 1	NM_176815	DHFRL1
1557126_a_at	-1.83	-2.50	phospholipase D1, phosphatidylcholine-specific	NM_002662	PLD1
241104_at	-1.83	-2.16	sorbin and SH3 domain containing 2	NM_003603 /// NM_021069	SORBS2
1570528_at	-1.84	-2.96	xylosyltransferase II	NM_022167	XYLT2
223680_at	-1.84	-1.76		NM_032689 /// XM_927632 /// XM_940452	

1556783_a_at	-1.84	-1.93	chromosome 12 open reading frame 62	NM_032901	C12orf62
213515_x_at	-1.85	-2.43	hemoglobin, gamma A	NM_000184 /// NM_000559	HBG1
1559462_at	-1.85	-2.31			
214917_at	-1.86	-1.82	protein kinase, AMP-activated, alpha 1 catalytic subunit	NM_006251 /// NM_206907	PRKAA1
202490_at	-1.87	-1.87	inhibitor of kappa light polypeptide gene enhancer in B-cells, kinase complex-associated protein	NM_003640	IKBKAP
1564215_at	-1.87	-10.47	myosin XIX	NM_001033579 /// NM_001033580 /// NM_025109	MYO19
204120_s_at	-1.87	-1.98	adenosine kinase	NM_001123 /// NM_006721	ADK
211101_x_at	-1.89	-2.48	leukocyte immunoglobulin-like receptor, subfamily B (with TM and ITIM domains), member 1	NM_006866	LILRB1
221212_x_at	-1.90	-2.16	polybromo 1	NM_018165 /// NM_018313 /// NM_181042	PBRM1
1553644_at	-1.94	-3.34	chromosome 14 open reading frame 49	NM_152592	C14orf49
1557744_at	-1.95	-1.89			
216063_at	-1.97	-3.08	hemoglobin, beta pseudogene 1	NR_001589	HBBP1
1552910_at	-1.99	-3.13	sialic acid binding Ig-like lectin 11	NM_052884	SIGLEC11
217369_at	-2.01	-6.12	immunoglobulin heavy constant gamma 1 (G1m marker)	XM_370973	IGHG1
222881_at	-2.01	-1.90	heparanase	NM_006665	HPSE
211905_s_at	-2.02	-2.15	integrin, beta 4	NM_000213 /// NM_001005619 /// NM_001005731	ITGB4
244818_at	-2.03	-1.91			
238810_at	-2.03	-1.74	regulatory factor X, 3 (influences HLA class II expression)	NM_002919 /// NM_134428	RFX3
236510_at	-2.12	-1.93	HLA complex group 18	XM_933653 /// XM_936164 /// XM_944855	HCG18
204589_at	-2.15	-3.82	NUAK family, SNF1-like kinase, 1	NM_014840	NUAK1
1559528_at	-2.21	-1.79		XM_937486	

229157_at	*-2.31*	*-3.70*	*amiloride binding protein 1 (amine oxidase (copper-containing))*	*NM_001091*	*ABP1*
241060_x_at	*-2.31*	*-3.08*	*tetraspanin 5*	*NM_005723*	*TSPAN5*
1569566_at	*-2.32*	*-3.23*	*TBC1 (tre-2/USP6, BUB2, cdc16) domain family, member 1*	*NM_015173*	*TBC1D1*
217662_x_at	*-2.34*	*-2.31*	*B-cell receptor-associated protein 29*	*NM_001008405 /// NM_001008406 /// NM_001008407 /// NM_018844*	*BCAP29*
230768_at	*-2.35*	*-2.60*	*FERM, RhoGEF and pleckstrin domain protein 2*	*NM_014808*	*FARP2*
1556743_at	*-2.35*	*-1.96*			
232990_at	*-2.41*	*-1.86*	*family with sequence similarity 104, member B*	*NM_138362*	*FAM104B*
244193_at	*-2.43*	*-1.82*		*NM_024902*	
228772_at	*-2.51*	*-2.93*	*histamine N-methyltransferase*	*NM_001024074 /// NM_001024075 /// NM_006895*	*HNMT*
237835_at	*-2.66*	*-5.38*			
221176_x_at	*-2.75*	*-6.04*	*Williams-Beuren syndrome chromosome region 23*	*NM_001039145*	*WBSCR23*
231166_at	*-2.92*	*-2.34*	*G protein-coupled receptor 155*	*NM_001033045 /// NM_152529*	*GPR155*
1556697_at	*-2.93*	*-10.64*	*GPRIN family member 3*	*NM_198281*	*GPRIN3*
217670_at	*-2.97*	*-5.09*	*tetraspanin 4*	*NM_001025234 /// NM_001025235 /// NM_001025236 /// NM_001025237 /// NM_001025238 /// NM_001025239 /// NM_003271*	*TSPAN4*
213744_at	*-3.03*	*-2.08*	*attractin-like 1*	*NM_207303*	*ATRNL1*
216377_x_at	*-3.13*	*-2.74*	*alkaline phosphatase, placental-like 2*	*NM_031313*	*ALPPL2*
230535_s_at	*-3.35*	*-2.61*			
214377_s_at	*-3.54*	*-2.88*	*chymotrypsin-like*	*NM_001907*	*CTRL*
1560973_a_at	*-3.62*	*-10.32*			

220474_at	-3.75	-2.72	solute carrier family 25 (mitochondrial oxodicarboxylate carrier), member 21	NM_030631	SLC25A21
242868_at	-4.12	-4.22	endothelial PAS domain protein 1	NM_001430	EPAS1
229316_at	-4.17	-1.78	chromosome 19 open reading frame 50	NM_024069	C19orf50
235372_at	-4.63	-1.79	Fc receptor-like A	NM_032738	FCRLA
215868_x_at	-4.80	-12.05	SRY (sex determining region Y)-box 5	NM_006940 /// NM_152989 /// NM_178010	SOX5
235874_at	-4.93	-3.06	protease, serine, 35	NM_153362	PRSS35
217552_x_at	-5.39	-2.09	complement component (3b/4b) receptor 1 (Knops blood group)	NM_000573 /// NM_000651	CR1
207633_s_at	-5.44	-1.77	muscle, skeletal, receptor tyrosine kinase	NM_005592	MUSK
240609_at	-6.23	-2.49			
220931_at	-9.85	-4.19		XM_930060 /// XM_941452	
236805_at	-10.05	-5.49	chromosome 9 open reading frame 96	NM_153710	C9orf96
244767_at	-10.39	-12.77			
1557703_at	-10.65	-9.72	membrane-associated ring	NM_022826	7-Mar
220811_at	-10.82	-2.57	proteoglycan 3	NM_006093	PRG3
243855_at	-13.15	-2.73	small nuclear ribonucleoprotein polypeptide N	NM_003097 /// NM_022805 /// NM_022806 /// NM_022807 /// NM_022808	SNRPN
233051_at	-13.26	-4.75	SLIT and NTRK-like family, member 2	NM_032539	SLITRK2
1570006_at	-14.44	-2.22		XM_379100 /// XM_945436	
1555339_at	-15.58	-7.05	RAP1A, member of RAS oncogene family	NM_001010935 /// NM_002884	RAP1A
1566046_at	-19.53	-1.76	DEAH (Asp-Glu-Ala-His) box	NM_019030	DHX29
1555340_x_at	-21.53	-7.98	RAP1A, member of RAS oncogene family	NM_001010935 /// NM_002884	RAP1A
206647_at	-29.82	-3.78	hemoglobin, zeta	NM_005332	HBZ
