# Supplementary material for: Dissection of the Transformation of Primary Human Hematopoietic Cells by the Oncogene NUP98-HOXA9
Source: PLoS One. 2009 Aug 21;4(8):e6719. doi: 10.1371/journal.pone.0006719 (PMC2725295; doi:10.1371/journal.pone.0006719)
Supplement: Table S4 — Genes dysregulated by wild-type HOXA9. Primary human CD34+ cells were retrovirally transduced with either control MSCV-IRES-GFP vector or vector expressing wild-type HOXA9. Cells were sorted for GFP positivity and total RNA was subjected to microarray analysis. The experiment was performed two independent times and only genes that showed up- or down-regulation by 1.74 fold or more compared to control in both experiments were considered dysregulated. (0.06 MB PDF) [file pone.0006719.s004.pdf]

Table S4. Genes dysregulated by wild-type HOXA9

Probeset I.D.	Fold Change		Gene Name	Accession Number	Gene Symbol
	Exp.1	*Exp.2*			
1552323_s_at	*2.37*	*2.16*	*family with sequence similarity 122C*	*NM_002995 /// NM_003175*	*FAM122C*
1552480_s_at	*2.70*	*2.13*	*protein tyrosine phosphatase, receptor*	*NM_001753*	*PTPRC*
1552497_a_at	*2.96*	*3.03*	*SLAM family member 6*	*NM_003175*	*SLAMF6*
1552732_at	*4.38*	*1.92*	*actin-binding Rho activating protein*		*ABRA*
1552783_at	*2.35*	*1.84*	*zinc finger protein 417*	*NM_030801 /// NM_177535 /// NM_177537 /// XM_927162 /// XM_931877 /// XM_931884 /// XM_931888 /// XM_931897 /// XM_931904 /// XM_931909 /// XM_931916 /// XM_931919 /// XM_931924 /// XM_931929 /// XM_931936 /// XM_931943*	*ZNF417*
1553453_at	*1.94*	*2.95*	*ankyrin repeat and*	*NM_002523*	*ASB14*
1553635_s_at	*2.48*	*3.14*	*Tctex1 domain containing 1*	*NM_000550*	*TCTEX1D1*
1553645_at	*20.50*	*7.29*	*coiled-coil domain containing 141*	*NM_001649*	*CCDC141*
1553705_a_at	*1.98*	*2.22*		*NM_032866*	
1554205_s_at	*2.57*	*2.57*	*islet cell autoantigen 1,69kDa-like*		*ICA1L*
1554549_a_at	*2.28*	*1.81*	*WD repeat domain 20*	*NM_001017372 /// NM_014031*	*WDR20*
1554564_a_at	*1.98*	*1.94*			
1554606_at	*2.00*	*2.68*	*coiled-coil domain containing 100*	*NM_018327*	*CCDC100*
1555122_at	*3.52*	*3.07*	*G protein-coupled receptor 125*	*NM_001753*	*GPR125*
1555323_at	*-1.79*	*-2.02*	*ATP-binding cassette, sub-family B (MDR/TAP), member 9*	*NM_024336*	*ABCB9*
1555474_at	*1.94*	*2.01*	*tubulin tyrosine ligase-*		*TTLL3*
1555536_at	*2.27*	*2.88*	*anthrax toxin receptor 2*	*NM_153235*	*ANTXR2*
1555681_at	*2.12*	*1.79*		*NM_002126*	

1555728_a_at	*1.79*	*2.73*	*membrane-spanning 4-domains, subfamily A, member 4*	*NM_173648*	*MS4A4A*
1555775_a_at	*-1.96*	*-2.42*	*zygote arrest 1*	*NM_001031692 /// NM_005824*	*ZAR1*
1555897_at	*2.90*	*2.41*	*amine oxidase (flavin containing) domain 2*	*NM_031455*	*AOF2*
1555932_at	*3.18*	*6.99*	*testis specific, 10*		*TSGA10*
1556122_at	*1.92*	*1.76*		*NM_001032409 /// NM_002534 /// NM_016816*	
1556170_at	*-2.15*	*-2.28*	*mastermind-like 3 (Drosophila)*	*NM_001015055 /// NM_001015056 /// NM_033046*	*MAML3*
1556212_x_at	*2.03*	*2.46*		*NM_001040142 /// NM_001040143 /// NM_021007*	
1556462_a_at	*2.36*	*3.07*	*Kruppel-like factor 12*	*NM_147189*	*KLF12*
1556607_at	*-5.61*	*-3.11*	*EH-domain containing 4*		*EHD4*
1556698_a_at	*1.76*	*2.48*	*GPRIN family member 3*	*NM_001037339 /// NM_001037340 /// NM_001037341 /// NM_002600*	*GPRIN3*
1556898_at	*2.56*	*1.97*		*XM_497731*	
1556905_at	*2.18*	*2.49*	*zinc finger protein 577*	*NM_013230 /// XM_942808 /// XM_942813 /// XM_942817*	*ZNF577*
1557113_at	*2.66*	*2.46*		*NM_002546*	
1557126_a_at	*-2.00*	*-2.02*	*phospholipase D1, phosphatidylcholine-specific*	*NM_001033053 /// NM_014922 /// NM_033004 /// NM_033006 /// NM_033007*	*PLD1*
1557366_at	*-2.01*	*-2.48*	*coiled-coil domain containing 144B*	*NM_013230*	*CCDC144B*
1557386_at	*1.90*	*7.21*	*lactase*	*NM_022467*	*LCT*
1557664_at	*2.50*	*2.47*		*NM_014677*	
1557673_at	*2.65*	*2.22*	*leucine rich repeat containing 16A*		*LRRC16A*
1557756_a_at	*1.94*	*1.77*	*chromosome 14 open reading frame 145*	*NM_031909*	*C14orf145*
1558371_a_at	*-2.14*	*-2.25*	*glutamate-rich 1*	*NM_002522*	*ERICH1*
1558754_at	*2.22*	*2.36*	*zinc finger protein 763*	*NM_013230*	*ZNF763*
1559580_at	*2.35*	*3.38*	*leucine rich repeat containing 39*	*NM_020642 /// NM_030952 /// NM_182901*	*LRRC39*
1559633_a_at	*1.83*	*2.65*	*cholinergic receptor, muscarinic 3*	*NM_006045*	*CHRM3*

1559867_at	2.01	2.13		NM_001723 /// NM_015548 /// NM_020388 /// NM_183380	
1559975_at	2.12	1.84	B-cell translocation	NM_025113	BTG1
1560075_at	-5.73	-1.79	zinc finger protein 622	NM_007128	ZNF622
1560169_at	2.75	9.84	phosphatidylinositol-4-phosphate 5-kinase, type I, beta	NM_018357 /// NM_197958	PIP5K1B
1561181_at	3.98	3.58	AT rich interactive domain 5B (MRF1-like)	NM_001882	ARID5B
1561292_at	3.85	1.97		NM_017970 /// NM_199043	
1561310_at	2.05	3.21		NM_013230	
1561340_at	-86.00	-16.23		NM_013230	
1562386_s_at	2.89	1.75	zinc finger protein 501	XM_166132 /// XM_929312 /// XM_933582 /// XM_933586 /// XM_940811 /// XM_944813 /// XM_944815 /// XM_944819	ZNF501
1562403_a_at	2.17	2.25	solute carrier family 8 (sodium-calcium exchanger), member 3	NM_005503	SLC8A3
1562546_at	13.50	1.87		NM_025113	
1562904_s_at	-2.78	-2.18			
1563445_x_at	-2.66	-3.06	cathepsin L-like 3	NM_014988	CTSLL3
1563469_at	2.58	2.65	AT rich interactive domain 5B (MRF1-like)	NM_199339	ARID5B
1563495_at	1.85	3.03	solute carrier family 9, member 11	NM_003667	SLC9A11
1563646_a_at	1.83	1.76	transmembrane protein	NM_007173	TMEM67
1563833_at	3.93	2.34		NM_001147	
1564077_at	2.37	3.00	GPRIN family member 3	NM_000689	GPRIN3
1565016_at	1.88	2.01	protein arginine methyltransferase 1	NM_006614	PRMT1
1565804_at	2.06	1.93	ataxin 1	NM_006820	ATXN1
1565898_at	-11.28	-2.05	methyltransferase 5 domain containing 1	NM_020808	METT5D1
1566557_at	1.87	2.12		NM_001001887	
1566852_at	3.02	11.13	tripartite motif-containing 42	NM_015595	TRIM42
1566966_at	1.90	1.91	dihydropyrimidine dehydrogenase		DPYD
1567008_at	-5.27	-2.35		NM_000921	
1568646_x_at	2.04	2.75	zinc finger protein 208	NM_002977	ZNF208
1568807_a_at	1.79	2.41		NM_013431	
1568892_at	2.12	2.00		NM_173808	
1569003_at	1.82	2.56	transmembrane protein	NM_018328	TMEM49

1569136_at	*2.25*	*2.30*	*mannosyl (alpha-1,3-)-glycoprotein beta-1,4-N-*	*NM_015032*	*MGAT4A*
1569208_a_at	*6.92*	*2.22*	*LIM and calponin homology domains 1*		*LIMCH1*
1569318_at	*-6.69*	*-1.93*		*NM_001461*	
1569542_at	*2.99*	*2.04*	*G protein-coupled receptor 125*	*NM_002164*	*GPR125*
1569688_at	*2.74*	*3.37*	*flavin containing monooxygenase 5*	*NM_003118*	*FMO5*
1569690_at	*1.75*	*2.28*	*coiled-coil domain containing 36*	*NM_014682*	*CCDC36*
1569934_at	*1.99*	*1.88*	*DEP domain containing*	*NM_138554*	*DEPDC2*
1570021_at	*2.40*	*1.88*		*NM_020808*	
1570111_at	*1.88*	*5.83*	*chromosome 14 open reading frame 48*	*NM_001197*	*C14orf48*
1570119_at	*5.51*	*1.75*	*PDS5, regulator of cohesion maintenance, homolog B (S. cerevisiae)*	*NM_001001924 /// NM_001001925 /// NM_001001927 /// NM_001001931 /// NM_020749*	*PDS5B*
1570375_at	*1.95*	*1.80*	*protein tyrosine phosphatase, receptor*	*XM_927616*	*PTPRN2*
1570505_at	*2.66*	*2.39*	*ATP-binding cassette, sub-family B (MDR/TAP), member 4*	*NM_003107*	*ABCB4*
1570621_at	*2.05*	*2.16*	*AF4/FMR2 family, member 1*	*NM_031282*	*AFF1*
1570623_at	*4.57*	*3.31*		*NM_002483*	
200923_at	*2.44*	*2.93*	*lectin, galactoside-binding, soluble, 3*	*NM_031282*	*LGALS3BP*
201034_at	*1.91*	*2.12*	*adducin 3 (gamma)*	*NM_005503*	*ADD3*
201147_s_at	*-1.80*	*-2.86*	*TIMP metallopeptidase inhibitor 3 (Sorsby fundus dystrophy, pseudoinflammatory)*	*NM_002228*	*TIMP3*
201242_s_at	*1.90*	*1.82*	*ATPase, Na+/K+ transporting, beta 1 polypeptide*		*ATP1B1*
201324_at	*1.84*	*6.78*	*epithelial membrane*	*NM_004210*	*EMP1*
201325_s_at	*1.83*	*6.21*	*epithelial membrane*	*NM_012342*	*EMP1*
201416_at	*4.89*	*3.14*	*SRY (sex determining region Y)-box 4*		*SOX4*
201417_at	*2.04*	*2.87*	*SRY (sex determining region Y)-box 4*		*SOX4*
201418_s_at	*2.68*	*2.61*	*SRY (sex determining region Y)-box 4*	*NM_139166*	*SOX4*
201427_s_at	*1.91*	*2.49*	*selenoprotein P, plasma, 1*	*NM_012417 /// NM_181671*	*SEPP1*
201464_x_at	*2.79*	*4.27*	*jun oncogene*		*JUN*
201466_s_at	*4.60*	*3.67*	*jun oncogene*	*NM_000204*	*JUN*

201601_x_at	*2.32*	*2.82*	*interferon induced transmembrane protein 2 (1-8D)*	*NM_173078*	*IFITM2*
201752_s_at	*1.75*	*1.80*	*adducin 3 (gamma)*	*NM_006144*	*ADD3*
201753_s_at	*1.85*	*2.33*	*adducin 3 (gamma)*	*NM_021202*	*ADD3*
201888_s_at	*1.80*	*2.79*	*interleukin 13 receptor, alpha 1*	*NM_033502*	*IL13RA1*
201939_at	*2.44*	*2.00*	*polo-like kinase 2 (Drosophila)*	*NM_000199*	*PLK2*
202086_at	*3.42*	*6.81*	*myxovirus (influenza virus) resistance 1, interferon-inducible*	*NM_025074*	*MX1*
202254_at	*2.79*	*3.36*	*signal-induced proliferation-associated*	*NM_002077*	*SIPA1L1*
202350_s_at	*3.35*	*4.24*	*matrilin 2*	*XM_929318 /// XM_939083*	*MATN2*
202464_s_at	*1.78*	*1.91*	*6-phosphofructo-2-kinase/fructose-2,6-biphosphatase 3*	*NM_017565*	*PFKFB3*
202769_at	*1.83*	*2.01*	*cyclin G2*	*NM_005771 /// NM_199204*	*CCNG2*
202980_s_at	*2.13*	*2.39*	*seven in absentia homolog 1 (Drosophila)*	*NM_032199*	*SIAH1*
203060_s_at	*1.79*	*1.83*	*3'-phosphoadenosine 5'-phosphosulfate*	*NM_001002295 /// NM_002051*	*PAPSS2*
203065_s_at	*22.32*	*21.77*	*caveolin 1, caveolae protein, 22kDa*		*CAV1*
203153_at	*6.27*	*15.42*	*interferon-induced protein with*	*NM_003512*	*IFIT1*
203217_s_at	*1.80*	*2.07*	*ST3 beta-galactoside alpha-2,3-sialyltransferase 5*	*NM_000933 /// NM_182797*	*ST3GAL5*
203232_s_at	*2.32*	*2.19*	*ataxin 1*	*NM_020469*	*ATXN1*
203304_at	*4.54*	*4.05*	*BMP and activin membrane-bound inhibitor homolog*	*NM_000933 /// NM_182797*	*BAMBI*
203417_at	*1.88*	*29.18*	*microfibrillar-associated protein 2*		*MFAP2*
203476_at	*2.11*	*4.83*	*trophoblast glycoprotein*	*NM_032772*	*TPBG*
203485_at	*2.53*	*2.32*	*reticulon 1*		*RTN1*
203657_s_at	*1.77*	*1.78*	*cathepsin F*	*NM_002483*	*CTSF*
203708_at	*1.94*	*2.49*	*phosphodiesterase 4B, cAMP-specific (phosphodiesterase E4 dunce homolog, Drosophila)*	*NM_001007022 /// NM_020729*	*PDE4B*
203717_at	*-1.76*	*-2.43*	*dipeptidyl-peptidase 4 (CD26, adenosine deaminase complexing*	*NM_002126*	*DPP4*

203735_x_at	2.16	1.84	PTPRF interacting protein, binding protein 1 (liprin beta 1)	NM_030756	PPFIBP1
203739_at	1.84	1.87	zinc finger protein 217	NM_001039183	ZNF217
203757_s_at	3.76	3.38	carcinoembryonic antigen-related cell adhesion molecule 5	NM_001839	CEACAM5
203821_at	2.51	3.10	heparin-binding EGF-like growth factor		HBEGF
203854_at	4.31	6.87	complement factor I	NM_003326	CFI
203895_at	3.87	3.32	phospholipase C, beta 4		PLCB4
203896_s_at	3.90	4.48	phospholipase C, beta 4		PLCB4
203910_at	-36.23	-6.36	Rho GTPase activating protein 29	NM_004120	ARHGAP29
204001_at	1.77	1.87	small nuclear RNA activating complex, polypeptide 3, 50kDa	NM_145290	SNAPC3
204018_x_at	-1.79	-7.70	hemoglobin, alpha 1	NM_002126	HBA1
204070_at	1.94	1.84	retinoic acid receptor responder (tazarotene induced) 3		RARRES3
204201_s_at	1.93	2.03	protein tyrosine phosphatase, non-receptor type 13 (APO-1/CD95 (Fas)-associated		PTPN13
204205_at	2.58	2.63	apolipoprotein B mRNA editing enzyme, catalytic polypeptide-like	NM_203459	APOBEC3G
204217_s_at	2.05	2.76	reticulon 2	NM_006343	RTN2
204221_x_at	3.21	3.53	GLI pathogenesis-related 1 (glioma)		GLIPR1
204271_s_at	1.91	2.95	endothelin receptor type	NM_003866	EDNRB
204304_s_at	1.99	2.05	prominin 1	NM_002462	PROM1
204415_at	2.82	3.14	interferon, alpha-inducible protein 6	NM_001002264 /// NM_033255	IFI6
204419_x_at	-2.31	-6.00	hemoglobin, gamma A	NM_013230	HBG1
204439_at	6.40	11.88	interferon-induced protein 44-like	NM_015976 /// NM_152238	IFI44L
204455_at	9.31	8.24	dystonin	NM_002380 /// NM_030583	DST
204567_s_at	2.12	1.88	ATP-binding cassette, sub-family G (WHITE),		ABCG1
204591_at	6.40	21.57	cell adhesion molecule with homology to L1CAM (close homolog	NM_002718 /// NM_181897	CHL1
204633_s_at	1.84	2.03	ribosomal protein S6 kinase, 90kDa, polypeptide 5		RPS6KA5

204635_at	*1.84*	*2.35*	*ribosomal protein S6 kinase, 90kDa, polypeptide 5*	*NM_032738*	*RPS6KA5*
204684_at	*9.92*	*2.33*	*neuronal pentraxin I*	*NM_006851*	*NPTX1*
204747_at	*2.96*	*2.44*	*interferon-induced protein with*	*NM_000499*	*IFIT3*
204753_s_at	*3.72*	*3.76*	*hepatic leukemia factor*	*NM_001033569 /// NM_001033570 /// NM_001033571 /// NM_001033572 /// NM_001033574 /// NM_016627*	*HLF*
204754_at	*21.30*	*4.48*	*hepatic leukemia factor*	*NM_025244 /// NM_182911*	*HLF*
204755_x_at	*3.51*	*3.61*	*hepatic leukemia factor*		*HLF*
204848_x_at	*-1.98*	*-5.38*	*hemoglobin, gamma A*	*NM_000933 /// NM_182797*	*HBG1*
204889_s_at	*4.57*	*3.17*	*neuralized homolog (Drosophila)*	*NM_032199*	*NEURL*
204932_at	*13.06*	*1.85*	*tumor necrosis factor receptor superfamily, member 11b*	*NM_153046*	*TNFRSF11B*

204967_at	*29.64*	*2.30*	*shroom family member 2*	*XM_496237 /// XM_930513 /// XM_935185 /// XM_935186 /// XM_935187 /// XM_935188 /// XM_935189 /// XM_935190 /// XM_935191 /// XM_935192 /// XM_935193 /// XM_935194 /// XM_935195 /// XM_935196 /// XM_935197 /// XM_935198 /// XM_935200 /// XM_935201 /// XM_940040 /// XM_942463 /// XM_945997 /// XM_945998 /// XM_945999 /// XM_946000 /// XM_946001 /// XM_946002 /// XM_946003 /// XM_946004 /// XM_946005 /// XM_946007 /// XM_946008 /// XM_946009 ///*	*SHROOM2*
204972_at	*2.45*	*3.34*	*2'-5'-oligoadenylate synthetase 2, 69/71kDa*	*NM_000856*	*OAS2*
205016_at	*-2.08*	*-1.98*	*transforming growth factor, alpha*	*NM_005814*	*TGFA*
205159_at	*-2.08*	*-2.72*	*colony stimulating factor 2 receptor, beta, low-affinity (granulocyte-*	*NM_174926*	*CSF2RB*
205376_at	*3.44*	*3.83*	*inositol polyphosphate-4-phosphatase, type II, 105kDa*	*NM_019012*	*INPP4B*
205381_at	*18.11*	*5.87*	*leucine rich repeat containing 17*	*NM_152616*	*LRRC17*
205383_s_at	*2.00*	*1.94*	*zinc finger and BTB domain containing 20*	*NM_005771 /// NM_199204*	*ZBTB20*
205390_s_at	*-14.26*	*-2.17*	*ankyrin 1, erythrocytic*	*NM_000903 /// NM_001025433 /// NM_001025434*	*ANK1*

205466_s_at	*1.88*	*1.98*	*heparan sulfate (glucosamine) 3-O-sulfotransferase 1*	*NM_005771 /// NM_199204*	*HS3ST1*
205483_s_at	*1.87*	*2.36*	*ISG15 ubiquitin-like modifier*		*ISG15*
205488_at	*4.23*	*3.97*	*granzyme A (granzyme 1, cytotoxic T-lymphocyte-associated serine esterase 3)*	*NM_004318 /// NM_020164 /// NM_032466 /// NM_032467 /// NM_032468*	*GZMA*
205513_at	*1.74*	*2.11*	*transcobalamin I (vitamin B12 binding protein, R binder family)*	*NM_145290*	*TCN1*
205552_s_at	*16.89*	*26.56*	*2',5'-oligoadenylate synthetase 1, 40/46kDa*	*NM_018165 /// NM_018313 /// NM_181042*	*OAS1*
205572_at	*6.69*	*21.32*	*angiopoietin 2*	*NM_001031683 /// NM_001549*	*ANGPT2*
205573_s_at	*3.36*	*3.87*	*sorting nexin 7*	*NM_052931*	*SNX7*
205609_at	*2.47*	*1.79*	*angiopoietin 1*	*NM_003196*	*ANGPT1*
205624_at	*-2.92*	*-2.22*	*carboxypeptidase A3 (mast cell)*	*NM_015013*	*CPA3*
205626_s_at	*2.68*	*3.04*	*calbindin 1, 28kDa*	*NM_032495 /// NM_139211 /// NM_139212*	*CALB1*
205653_at	*2.13*	*1.94*	*cathepsin G*	*NM_145044*	*CTSG*
205694_at	*30.54*	*21.46*	*tyrosinase-related*	*NM_003107*	*TYRP1*
205749_at	*3.19*	*5.52*	*cytochrome P450, family 1, subfamily A, polypeptide 1*	*NM_001547*	*CYP1A1*
205780_at	*5.12*	*12.58*	*BCL2-interacting killer (apoptosis-inducing)*	*XM_376281 /// XM_941354*	*BIK*
205882_x_at	*1.81*	*1.93*	*adducin 3 (gamma)*	*NM_032199*	*ADD3*
205929_at	*3.07*	*4.66*	*glycoprotein A33 (transmembrane)*	*NM_001001132 /// NM_003024*	*GPA33*
205967_at	*-1.77*	*-1.82*	*histone cluster 1, H4c*	*NM_152588*	*HIST1H4C*
205984_at	*8.66*	*3.63*	*corticotropin releasing hormone binding protein*	*NM_017644*	*CRHBP*
205991_s_at	*-2.36*	*-2.84*	*paired related*	*NM_024614*	*PRRX1*
206009_at	*2.31*	*3.93*	*integrin, alpha 9*	*NM_002038 /// NM_022872 /// NM_022873*	*ITGA9*
206135_at	*5.23*	*33.43*	*suppression of tumorigenicity 18 (breast carcinoma) (zinc*	*NM_002228*	*ST18*
206207_at	*-2.20*	*-1.90*	*Charcot-Leyden crystal protein*	*NM_015556*	*CLC*
206310_at	*2.45*	*2.24*	*serine peptidase inhibitor, Kazal type 2 (acrosin-trypsin*	*NM_030756*	*SPINK2*

206318_at	*2.76*	*3.46*	*serine peptidase inhibitor-like, with Kunitz and WAP domains 1*	*NM_020398 /// NM_181502*	*SPINLW1*
206366_x_at	*79.77*	*25.07*	*chemokine (C motif) ligand 2*	*NM_001040214*	*XCL2*
206385_s_at	*2.52*	*3.52*	*ankyrin 3, node of Ranvier (ankyrin G)*	*NM_001031687 /// NM_003558*	*ANK3*
206390_x_at	*-2.14*	*-4.00*	*platelet factor 4 (chemokine (C-X-C motif) ligand 4)*	*NM_001461*	*PF4*
206548_at	*3.01*	*2.09*		*NM_031435*	
206579_at	*1.78*	*1.90*	*zinc finger protein 192*		*ZNF192*
206618_at	*-2.66*	*-1.96*	*interleukin 18 receptor 1*	*NM_032427*	*IL18R1*
206647_at	*-1.99*	*-12.99*	*hemoglobin, zeta*	*NM_013313*	*HBZ*
206707_x_at	*1.79*	*1.79*	*chromosome 6 open reading frame 32*	*NM_000452*	*C6orf32*
206828_at	*2.45*	*1.78*	*TXK tyrosine kinase*	*NM_001001132 /// NM_003024*	*TXK*
206834_at	*-2.03*	*-3.79*	*hemoglobin, delta*	*NM_014600*	*HBD*
206932_at	*-2.28*	*-2.24*	*cholesterol 25-hydroxylase*	*NM_002838 /// NM_080921 /// NM_080922 /// NM_080923*	*CH25H*
206950_at	*5.96*	*5.89*	*sodium channel, voltage-gated, type IX, alpha subunit*	*NM_182758*	*SCN9A*
207067_s_at	*-2.56*	*-2.52*	*histidine decarboxylase*		*HDC*
207095_at	*2.72*	*2.89*	*solute carrier family 10 (sodium/bile acid cotransporter family), member 2*	*NM_003107*	*SLC10A2*
207111_at	*2.23*	*3.79*	*egf-like module containing, mucin-like, hormone receptor-like 1*	*NM_004929*	*EMR1*
207161_at	*21.87*	*13.11*	*KIAA0087*	*NM_001198 /// NM_182907*	*KIAA0087*
207284_s_at	*3.00*	*3.31*	*aspartate beta-*	*NM_000076*	*ASPH*
207303_at	*-3.75*	*-3.66*	*phosphodiesterase 1C, calmodulin-dependent 70kDa*		*PDE1C*
207408_at	*1.83*	*2.02*	*solute carrier family 22, member 14*	*NM_000443 /// NM_018849 /// NM_018850*	*SLC22A14*
207426_s_at	*3.59*	*3.25*	*tumor necrosis factor (ligand) superfamily, member 4 (tax-transcriptionally*	*NM_013313*	*TNFSF4*
207496_at	*2.61*	*1.84*	*membrane-spanning 4-domains, subfamily A, member 2 (Fc fragment of IgE, high affinity I,*	*NM_017640*	*MS4A2*

207764_s_at	*2.10*	*1.77*	*homeodomain interacting protein*	*NM_006417*	*HIPK3*
207777_s_at	*1.77*	*4.11*	*SP140 nuclear body protein*	*NM_001012755*	*SP140*
207793_s_at	*2.48*	*1.77*	*erythrocyte membrane protein band 4.1 (elliptocytosis 1, RH-linked)*	*NM_007118*	*EPB41*
207857_at	*2.01*	*3.44*	*leukocyte immunoglobulin-like receptor, subfamily B (with TM and ITIM*	*NM_000139*	*LILRB1*
208436_s_at	*2.37*	*2.35*	*interferon regulatory factor 7*	*XM_043739 /// XM_935434 /// XM_935435 /// XM_942428 /// XM_945966 /// XM_945967*	*IRF7*
208488_s_at	*-2.06*	*-1.83*	*complement component (3b/4b) receptor 1 (Knops blood group)*		*CR1*
208498_s_at	*1.93*	*1.77*	*amylase, alpha 1A (salivary)*		*AMY1A*
208544_at	*2.37*	*1.96*	*adrenergic, alpha-2B-, receptor*	*NM_005294*	*ADRA2B*
208650_s_at	*13.42*	*10.66*		*NM_032199*	
208651_x_at	*11.40*	*13.81*	*CD24 molecule*	*NM_021822*	*CD24*
208782_at	*1.79*	*2.34*	*follistatin-like 1*	*NM_138468 /// NM_178231*	*FSTL1*
208891_at	*2.50*	*2.50*	*dual specificity phosphatase 6*	*NM_000856*	*DUSP6*
208892_s_at	*2.50*	*2.18*	*dual specificity phosphatase 6*	*NM_006866*	*DUSP6*
209010_s_at	*2.62*	*10.58*	*triple functional domain*		*TRIO*
209047_at	*-1.82*	*-2.19*	*aquaporin 1 (Colton blood group)*		*AQP1*
209116_x_at	*-2.54*	*-5.24*	*hemoglobin, beta*		*HBB*
209118_s_at	*2.31*	*2.55*	*tubulin, alpha 1a*	*NM_032199*	*TUBA1A*
209147_s_at	*-2.38*	*-2.47*	*phosphatidic acid phosphatase type 2A*	*NM_021136 /// NM_206852 /// NM_206857*	*PPAP2A*
209160_at	*2.08*	*2.14*	*aldo-keto reductase family 1, member C3 (3-*	*NM_001149 /// NM_020987*	*AKR1C3*
209298_s_at	*2.85*	*3.12*	*intersectin 1 (SH3 domain protein)*	*NM_015493*	*ITSN1*
209348_s_at	*1.92*	*2.28*	*v-maf musculoaponeurotic fibrosarcoma oncogene*	*NM_001945*	*MAF*
209356_x_at	*2.38*	*2.08*	*EGF-containing fibulin-like extracellular matrix protein 2*	*NM_000076*	*EFEMP2*

209458_x_at	*-2.12*	*-7.47*	*hemoglobin, alpha 1*	*NM_002077*	*HBA1*
209459_s_at	*2.44*	*5.45*	*4-aminobutyrate aminotransferase*	*NM_017691*	*ABAT*
209514_s_at	*1.79*	*2.16*	*RAB27A, member RAS oncogene family*		*RAB27A*
209515_s_at	*1.82*	*2.21*	*RAB27A, member RAS oncogene family*	*NM_001946 /// NM_022652*	*RAB27A*
209555_s_at	*-1.76*	*-2.19*	*CD36 molecule (thrombospondin*	*NM_001946 /// NM_022652*	*CD36*
209604_s_at	*3.95*	*8.15*	*GATA binding protein 3*	*NM_033514 /// XM_290985 /// XM_496582 /// XM_934628 /// XM_934629 /// XM_934630 /// XM_934631 /// XM_934633 /// XM_934634*	*GATA3*
209632_at	*3.32*	*2.84*	*protein phosphatase 2 (formerly 2A), regulatory subunit B'', alpha*	*NM_004437 /// NM_203342 /// NM_203343*	*PPP2R3A*
209771_x_at	*7.84*	*10.63*	*CD24 molecule*	*NM_152665*	*CD24*
209772_s_at	*3.37*	*7.00*	*CD24 molecule*	*NM_006045*	*CD24*
209829_at	*1.97*	*1.95*	*chromosome 6 open reading frame 32*	*NM_018165 /// NM_018313 /// NM_181042*	*C6orf32*
209870_s_at	*4.68*	*4.66*	*amyloid beta (A4) precursor protein-binding, family A, member 2 (X11-like)*	*NM_001146*	*APBA2*
209871_s_at	*7.34*	*16.34*	*amyloid beta (A4) precursor protein-binding, family A, member 2 (X11-like)*	*NM_002242*	*APBA2*
209994_s_at	*1.76*	*1.98*	*ATP-binding cassette, sub-family B (MDR/TAP), member 1*		*ABCB1*
210029_at	*5.36*	*2.32*	*indoleamine-pyrrole 2,3 dioxygenase*	*NM_080672 /// NM_183244 /// NM_183246*	*INDO*
210036_s_at	*-1.89*	*-2.41*	*potassium voltage-gated channel, subfamily H (eag-*	*NM_024989*	*KCNH2*
210179_at	*2.47*	*5.94*	*potassium inwardly-rectifying channel, subfamily J, member 13*	*NM_022824*	*KCNJ13*
210279_at	*2.04*	*2.54*	*G protein-coupled receptor 18*	*NM_017523 /// NM_199139*	*GPR18*
210414_at	*2.24*	*10.26*	*fibronectin leucine rich transmembrane protein*	*NM_003328*	*FLRT1*

210504_at	*-1.87*	*-2.72*	*Kruppel-like factor 1 (erythroid)*	*NM_001032731 /// NM_002535 /// NM_016817*	*KLF1*
210517_s_at	*-2.50*	*-1.96*	*A kinase (PRKA) anchor protein (gravin) 12*	*NM_021114*	*AKAP12*
210803_at	*2.15*	*4.02*	*thioredoxin reductase 2*	*NM_000663 /// NM_020686*	*TXNRD2*
210946_at	*-2.69*	*-1.98*	*phosphatidic acid phosphatase type 2A*	*NM_005567*	*PPAP2A*
210951_x_at	*2.15*	*2.40*	*RAB27A, member RAS oncogene family*	*NM_006622*	*RAB27A*
210954_s_at	*-2.41*	*-7.08*	*TSC22 domain family, member 2*	*NM_014602*	*TSC22D2*
211066_x_at	*1.80*	*2.10*	*protocadherin gamma subfamily A, 12*	*NM_173690*	*PCDHGA12*
211100_x_at	*1.79*	*2.95*	*leukocyte immunoglobulin-like receptor, subfamily B (with TM and ITIM*	*NM_002207*	*LILRB1*
211102_s_at	*2.57*	*3.43*	*leukocyte immunoglobulin-like receptor, subfamily B (with TM and ITIM*		*LILRB1*
211298_s_at	*-1.98*	*-4.95*	*albumin*	*NM_020397 /// NM_153498*	*ALB*
211597_s_at	*2.90*	*3.00*	*HOP homeobox*	*NM_015263*	*HOPX*
211657_at	*4.73*	*4.21*	*carcinoembryonic antigen-related cell adhesion molecule 5*	*NM_016938*	*CEACAM5*
211696_x_at	*-2.44*	*-4.16*	*hemoglobin, beta*	*NM_003369*	*HBB*
211699_x_at	*-2.09*	*-6.70*	*hemoglobin, alpha 1*	*XM_379398 /// XM_932815 /// XM_932820 /// XM_944201 /// XM_944203 /// XM_944207*	*HBA1*
211745_x_at	*-2.30*	*-7.51*	*hemoglobin, alpha 1*	*NM_198281*	*HBA1*
211883_x_at	*1.98*	*2.05*	*carcinoembryonic antigen-related cell adhesion molecule 1 (biliary glycoprotein)*	*NM_016205*	*CEACAM1*
212062_at	*9.43*	*11.83*	*ATPase, class II, type*	*NM_138819*	*ATP9A*
212096_s_at	*5.05*	*3.79*	*mitochondrial tumor suppressor 1*	*NM_000682*	*MTUS1*
212097_at	*99.14*	*31.15*	*caveolin 1, caveolae protein, 22kDa*		*CAV1*
212224_at	*6.56*	*8.09*	*aldehyde dehydrogenase 1 family, member A1*	*NM_001572 /// NM_004029 /// NM_004031*	*ALDH1A1*
212614_at	*2.87*	*3.09*	*AT rich interactive domain 5B (MRF1-like)*		*ARID5B*

212667_at	*5.24*	*1.90*	*secreted protein, acidic, cysteine-rich*		*SPARC*
212761_at	*1.99*	*2.64*	*transcription factor 7-like 2 (T-cell specific,*	*NM_007249 /// NM_016285*	*TCF7L2*
212765_at	*3.46*	*3.18*	*calmodulin regulated spectrin-associated protein 1-like 1*	*NM_144620*	*CAMSAP1L1*
212820_at	*2.38*	*3.49*	*Dmx-like 2*	*NM_000856*	*DMXL2*
213032_at	*-3.35*	*-2.00*	*nuclear factor I/B*	*NM_152475*	*NFIB*
213058_at	*2.05*	*1.90*	*tetratricopeptide repeat domain 28*	*NM_001001132 /// NM_003024*	*TTC28*
213082_s_at	*1.88*	*2.40*	*solute carrier family 35, member D2*	*NM_001037501 /// XM_496391 /// XM_929808 /// XM_933968 /// XM_933971 /// XM_933974 /// XM_933975 /// XM_933976 /// XM_933977 /// XM_933978 /// XM_933980 /// XM_933982 /// XM_933984 /// XM_933989 /// XM_933993 /// XM_933996 /// XM_934000 /// XM_934003 /// XM_934004 /// XM_934007 /// XM_934010 /// XM_934014 /// XM_934016 /// XM_934017 /// XM_934019 /// XM_934021 /// XM_934022 /// XM_934023 /// XM_934025 /// XM_934026 /// XM_934028 /// XM_934029 ///*	*SLC35D2*
213094_at	*1.95*	*2.09*	*G protein-coupled receptor 126*	*NM_003107*	*GPR126*
213135_at	*1.76*	*2.58*	*T-cell lymphoma invasion and metastasis*	*NM_000332*	*TIAM1*
213156_at	*1.80*	*1.95*		*NM_003641*	
213158_at	*2.16*	*2.11*		*NM_006009*	
213174_at	*-2.12*	*-4.76*	*tetratricopeptide repeat domain 9*	*NM_002207*	*TTC9*

213283_s_at	*-1.74*	*-1.85*	*sal-like 2 (Drosophila)*	*XM_496826 /// XM_933674 /// XM_933675 /// XM_933677 /// XM_941807 /// XM_945447 /// XM_945448 /// XM_945450*	*SALL2*
213316_at	*7.54*	*9.04*	*KIAA1462*	*NM_001037339 /// NM_001037340 /// NM_001037341 /// NM_002600*	*KIAA1462*
213348_at	*2.51*	*2.46*	*cyclin-dependent kinase inhibitor 1C (p57, Kip2)*	*NM_020654*	*CDKN1C*
213479_at	*34.38*	*10.36*	*neuronal pentraxin II*	*NM_001024948 /// NM_017737*	*NPTX2*
213515_x_at	*-2.35*	*-4.72*	*hemoglobin, gamma A*	*NM_080657*	*HBG1*
213541_s_at	*1.82*	*1.90*	*v-ets erythroblastosis virus E26 oncogene homolog (avian)*	*NM_144574 /// NM_181291 /// NM_181302 /// NM_181308*	*ERG*
213665_at	*2.89*	*2.65*	*SRY (sex determining region Y)-box 4*	*XM_371082 /// XM_930250 /// XM_934509 /// XM_934511 /// XM_934512 /// XM_934513 /// XM_934515 /// XM_934518 /// XM_934521 /// XM_934522 /// XM_942690 /// XM_946231 /// XM_946232 /// XM_946233 /// XM_946234 /// XM_946235 /// XM_946236*	*SOX4*
213668_s_at	*2.33*	*2.92*	*SRY (sex determining region Y)-box 4*	*NM_058172*	*SOX4*
213689_x_at	*1.81*	*2.45*	*ribosomal protein L5*	*NM_005619 /// NM_206900 /// NM_206901 /// NM_206902*	*RPL5*
213880_at	*6.88*	*5.13*	*leucine-rich repeat-containing G protein-coupled receptor 5*	*NM_018423*	*LGR5*
213963_s_at	*-3.27*	*-2.58*	*Sin3A-associated protein, 30kDa*		*SAP30*
213996_at	*2.66*	*2.45*	*yippee-like 1*		*YPEL1*

214084_x_at	1.95	2.72		NM_012214	
214146_s_at	-1.87	-2.09	pro-platelet basic protein (chemokine (C-X-C motif) ligand 7)	NM_018371	PPBP
214366_s_at	-2.19	-2.33	arachidonate 5-lipoxygenase	NM_013280	ALOX5
214414_x_at	-2.73	-9.13	hemoglobin, alpha 2	NM_213602	HBA2
214453_s_at	2.65	2.07	interferon-induced protein 44	NM_032584	IFI44
214567_s_at	125.15	29.40	chemokine (C motif) ligand 1	NM_001974	XCL1
214596_at	4.52	5.35		NM_001012753	
214627_at	-5.05	-2.53	eosinophil peroxidase	NM_004285	EPX
214997_at	4.06	1.84	golgi autoantigen, golgin subfamily a, 1	NM_005902	GOLGA1
215017_s_at	2.29	1.99	formin binding protein 1-like	NM_152544	FNBP1L
215071_s_at	3.92	4.75	histone cluster 1, H2ac	NM_152588	HIST1H2AC
215124_at	-15.32	-2.15	zinc finger protein 550	NM_020428	ZNF550
215146_s_at	4.05	1.96	tetratricopeptide repeat domain 28	NM_173690	TTC28
215288_at	1.77	3.15	transient receptor potential cation channel, subfamily C, member 2 (pseudogene)	XM_927459 /// XM_935508 /// XM_938444	TRPC2
215300_s_at	5.42	3.36	flavin containing monooxygenase 5		FMO5
215368_at	1.92	2.06	nebulin	NM_001032278 /// NM_024302	NEB
215478_at	10.77	1.90	regulating synaptic membrane exocytosis 2	NM_015116	RIMS2
215585_at	-2.40	-1.89	KIAA0174	NM_005230	KIAA0174
215671_at	13.58	7.95	phosphodiesterase 4B, cAMP-specific (phosphodiesterase E4 dunce homolog, Drosophila)	NM_032679	PDE4B
215761_at	1.90	2.25	Dmx-like 2		DMXL2
215791_at	2.71	4.10	intersectin 1 (SH3 domain protein)		ITSN1
215856_at	2.24	1.82	sialic acid binding Ig-like lectin 15		SIGLEC15
216129_at	2.48	2.63	ATPase, class II, type 9A	NM_033262 /// NM_058240 /// NM_182932 /// NM_182936 /// NM_183002	ATP9A
216317_x_at	-2.70	-4.78	Rh blood group, CcEe antigens	NM_003622 /// NM_177444	RHCE
216379_x_at	8.38	9.90	CD24 molecule		CD24
216511_s_at	3.72	1.82	transcription factor 7-like 2 (T-cell specific,	NM_006440	TCF7L2

216628_at	*3.30*	*3.52*		*NM_004580 /// NM_183234 /// NM_183235 /// NM_183236*	
216716_at	*3.89*	*3.54*	*ABO blood group (transferase A, alpha 1-3-N-acetylgalactosaminyltransferase; transferase B,*	*NM_005465 /// NM_181690*	*ABO*
216749_at	*4.11*	*5.79*	*transcriptional regulating factor 1*	*NM_002037 /// NM_153047 /// NM_153048*	*TRERF1*
216752_at	*2.43*	*5.30*	*phosphoinositide-3-kinase, regulatory subunit 4*	*NM_032379 /// NM_032943 /// NM_206927 /// NM_206928 /// NM_206929 /// NM_206930*	*PIK3R4*
216850_at	*1.94*	*2.99*	*small nuclear ribonucleoprotein polypeptide N*	*NM_001006610 /// NM_003031*	*SNRPN*
216894_x_at	*2.66*	*1.78*	*cyclin-dependent kinase inhibitor 1C (p57, Kip2)*	*NM_021197*	*CDKN1C*
217125_at	*1.86*	*2.72*		*NM_001911*	
217143_s_at	*-2.07*	*-2.13*	*T cell receptor alpha*		*TRA@*
217196_s_at	*2.11*	*2.17*	*calmodulin regulated spectrin-associated protein 1-like 1*	*NM_004753*	*CAMSAP1L1*
217203_at	*2.55*	*6.47*			
217232_x_at	*-2.09*	*-5.00*	*hemoglobin, beta*	*NM_004915 /// NM_016818 /// NM_207174 /// NM_207627 /// NM_207628 /// NM_207629 /// NM_207630*	*HBB*
217414_x_at	*-2.82*	*-9.06*	*hemoglobin, alpha 1*	*NM_001731*	*HBA1*
217540_at	*2.56*	*2.14*		*NM_030938*	
217549_at	*1.96*	*1.90*		*NM_031477*	
217552_x_at	*-8.35*	*-2.36*	*complement component (3b/4b) receptor 1 (Knops blood group)*	*NM_016644*	*CR1*
217787_s_at	*1.92*	*2.37*	*UDP-N-acetyl-alpha-D-galactosamine:polypeptide N-acetylgalactosaminyltransferase 2 (GalNAc-T2)*		*GALNT2*
217788_s_at	*2.01*	*1.76*	*UDP-N-acetyl-alpha-D-galactosamine:polypeptide N-acetylgalactosaminyltransferase 2 (GalNAc-T2)*	*NM_006670*	*GALNT2*

217867_x_at	*-1.76*	*-1.74*	*beta-site APP-cleaving enzyme 2*	*NM_203459*	*BACE2*
218284_at	*2.22*	*1.89*	*SMAD family member 3*	*NM_005734*	*SMAD3*
218309_at	*-3.00*	*-2.20*	*calcium/calmodulin-dependent protein kinase II inhibitor 1*	*NM_018699*	*CAMK2N1*
218380_at	*12.80*	*3.88*	*NLR family, pyrin domain containing 1*	*NM_022488*	*NLRP1*
218418_s_at	*2.52*	*2.94*	*KN motif and ankyrin repeat domains 2*	*NM_025189*	*KANK2*
218608_at	*1.75*	*2.10*	*ATPase type 13A2*	*NM_001945*	*ATP13A2*
218718_at	*2.37*	*2.59*	*platelet derived growth factor C*	*NM_003739*	*PDGFC*
218935_at	*2.70*	*1.95*	*EH-domain containing 3*		*EHD3*
219049_at	*2.24*	*1.92*	*chondroitin sulfate N-acetylgalactosaminyltransferase 1*		*CSGALNACT1*
219338_s_at	*2.50*	*3.25*	*leucine rich repeat containing 49*	*NM_024491*	*LRRC49*
219383_at	*1.89*	*2.89*		*NM_000332*	
219386_s_at	*-2.44*	*-1.84*	*SLAM family member 8*	*NM_001031679 /// NM_198080*	*SLAMF8*
219471_at	*9.04*	*36.04*	*chromosome 13 open reading frame 18*	*NM_005935*	*C13orf18*
219478_at	*2.13*	*3.56*	*WAP four-disulfide core domain 1*	*NM_005619 /// NM_206900 /// NM_206901 /// NM_206902*	*WFDC1*
219520_s_at	*2.01*	*2.06*	*WWC family member 3*		*WWC3*
219746_at	*-2.21*	*-1.87*	*D4, zinc and double PHD fingers, family 3*	*XM_933721 /// XM_935806 /// XM_943045*	*DPF3*
219799_s_at	*3.01*	*5.50*	*dehydrogenase/reductase (SDR family)*	*XM_929318 /// XM_939083*	*DHRS9*
219871_at	*2.82*	*2.11*		*NM_000964 /// NM_001024809 /// NM_001033603*	
219932_at	*24.98*	*14.23*	*solute carrier family 27 (fatty acid transporter), member 6*	*NM_003107*	*SLC27A6*
219944_at	*-1.85*	*-1.85*	*CAP-GLY domain containing linker protein family, member 4*	*NM_007153*	*CLIP4*
220014_at	*2.11*	*3.00*	*proline rich 16*	*NM_005292*	*PRR16*
220030_at	*1.82*	*4.89*	*serine/threonine/tyrosine kinase 1*	*NM_002214*	*STYK1*
220237_at	*2.08*	*1.84*	*ATG3 autophagy related 3 homolog (S.*	*XM_929541 /// XM_941868*	*ATG3*
220246_at	*2.39*	*1.89*	*calcium/calmodulin-dependent protein kinase ID*	*NM_001017523 /// NM_001018072 /// NM_152322*	*CAMK1D*

220576_at	*2.46*	*2.24*	*post-GPI attachment to proteins 1*	*NM_002841*	*PGAP1*
220593_s_at	*2.27*	*4.01*	*coiled-coil domain containing 40*	*NM_017554*	*CCDC40*
220726_at	*-18.76*	*-2.00*		*NM_001259*	
220735_s_at	*1.92*	*1.91*	*SUMO1/sentrin specific peptidase 7*	*NM_015691*	*SENP7*
220811_at	*-1.79*	*-2.00*	*proteoglycan 3*	*NM_015074 /// NM_183416*	*PRG3*
220891_at	*2.21*	*1.97*	*chromosome 4 open reading frame 23*	*NM_006866*	*C4orf23*
220952_s_at	*1.85*	*2.01*	*pleckstrin homology domain containing, family A member 5*	*NM_015691*	*PLEKHA5*
220987_s_at	*9.45*	*2.40*	*chromosome 11 open reading frame 17*		*C11orf17*
221065_s_at	*11.36*	*10.05*	*carbohydrate (N-acetylgalactosamine 4-0) sulfotransferase 8*		*CHST8*
221209_s_at	*-2.84*	*-1.89*	*otoraplin*	*NM_004481*	*OTOR*
221213_s_at	*1.87*	*2.09*	*zinc finger protein 280D*	*NM_015642*	*ZNF280D*
221261_x_at	*42.33*	*1.96*		*NM_002926 /// NM_198227 /// NM_198229*	
221294_at	*2.59*	*1.79*	*RAB GTPase activating protein 1*	*NM_002647*	*RABGAP1*
221349_at	*8.91*	*1.85*	*pre-B lymphocyte gene*	*NM_153223*	*VPREB1*
221600_s_at	*1.86*	*2.03*	*chromosome 11 open reading frame 67*		*C11orf67*
221696_s_at	*2.26*	*1.76*	*serine/threonine/tyrosin e kinase 1*	*NM_002919 /// NM_134428*	*STYK1*
221773_at	*2.18*	*1.89*	*ELK3, ETS-domain protein (SRF accessory*	*NM_170662*	*ELK3*
221892_at	*2.22*	*2.31*	*hexose-6-phosphate dehydrogenase (glucose 1-*	*NM_002350*	*H6PD*
221942_s_at	*2.35*	*2.29*	*guanylate cyclase 1, soluble, alpha 3*	*NM_030756*	*GUCY1A3*
222146_s_at	*1.75*	*2.15*	*transcription factor 4*	*NM_024870 /// NM_025170*	*TCF4*
222281_s_at	*1.84*	*2.85*		*NM_145307*	
222294_s_at	*2.36*	*2.12*		*NM_006017*	
222326_at	*2.30*	*2.09*	*phosphodiesterase 4B, cAMP-specific (phosphodiesterase E4 dunce homolog, Drosophila)*	*NM_003097 /// NM_022805 /// NM_022806 /// NM_022807 /// NM_022808*	*PDE4B*
222446_s_at	*-2.50*	*-2.31*	*beta-site APP-cleaving enzyme 2*	*NM_006557 /// NM_181872*	*BACE2*
222862_s_at	*1.94*	*2.08*	*adenylate kinase 5*	*NM_144660*	*AK5*

222880_at	1.82	1.94	v-akt murine thymoma viral oncogene homolog 3 (protein kinase B,	NM_139015	AKT3
223179_at	2.11	2.25	yippee-like 3 (Drosophila)	NM_001024912 /// NM_001712	YPEL3
223276_at	1.94	1.80			
223316_at	17.25	14.78	optineurin	NM_007118	OPTN
223371_s_at	-1.96	-2.04	DnaJ (Hsp40) homolog, subfamily C, member 4		DNAJC4
223399_x_at	2.47	5.49	polybromo 1	NM_015864	PBRM1
223444_at	2.30	2.01	SUMO1/sentrin specific peptidase 7	NM_031277	SENP7
223588_at	2.74	2.02	THAP domain containing, apoptosis associated protein 2	NM_017523 /// NM_199139	THAP2
223607_x_at	-2.01	-1.83	zinc finger, SWIM-type containing 1	NM_006851	ZSWIM1
223704_s_at	1.98	11.58	doublesex and mab-3 related transcription factor 2		DMRT2
223708_at	9.97	10.48	C1q and tumor necrosis factor related protein 4	NM_018660	C1QTNF4
223800_s_at	2.50	1.94		NM_004354	

223900_s_at	*2.96*	*2.74*	*polybromo 1*	*XM_927922 /// XM_928908 /// XM_932380 /// XM_932385 /// XM_932390 /// XM_932395 /// XM_932400 /// XM_932405 /// XM_932411 /// XM_932414 /// XM_933267 /// XM_933270 /// XM_933273 /// XM_933285 /// XM_933287 /// XM_933290 /// XM_933292 /// XM_935561 /// XM_935606 /// XM_935859 /// XM_935861 /// XM_935863 /// XM_935864 /// XM_935866 /// XM_935868 /// XM_935869 /// XM_935870 /// XM_935896 /// XM_935897 /// XM_935898 /// XM_935899 /// XM_935900 ///*	*PBRM1*
223952_x_at	*4.00*	*9.93*	*dehydrogenase/reductase (SDR family) member 9*	*NM_001032394 /// NM_001032395 /// NM_020455 /// NM_198569*	*DHRS9*
224009_x_at	*3.02*	*7.37*	*dehydrogenase/reductase (SDR family) member 9*	*NM_002847 /// NM_130842 /// NM_130843*	*DHRS9*
224328_s_at	*-2.51*	*-2.36*	*late cornified envelope*		*LCE3D*
224383_at	*1.97*	*11.00*	*ring finger protein 17*	*NM_003097 /// NM_022805 /// NM_022806 /// NM_022807 /// NM_022808*	*RNF17*
224402_s_at	*4.82*	*2.53*	*Fc receptor-like 4*	*NM_032947*	*FCRL4*
224403_at	*4.70*	*29.09*	*Fc receptor-like 4*	*NM_001025930 /// NM_015644*	*FCRL4*
224609_at	*2.20*	*1.85*	*solute carrier family 44, member 2*	*NM_130387*	*SLC44A2*

224799_at	*-2.27*	*-2.04*	*Nedd4 family interacting protein 2*	*NM_001006600 /// NM_018695*	*NDFIP2*
224836_at	*4.19*	*4.89*	*tumor protein p53*	*NM_152446*	*TP53INP2*
224917_at	*1.97*	*2.13*	*microRNA 21*	*NM_001037339 /// NM_001037340 /// NM_001037341 /// NM_002600*	*MIRN21*
224941_at	*-4.12*	*-2.78*	*pregnancy-associated plasma protein A,*	*NM_004585*	*PAPPA*
225056_at	*6.40*	*8.64*	*signal-induced proliferation-associated*	*NM_012093 /// NM_174858*	*SIPA1L2*
225129_at	*1.80*	*1.78*	*copine II*	*NM_006264 /// NM_080683 /// NM_080684 /// NM_080685*	*CPNE2*
225150_s_at	*15.10*	*1.99*	*rhotekin*	*NM_000699 /// NM_001008218 /// NM_001008219 /// NM_001008221 /// NM_004038 /// NM_020978 /// XM_936407 /// XM_937845 /// XM_942801*	*RTKN*
225166_at	*1.80*	*1.88*	*Rho GTPase activating protein 18*		*ARHGAP18*
225237_s_at	*1.87*	*1.92*	*musashi homolog 2 (Drosophila)*	*NM_020654*	*MSI2*
225239_at	*1.88*	*2.14*		*NM_004543*	
225273_at	*2.02*	*2.33*	*WWC family member 3*		*WWC3*
225583_at	*1.79*	*1.77*	*UDP-glucuronate decarboxylase 1*	*NM_004481*	*UXS1*
225688_s_at	*1.83*	*4.40*	*pleckstrin homology-like domain, family B, member 2*	*NM_012417 /// NM_181671*	*PHLDB2*
225706_at	*1.80*	*2.06*	*glucocorticoid induced transcript 1*	*NM_181504 /// NM_181523 /// NM_181524*	*GLCCI1*
225782_at	*2.06*	*1.84*	*methionine sulfoxide reductase B3*	*NM_001031804 /// NM_005360*	*MSRB3*
225798_at	*-2.00*	*-1.92*	*JAZF zinc finger 1*	*NM_000115 /// NM_003991*	*JAZF1*
225817_at	*28.71*	*337.00*	*cingulin-like 1*	*NM_015110*	*CGNL1*
225950_at	*1.98*	*2.57*	*sterile alpha motif domain containing 8*	*NM_001121 /// NM_016824 /// NM_019903*	*SAMD8*
226136_at	*1.89*	*3.00*		*NM_005410*	
226142_at	*1.96*	*2.89*	*GLI pathogenesis-related 1 (glioma)*	*XM_930500 /// XM_935094 /// XM_942528 /// XM_946076*	*GLIPR1*

226145_s_at	*4.10*	*4.44*	*Fraser syndrome 1*	*NM_015263*	*FRAS1*
226189_at	*2.04*	*3.40*	*integrin, beta 8*		*ITGB8*
226250_at	*2.00*	*1.89*			
226252_at	*1.92*	*2.02*			
226279_at	*6.73*	*3.53*	*protease, serine, 23*	*NM_173808*	*PRSS23*
226302_at	*3.49*	*2.52*		*NM_052905*	
226382_at	*1.89*	*2.80*		*NM_000110*	
226388_at	*2.94*	*2.70*	*transcription elongation factor A (SII), 3*	*NM_001001787 /// NM_001677*	*TCEA3*
226490_at	*2.30*	*4.27*	*NHS-like 1*	*NM_002299*	*NHSL1*
226657_at	*2.74*	*2.01*		*NM_138959*	
226725_at	*3.59*	*1.93*			
226757_at	*2.89*	*5.83*	*interferon-induced protein with*	*NM_015642*	*IFIT2*
226959_at	*2.11*	*1.88*			
227051_at	*-2.72*	*-3.33*		*NM_024841*	
227132_at	*1.81*	*1.81*	*zinc finger protein 706*	*NM_001723 /// NM_015548 /// NM_020388 /// NM_183380*	*ZNF706*
227174_at	*2.68*	*2.62*	*WD repeat domain 72*	*NM_152777*	*WDR72*
227195_at	*3.84*	*13.18*	*zinc finger protein 503*	*NM_002403 /// NM_017459*	*ZNF503*
227197_at	*6.17*	*5.31*		*XM_043272 /// XM_933967 /// XM_941783 /// XM_945439*	
227235_at	*2.57*	*2.80*	*guanylate cyclase 1, soluble, alpha 3*	*NM_005114*	*GUCY1A3*
227290_at	*1.80*	*1.82*		*NM_194291*	
227297_at	*2.42*	*2.31*	*integrin, alpha 9*	*NM_007001*	*ITGA9*
227312_at	*-2.72*	*-2.11*	*syntrophin, beta 2 (dystrophin-associated protein A1, 59kDa,*	*NM_001536 /// NM_198318 /// NM_198319*	*SNTB2*
227319_at	*3.33*	*2.10*			
227444_at	*1.90*	*14.05*	*armadillo repeat containing, X-linked 4*	*NM_005101*	*ARMCX4*
227529_s_at	*-2.87*	*-2.71*	*A kinase (PRKA) anchor protein (gravin) 12*		*AKAP12*
227530_at	*-2.30*	*-3.30*	*A kinase (PRKA) anchor protein (gravin) 12*	*NM_138962 /// NM_170721*	*AKAP12*
227609_at	*3.37*	*1.94*	*epithelial stromal interaction 1 (breast)*	*NM_001002843 /// NM_001002844 /// NM_017661*	*EPSTI1*
227834_at	*21.31*	*75.43*	*taxilin beta*	*NM_024684*	*TXLNB*
227839_at	*5.56*	*1.92*	*methyl-CpG binding domain protein 5*		*MBD5*
227929_at	*-1.88*	*-2.42*		*NM_152703*	
227949_at	*2.47*	*3.04*	*phosphatase and actin regulator 3*		*PHACTR3*
228081_at	*1.95*	*2.37*	*cyclin G2*	*NM_178527*	*CCNG2*

228108_at	*2.68*	*3.63*		*NM_019012*	
228128_x_at	*-3.95*	*-2.40*	*pregnancy-associated plasma protein A, pappalysin 1*	*NM_001121 /// NM_016824 /// NM_019903*	*PAPPA*
228174_at	*2.51*	*2.25*	*golgi autoantigen, golgin subfamily a, 1*	*NM_001423*	*GOLGA1*
228285_at	*3.09*	*9.42*	*tudor domain containing 9*	*XM_930677 /// XM_945048*	*TDRD9*
228297_at	*3.65*	*2.51*	*calponin 3, acidic*		*CNN3*
228574_at	*2.20*	*1.92*	*transmembrane and tetratricopeptide repeat containing 2*	*NM_006526*	*TMTC2*
228617_at	*2.45*	*2.04*	*XIAP associated factor*		*XAF1*
228779_at	*3.09*	*2.01*		*NM_004755 /// NM_182398*	
228788_at	*2.72*	*2.90*	*yippee-like 1 (Drosophila)*	*NM_004755 /// NM_182398*	*YPEL1*
228790_at	*13.90*	*4.01*	*family with sequence similarity 110, member*	*NM_002166*	*FAM110B*
228964_at	*2.67*	*2.73*	*PR domain containing 1, with ZNF domain*	*NM_000740*	*PRDM1*
228968_at	*1.81*	*1.78*	*zinc finger protein 449*	*NM_145753*	*ZNF449*
229011_at	*1.90*	*2.77*		*NM_001423*	
229057_at	*14.00*	*4.93*	*sodium channel, voltage-gated, type II, alpha subunit*	*NM_153704*	*SCN2A*
229090_at	*2.17*	*1.76*		*NM_019012*	
229134_at	*1.89*	*2.39*	*vang-like 1 (van gogh, Drosophila)*	*NM_004803*	*VANGL1*
229199_at	*2.26*	*4.75*		*NM_004354*	
229280_s_at	*2.37*	*2.06*		*NM_004449 /// NM_182918*	
229309_at	*1.76*	*2.41*			
229336_at	*-2.04*	*-2.35*	*ST3 beta-galactoside alpha-2,3-sialyltransferase 2*	*NM_005465 /// NM_181690*	*ST3GAL2*
229450_at	*3.44*	*2.93*		*NM_004580 /// NM_183234 /// NM_183235 /// NM_183236*	
229461_x_at	*5.68*	*5.49*	*neuronal growth*	*NM_018423*	*NEGR1*
229530_at	*3.07*	*2.30*	*guanylate cyclase 1, soluble, alpha 3*	*NM_030938*	*GUCY1A3*
229581_at	*-1.90*	*-3.91*	*extracellular leucine-rich repeat and fibronectin type III domain*	*NM_152695*	*ELFN1*
229623_at	*1.76*	*1.99*		*NM_001121 /// NM_016824 /// NM_019903*	
229638_at	*22.24*	*6.05*	*iroquois homeobox 3*	*NM_001039506 /// NM_006020*	*IRX3*

229756_at	1.83	3.96	inhibitor of DNA binding 2, dominant negative helix-loop-helix protein	NM_000969	ID2
230036_at	1.85	1.91	sterile alpha motif domain containing 9-like	NM_172004	SAMD9L
230147_at	-2.83	-3.38	coagulation factor II (thrombin) receptor-like 2	NM_001042510 /// NM_001042511 /// NM_016096	F2RL2
230224_at	2.62	2.13	mitochondrial carrier triple repeat 6		MCART6
230252_at	-1.75	-2.36	lysophosphatidic acid receptor 5	NM_018182	LPAR5
230302_at	10.69	1.94		NM_001042437 /// NM_003896	
230381_at	-2.28	-2.12	chromosome 1 open reading frame 186	NM_004892	C1orf186
230383_x_at	3.14	3.35		NM_001560	
230403_at	1.77	1.89		NM_002588 /// NM_003735 /// NM_003736 /// NM_014004 /// NM_018912 /// NM_018913 /// NM_018914 /// NM_018915 /// NM_018916 /// NM_018917 /// NM_018918 /// NM_018919 /// NM_018920 /// NM_018921 /// NM_018922 /// NM_018923 /// NM_018924 /// NM_018925 /// NM_018926 /// NM_018927 /// NM_018928 /// NM_018929 /// NM_031993 /// NM_032009 /// NM_032011 /// NM_032053 /// NM_032054 /// NM_032086 /// NM_032087 /// NM_032088 /// NM_032089 /// NM_032090 ///	
230469_at	1.99	2.43	rhotekin 2	NM_012199	RTKN2

230503_at	*-9.43*	*-2.66*	*sterile alpha motif domain containing 4A*	*NM_017640*	*SAMD4A*
230587_at	*5.45*	*6.02*			
230793_at	*1.80*	*1.94*	*leucine rich repeat containing 16A*	*NM_033515*	*LRRC16A*
230894_s_at	*1.90*	*4.53*			
230944_at	*-2.92*	*-2.07*	*chromosome 6 open reading frame 223*	*NM_138426*	*C6orf223*
231034_s_at	*2.60*	*2.25*		*NM_005465 /// NM_181690*	
231067_s_at	*-2.27*	*-1.85*	*A kinase (PRKA) anchor protein (gravin) 12*	*NM_152727*	*AKAP12*
231116_at	*1.95*	*2.09*	*chromosome 1 open reading frame 204*	*NM_000725*	*C1orf204*
231166_at	*-7.73*	*-2.03*	*G protein-coupled receptor 155*	*NM_006866*	*GPR155*
231697_s_at	*2.12*	*3.27*	*transmembrane protein*		*TMEM49*
231838_at	*1.85*	*1.88*	*poly(A) binding protein, cytoplasmic 1-like*	*NM_007085*	*PABPC1L*
231909_x_at	*3.75*	*2.95*	*outer dense fiber of sperm tails 2-like*	*NM_015864*	*ODF2L*
231929_at	*-2.52*	*-2.00*		*NM_004580 /// NM_183234 /// NM_183235 /// NM_183236*	
231964_at	*1.81*	*2.00*		*NM_024021 /// NM_148975*	
232098_at	*1.88*	*1.76*	*dystonin*	*NM_018349*	*DST*
232136_s_at	*-2.18*	*-1.87*	*cortactin binding protein*	*NM_025076*	*CTTNBP2*
232206_at	*-2.93*	*-2.20*	*unc-51-like kinase 4 (C. elegans)*	*NM_001015880 /// NM_004670*	*ULK4*
232212_at	*-3.10*	*-2.24*	*pleckstrin homology domain containing, family A (phosphoinositide binding specific) member 8*	*NM_001033569 /// NM_001033570 /// NM_001033571 /// NM_001033572 /// NM_001033574 /// NM_016627*	*PLEKHA8*
232252_at	*2.61*	*2.96*	*dual specificity phosphatase 27*	*NM_006298*	*DUSP27*
232297_at	*1.74*	*2.31*		*NM_024503*	
232465_at	*2.77*	*2.28*	*transcription factor 7-like 2 (T-cell specific,*	*NM_004566*	*TCF7L2*
232610_at	*2.03*	*2.07*	*poly (ADP-ribose) polymerase family,*	*NR_002720*	*PARP14*
232636_at	*4.27*	*4.16*	*SLIT and NTRK-like family, member 4*	*NM_001039697*	*SLITRK4*
232914_s_at	*2.13*	*2.36*	*synaptotagmin-like 2*		*SYTL2*
232959_at	*-2.16*	*-2.63*		*NM_001005176 /// NM_007237*	

233020_at	*1.80*	*2.46*	*SEC22 vesicle trafficking protein homolog B (S.*		*SEC22B*
233051_at	*-6.91*	*-1.94*	*SLIT and NTRK-like family, member 2*	*NM_003793*	*SLITRK2*
233079_at	*3.46*	*5.09*	*c-mer proto-oncogene tyrosine kinase*		*MERTK*
233246_at	*2.20*	*1.86*	*chromosome 9 open reading frame 126*	*NM_006379*	*C9orf126*
233296_x_at	*1.89*	*1.90*	*zinc finger and BTB domain containing 20*	*NM_000443 /// NM_000927 /// NM_018849 /// NM_018850*	*ZBTB20*
233377_at	*2.53*	*2.27*	*AT rich interactive domain 5B (MRF1-like)*	*NM_198281*	*ARID5B*
233430_at	*-5.37*	*-2.36*	*TBC1 domain family, member 22B*	*NM_145804*	*TBC1D22B*
233587_s_at	*5.13*	*5.05*	*signal-induced proliferation-associated*	*XM_496707 /// XM_938685*	*SIPA1L2*
233614_at	*2.00*	*1.79*	*Cas-Br-M (murine) ecotropic retroviral transforming sequence*		*CBLB*
233627_at	*-2.17*	*-1.81*	*megakaryoblastic*	*NM_003253*	*MKL1*
233691_at	*1.76*	*2.25*	*ankyrin repeat and BTB (POZ) domain containing 2*	*NM_001121 /// NM_016824 /// NM_019903*	*ABTB2*
234125_at	*1.94*	*1.92*	*erbb2 interacting protein*	*NM_022089*	*ERBB2IP*
234217_at	*-2.30*	*-2.12*	*GDP-mannose 4,6-dehydratase*	*NM_178173*	*GMDS*
234563_at	*6.04*	*17.49*	*phosphodiesterase 3A, cGMP-inhibited*	*NM_003199*	*PDE3A*
234805_at	*-2.95*	*-2.45*	*ATPase family, AAA domain containing 1*	*NM_001062*	*ATAD1*
234862_at	*2.47*	*2.04*			
235046_at	*7.05*	*5.92*		*NM_004612*	
235212_at	*8.64*	*8.47*	*chromosome 14 open reading frame 102*	*NM_005407*	*C14orf102*
235264_at	*2.07*	*2.36*		*NM_020400*	
235401_s_at	*3.23*	*14.14*	*Fc receptor-like A*	*XM_373347 /// XM_937675*	*FCRLA*
235422_at	*1.79*	*2.57*	*archaelysin family metallopeptidase 2*	*NM_012105 /// NM_138991 /// NM_138992*	*AMZ2*
235456_at	*1.84*	*2.02*		*NM_000072 /// NM_001001547 /// NM_001001548*	
235754_at	*-2.05*	*-3.30*	*hemochromatosis*	*NM_001935*	*HFE*
235764_at	*2.10*	*2.79*	*PR domain containing 5*	*NM_005723*	*PRDM5*
235766_x_at	*2.36*	*2.44*		*NM_003542*	

235775_at	2.85	2.48	transmembrane and tetratricopeptide repeat containing 2	NM_006093	TMTC2
236034_at	3.56	38.22		NM_001640	
236199_at	-2.28	-4.38	arachidonate 5-lipoxygenase	NM_000517 /// NM_000558	ALOX5
236565_s_at	8.88	5.95	La ribonucleoprotein domain family, member 6	NM_019624 /// NM_019625 /// NM_203444 /// NM_203445	LARP6
236721_at	1.81	2.26	alkB, alkylation repair homolog 1 (E. coli)	NM_000362	ALKBH1
236738_at	2.87	4.32		NM_198098	
236832_at	1.91	2.05		NM_024692	
237061_at	2.23	1.76	zinc finger protein 347	NM_002704	ZNF347
237261_at	17.21	1.83		NM_006563	
237444_at	1.82	2.06			
237446_at	-1.91	-7.55		NM_182529	
237483_at	3.03	2.58	pleckstrin homology domain containing, family A member 5	NM_000238 /// NM_172056 /// NM_172057	PLEKHA5
237514_at	27.93	7.62		XM_374386 /// XM_935649 /// XM_941372	
237725_x_at	1.91	2.02	structural maintenance of chromosomes 5		SMC5
237917_at	2.34	1.83	neuroblastoma	NM_005528	NBPF9
238032_at	2.12	2.37	dehydrogenase/reductase (SDR family)	NM_175619 /// XM_941237	DHRS3
238045_at	1.88	2.08	transmembrane protein 65	NM_000184 /// NM_000559	TMEM65
238154_at	2.06	2.15	centrosomal protein		CEP70
238174_at	2.45	1.77	F-box and leucine-rich repeat protein 17	NM_000477	FBXL17
238292_at	-2.28	-4.28	SFT2 domain containing	NM_005332	SFT2D1
238497_at	3.05	5.27	transmembrane protein 136	NM_175061	TMEM136
238614_x_at	2.08	2.26	zinc finger protein 430	NM_002662	ZNF430
238649_at	4.37	2.28	phosphatidylinositol transfer protein, cytoplasmic 1	NM_003711 /// NM_176895	PITPNC1
238668_at	2.01	2.18		NM_182568 /// XM_930399 /// XM_934726 /// XM_934727 /// XM_934728 /// XM_934729	
238692_at	2.03	3.57	BTB (POZ) domain containing 11	NM_080603	BTBD11
238874_at	3.78	4.77		NM_000519	
239036_at	1.80	1.96	eukaryotic translation initiation factor 2C, 1	NM_006927	EIF2C1

239205_s_at	-6.67	-2.84	complement component (3b/4b) receptor 1 (Knops blood group)	NM_000410 /// NM_139002 /// NM_139003 /// NM_139004 /// NM_139005 /// NM_139006 /// NM_139007 /// NM_139008 /// NM_139009 /// NM_139010 /// NM_139011	CR1
239272_at	2.20	4.60	matrix metallopeptidase 28	NM_000573 /// NM_000651	MMP28
239287_at	4.47	2.89			
239300_at	2.00	2.14	phosphoinositide-3-kinase, class 3	NM_030758	PIK3C3
239387_at	2.38	1.97	UV radiation resistance associated gene	NM_000395	UVRAG
239476_at	1.92	2.02	phosphoinositide-3-kinase, regulatory	NM_003236	PIK3R1
239529_at	-2.60	-1.90	chromosome 5 open reading frame 20	NM_000517 /// NM_000558	C5orf20
239555_at	1.99	1.88	v-yes-1 Yamaguchi sarcoma viral related oncogene homolog	NM_000518	LYN
239559_at	1.83	3.31	pleckstrin homology domain containing, family A member 5	NM_020458	PLEKHA5
239605_x_at	1.74	1.86	transforming growth factor, beta receptor I (activin A receptor type II-like kinase, 53kDa)	XM_027236 /// XM_938197	TGFBR1
239619_at	1.95	1.91	zinc finger protein 395	NM_000517 /// NM_000558	ZNF395
239625_at	23.29	2.56		NM_000304 /// NM_153321 /// NM_153322	
239808_at	1.92	1.91	phosphatidylinositol transfer protein, cytoplasmic 1	NM_002619	PITPNC1
239872_at	-2.08	-2.05	oxysterol binding protein	NM_207332	OSBP2
239893_at	1.79	1.85	multiple C2 domains, transmembrane 2	NM_018717	MCTP2
239958_at	3.47	6.94			
240000_at	1.77	4.17		NM_020831	
240173_at	2.17	7.22		NM_033427	
240204_at	1.99	1.80	small nuclear ribonucleoprotein polypeptide N	NM_000698	SNRPN
240380_at	2.17	1.83		NM_001828	
240399_at	1.98	2.11	triple functional domain (PTPRF interacting)	NM_012074	TRIO

240432_x_at	*2.20*	*1.78*		*NM_005100 /// NM_144497*	
240454_at	*-2.11*	*-2.18*	*tetratricopeptide repeat domain 7A*	*NM_019080*	*TTC7A*
240566_at	*4.31*	*2.17*		*NM_000698*	
240568_at	*1.80*	*2.28*	*v-akt murine thymoma viral oncogene homolog 3 (protein kinase B,*	*NM_145169*	*AKT3*
240630_at	*2.05*	*1.93*	*retinoic acid receptor, alpha*	*NM_003956*	*RARA*
240696_at	*-3.39*	*-2.24*		*NM_001007544*	
240728_at	*3.13*	*3.57*	*phospholipase C, beta 4*	*NM_005100 /// NM_144497*	*PLCB4*
240782_at	*13.61*	*21.86*		*NM_000558*	
240815_at	*1.77*	*2.81*	*sema domain, immunoglobulin domain (Ig), short basic domain, secreted, (semaphorin)*	*NM_001500*	*SEMA3C*
240867_at	*2.00*	*2.36*	*regulatory factor X, 3 (influences HLA class II expression)*	*NM_000184 /// NM_000559*	*RFX3*
240878_at	*6.91*	*5.30*	*fibroblast growth factor 11*	*NM_000184 /// NM_000559*	*FGF11*
240882_at	*61.55*	*7.58*		*NM_006902 /// NM_022716*	
240948_at	*5.17*	*2.28*	*toll-like receptor 4*	*NM_003711 /// NM_176895*	*TLR4*
240977_at	*2.18*	*2.57*	*leucine-rich repeats and calponin homology (CH) domain containing 1*		*LRCH1*
241005_at	*-3.78*	*-8.34*		*NM_014761*	
241060_x_at	*-1.76*	*-2.02*	*tetraspanin 5*	*NM_014779*	*TSPAN5*
241216_at	*2.01*	*2.46*	*kinesin family member*	*NM_020125*	*KIF1B*
241304_at	*2.60*	*2.84*		*NM_000518*	
241497_at	*2.25*	*5.70*		*NM_005100 /// NM_144497*	
241564_at	*-1.75*	*-2.35*	*glyoxalase domain containing 5*	*NM_012105 /// NM_138991 /// NM_138992*	*GLOD5*
241646_s_at	*2.05*	*3.91*		*NM_032563*	
241730_at	*1.77*	*2.10*			
241754_at	*2.43*	*1.91*	*chromosome 9 open reading frame 126*	*NM_000518*	*C9orf126*
241823_at	*-1.98*	*-1.82*		*NM_002112*	
241837_at	*3.10*	*2.57*	*AT rich interactive domain 5B (MRF1-like)*	*NM_001017927*	*ARID5B*
242002_at	*2.76*	*3.80*	*Na+/K+ transporting ATPase interacting 2*	*NM_130848*	*NKAIN2*
242015_x_at	*-1.79*	*-1.99*	*N-acylaminoacyl-peptide hydrolase*	*NM_003855*	*APEH*
242079_at	*2.00*	*2.13*	*regulator of G-protein signaling 12*		*RGS12*

242088_at	2.83	1.89	kelch-like 24	NM_006052	KLHL24
242134_at	2.07	2.15		NM_003711 /// NM_176895	
242197_x_at	-3.51	-1.85	CD36 molecule (thrombospondin receptor)	NM_020485 /// NM_138616 /// NM_138617 /// NM_138618	CD36
242234_at	1.97	2.90	XIAP associated factor	NM_006750	XAF1
242321_at	6.15	34.00			
242405_at	2.73	2.26	mastermind-like 2 (Drosophila)	NM_000517	MAML2
242416_at	2.03	1.75	protein tyrosine phosphatase, receptor type, G	XM_496963 /// XM_933431 /// XM_936749 /// XM_942939	PTPRG
242488_at	3.63	2.84		NM_000517 /// NM_000558	
242559_at	23.22	2.31	serine palmitoyltransferase, long chain base subunit	NM_001430	SPTLC3
242625_at	2.29	1.78	radical S-adenosyl methionine domain containing 2	NM_004101	RSAD2
242665_at	1.90	2.01	formin-like 2	NM_020157	FMNL2
242681_at	3.01	2.13	NAD(P)H dehydrogenase,	NM_005100 /// NM_144497	NQO1
242798_at	-2.38	-1.77		NM_001870	
242868_at	-2.83	-2.43	endothelial PAS domain protein 1	NM_153246	EPAS1
242873_at	5.73	2.54	killer cell lectin-like receptor subfamily C, member 4	XM_929989 /// XM_934158 /// XM_934164 /// XM_934167 /// XM_934168 /// XM_934169 /// XM_934170 /// XM_934171 /// XM_934172 /// XM_934174 /// XM_942055 /// XM_945658 /// XM_945660 /// XM_945662 /// XM_945665 /// XM_945667 /// XM_945669 /// XM_945672 /// XM_945673 /// XM_945675 /// XM_945676	KLRC4

242876_at	*2.14*	*2.45*	*v-akt murine thymoma viral oncogene homolog 3 (protein kinase B,*	*NM_032810*	*AKT3*
242907_at	*3.56*	*5.35*	*guanylate binding protein 2, interferon-*	*NM_018584*	*GBP2*
243006_at	*2.14*	*1.96*	*FYN oncogene related to SRC, FGR, YES*	*NM_032639*	*FYN*
243050_at	*-2.14*	*-2.33*	*peripheral myelin protein 22*	*NM_003864*	*PMP22*
243221_at	*4.01*	*2.47*	*family with sequence similarity 20, member A*	*NM_005596*	*FAM20A*
243357_at	*1.90*	*3.48*	*neuronal growth*		*NEGR1*
243386_at	*3.66*	*7.48*		*NM_000072 /// NM_001001547 /// NM_001001548*	
243715_at	*-2.01*	*-2.57*	*phosphatidic acid phosphatase type 2A*	*NM_005020*	*PPAP2A*
243808_at	*2.03*	*2.18*	*cyclin-dependent kinase*		*CDK6*
243886_at	*-2.56*	*-2.03*		*NM_002581*	
243915_at	*3.19*	*4.41*	*archaelysin family metallopeptidase 2*	*NM_002581*	*AMZ2*
244190_at	*-1.88*	*-1.88*	*THAP domain*	*NM_000502*	*THAP5*
244236_at	*1.80*	*2.12*	*chromosome 17 open reading frame 63*		*C17orf63*
244291_x_at	*1.84*	*2.04*		*NM_017772*	
244359_s_at	*4.97*	*4.48*		*NM_139265*	
244413_at	*1.81*	*2.80*	*C-type lectin-like 1*	*NM_033414*	*CLECL1*
244533_at	*2.37*	*5.68*		*NM_000573 /// NM_000651 /// XM_114735 /// XM_931252 /// XM_931256 /// XM_936496 /// XM_936931 /// XM_943074 /// XM_943081*	
244551_at	*2.20*	*1.88*			
244762_at	*-2.68*	*-12.49*	*Down syndrome critical region gene 3*	*NM_032539*	*DSCR3*
244764_at	*1.78*	*2.31*	*human immunodeficiency virus type I enhancer binding*	*NM_001033045 /// NM_152529*	*HIVEP3*
266_s_at	*9.67*	*10.14*	*CD24 molecule*	*NM_000573 /// NM_000651*	*CD24*
34408_at	*2.27*	*1.75*	*reticulon 2*	*NM_015589*	*RTN2*
34726_at	*1.80*	*1.79*	*calcium channel, voltage-dependent, beta*	*NM_152636*	*CACNB3*

35626_at	*4.11*	*2.90*	*N-sulfoglucosamine sulfohydrolase (sulfamidase)*	*NM_000037 /// NM_020475 /// NM_020476 /// NM_020477 /// NM_020478 /// NM_020479 /// NM_020480 /// NM_020481*	*SGSH*
35776_at	*2.35*	*2.53*	*intersectin 1 (SH3 domain protein)*	*NM_001039654*	*ITSN1*
38037_at	*2.08*	*2.13*	*heparin-binding EGF-like growth factor*		*HBEGF*
41387_r_at	*1.88*	*2.07*	*jumonji domain containing 3, histone lysine demethylase*	*NM_004815*	*JMJD3*
44790_s_at	*7.24*	*21.64*	*chromosome 13 open reading frame 18*		*C13orf18*
