# Supplementary material for: Dissection of the Transformation of Primary Human Hematopoietic Cells by the Oncogene NUP98-HOXA9
Source: PLoS One. 2009 Aug 21;4(8):e6719. doi: 10.1371/journal.pone.0006719 (PMC2725295; doi:10.1371/journal.pone.0006719)
Supplement: Table S5 — Genes dysregulated by HOXA9ΔN. Primary human CD34+ cells were retrovirally transduced with either control MSCV-IRES-GFP vector or vector expressing HOXA9ΔN. Cells were sorted for GFP positivity and total RNA was subjected to microarray analysis. The experiment was performed two independent times and only genes that showed up- or down-regulation by 1.74 fold or more compared to control in both experiments were considered dysregulated. (0.03 MB PDF) [file pone.0006719.s005.pdf]

Table S5. Genes dysregulated by HOXA9ΔN

Probeset I.D.	Fold Change		Gene Name	Accession Number	Gene Symbol
	Exp.1	*Exp.2*			
243130_at	*58.30*	*6.93*			
230386_at	*14.50*	*3.20*			
1557349_at	*14.10*	*18.96*	*arginine-glutamic acid*	*NM_001042681*	*RERE*
1564642_at	*12.62*	*15.90*	*runt-related transcription factor 1; translocated to, 1 (cyclin D-related)*	*NM_004349 /// NM_175634 /// NM_175635 /// NM_175636*	*RUNX1T1*
224123_at	*8.91*	*7.26*			
240878_at	*8.30*	*3.89*	*fibroblast growth factor 11*	*NM_199339*	*FGF11*
204475_at	*8.27*	*4.87*	*matrix metallopeptidase 1 (interstitial collagenase)*	*NM_002421*	*MMP1*
1570119_at	*6.96*	*2.91*	*PDS5, regulator of cohesion maintenance, homolog B (S. cerevisiae)*	*NM_015032*	*PDS5B*
236892_s_at	*6.69*	*2.40*			
237058_x_at	*6.65*	*12.84*	*solute carrier family 6 (neurotransmitter transporter, GABA), member 13*	*NM_016615*	*SLC6A13*
227566_at	*6.45*	*2.54*		*NM_016522*	
1560819_a_at	*6.28*	*4.05*		*XM_373553*	
210228_at	*6.19*	*5.73*	*colony stimulating factor 2 (granulocyte-macrophage)*	*NM_000758*	*CSF2*
234520_at	*5.80*	*25.82*			
1562058_at	*5.75*	*2.05*			
1564259_at	*5.03*	*2.15*	*ubiquitin specific peptidase 2*	*NM_004205 /// NM_171997*	*USP2*
1558982_at	*4.83*	*2.24*		*XM_927556*	
216833_x_at	*4.68*	*2.38*	*glycophorin B (MNS blood group)*	*NM_002100 /// NM_002102 /// NM_198682*	*GYPB*
236284_at	*4.29*	*5.52*	*KIAA0146*		*KIAA0146*
230500_at	*4.26*	*1.92*			
1554205_s_at	*4.24*	*2.63*	*islet cell autoantigen 1,69kDa-like*	*NM_138468 /// NM_178231*	*ICA1L*
214239_x_at	*4.22*	*1.97*	*polycomb group ring finger 2*	*NM_007144*	*PCGF2*
224040_at	*4.04*	*1.80*	*testis-specific transcript, Y-linked 5*	*NR_001541*	*TTTY5*
207326_at	*4.03*	*8.41*	*betacellulin*	*NM_001729*	*BTC*
206077_at	*3.97*	*2.24*	*Kell blood group, metallo-endopeptidase*	*NM_000420*	*KEL*
234097_s_at	*3.82*	*7.03*	*chromosome 6 open reading frame 12*	*XM_379403 /// XM_945157*	*C6orf12*
216020_at	*3.69*	*1.91*	*interferon induced with helicase C domain 1*	*NM_022168*	*IFIH1*

1564158_a_at	*3.65*	*25.39*	*Vac14 homolog (S. cerevisiae)*	*NM_018052*	*VAC14*
1556903_at	*3.37*	*1.89*	*midline 2*	*NM_012216 /// NM_052817*	*MID2*
221538_s_at	*3.26*	*2.03*	*plexin A1*	*NM_032242*	*PLXNA1*
208462_s_at	*3.23*	*3.52*	*ATP-binding cassette, sub-family C (CFTR/MRP), member 9*	*NM_005691 /// NM_020297 /// NM_020298*	*ABCC9*
240539_at	*3.20*	*1.88*	*autism susceptibility candidate 2*	*NM_015570*	*AUTS2*
239712_at	*3.19*	*3.96*			
229199_at	*3.18*	*2.85*			
1562741_at	*3.16*	*2.04*	*UBX domain containing 2*	*NM_014607*	*UBXD2*
241516_at	*3.13*	*1.76*	*solute carrier family 5 (sodium/glucose cotransporter), member 12*	*NM_001042366 /// NM_178498*	*SLC5A12*
240728_at	*3.10*	*3.37*	*phospholipase C, beta 4*	*NM_000933 /// NM_182797*	*PLCB4*
1554549_a_at	*3.05*	*1.87*	*WD repeat domain 20*	*NM_144574 /// NM_181291 /// NM_181302 /// NM_181308*	*WDR20*
1557149_at	*3.00*	*1.87*			
236599_at	*3.00*	*1.85*	*serpin peptidase inhibitor, clade E (nexin, plasminogen*	*NM_006216*	*SERPINE2*
216509_x_at	*3.00*	*2.17*	*myeloid/lymphoid or mixed-lineage leukemia (trithorax homolog, Drosophila); translocated to, 10*	*NM_001009569 /// NM_004641*	*MLLT10*
243250_at	*2.99*	*1.91*	*zinc finger, DHHC-type containing 13*	*NM_001001483 /// NM_019028*	*ZDHHC13*
1555122_at	*2.93*	*2.12*	*G protein-coupled receptor 125*	*NM_145290*	*GPR125*
240399_at	*2.83*	*2.05*	*triple functional domain*	*NM_007118*	*TRIO*
203485_at	*2.81*	*1.74*	*reticulon 1*	*NM_021136 /// NM_206852 /// NM_206857*	*RTN1*
218330_s_at	*2.73*	*1.78*	*neuron navigator 2*	*NM_145117 /// NM_182964*	*NAV2*
240564_x_at	*2.72*	*2.23*	*synuclein, alpha (non A4 component of amyloid precursor)*	*NM_000345 /// NM_007308*	*SNCA*
213348_at	*2.71*	*1.92*	*cyclin-dependent kinase*	*NM_000076*	*CDKN1C*
1568777_at	*2.71*	*5.04*	*echinoderm microtubule*	*NM_183387*	*EML5*
1560058_at	*2.71*	*2.06*		*NM_001013667*	
215810_x_at	*2.63*	*2.08*	*dystonin*	*NM_001723 /// NM_015548 /// NM_020388 /// NM_183380*	*DST*

219411_at	*2.60*	*2.25*	*engulfment and cell motility 3*	*NM_024712*	*ELMO3*
206595_at	*2.58*	*3.64*	*cystatin E/M*	*NM_001323*	*CST6*
215852_x_at	*2.57*	*1.76*	*chromosome 20 open reading frame 117*	*NM_199181*	*C20orf117*
1568646_x_at	*2.57*	*3.63*	*zinc finger protein 208*	*NM_007153*	*ZNF208*
209442_x_at	*2.55*	*1.87*	*ankyrin 3, node of Ranvier (ankyrin G)*	*NM_001149 /// NM_020987*	*ANK3*
242805_at	*2.49*	*1.76*	*cyclin C*	*NM_001013399 /// NM_005190*	*CCNC*
1555159_at	*2.38*	*4.62*	*transmembrane protein 74*	*NM_153015*	*TMEM74*
211479_s_at	*2.37*	*4.00*	*5-hydroxytryptamine*	*NM_000868*	*HTR2C*
207754_at	*2.35*	*1.87*	*Ras association (RalGDS/AF-6) domain family (N-terminal) member 8*	*NM_007211*	*RASSF8*
223995_at	*2.35*	*1.88*	*solute carrier family 12 (potassium/chloride*	*NM_020246*	*SLC12A9*
1553493_a_at	*2.32*	*24.45*	*L-threonine dehydrogenase*	*NR_001578*	*TDH*
235617_x_at	*2.31*	*3.47*			
244424_at	*2.31*	*2.73*			
243396_at	*2.29*	*2.26*			
230706_s_at	*2.29*	*1.79*	*calcium/calmodulin-dependent protein kinase II inhibitor 2*	*NM_033259*	*CAMK2N2*
219019_at	*2.29*	*1.83*	*leucine-rich repeats and death*	*NM_018494 ///*	*LRDD*
225540_at	*2.26*	*1.80*	*microtubule-associated protein 2*	*NM_001039538 /// NM_002374 /// NM_031845 /// NM_031847*	*MAP2*
201269_s_at	*2.25*	*3.53*	*NudC domain containing 3*	*NM_015332*	*NUDCD3*
1564121_at	*2.24*	*1.97*	*active BCR-related gene*	*NM_001092 /// NM_021962*	*ABR*
216341_s_at	*2.22*	*19.83*	*gonadotropin-releasing hormone receptor*	*NM_000406 /// NM_001012763*	*GNRHR*
230812_at	*2.21*	*1.84*			
227599_at	*2.18*	*1.92*	*chromosome 3 open reading frame 59*	*NM_178496*	*C3orf59*
220891_at	*2.17*	*2.35*	*chromosome 4 open reading frame 23*	*NM_152544*	*C4orf23*
216813_at	*2.17*	*1.74*			
235541_at	*2.12*	*2.62*	*LAS1-like (S. cerevisiae)*	*NM_031206*	*LAS1L*
231172_at	*2.12*	*3.65*	*chromosome 9 open reading frame 117*	*NM_001012502*	*C9orf117*
243746_at	*2.12*	*2.78*			
235844_at	*2.12*	*2.28*	*putative homeodomain*	*NM_006608*	*PHTF1*
1553533_at	*2.09*	*2.55*	*junctophilin 1*	*NM_020647*	*JPH1*
1570111_at	*2.09*	*4.17*	*chromosome 14 open reading frame 48*	*NM_152777*	*C14orf48*

240229_at	*2.08*	*2.23*	*G protein-coupled receptor*	*NM_014030*	*GIT1*
64899_at	*2.07*	*2.27*		*NM_022737*	
212698_s_at	*2.06*	*3.27*	*septin 10*	*NM_144710 /// NM_178584*	*10-Sep*
221800_s_at	*2.05*	*1.86*	*chromosome 17 open reading frame 70*	*NM_025161*	*C17orf70*
226722_at	*2.04*	*1.86*	*family with sequence similarity*	*NM_020223*	*FAM20C*
211269_s_at	*2.04*	*2.56*	*interleukin 2 receptor, alpha*	*NM_000417*	*IL2RA*
1563012_x_at	*2.02*	*1.78*			
217484_at	*2.00*	*4.18*	*complement component (3b/4b) receptor 1 (Knops blood group)*	*NM_000573 /// NM_000651*	*CR1*
225085_at	*2.00*	*1.88*	*ubiquitin specific peptidase 40*	*NM_018218*	*USP40*
222075_s_at	*1.99*	*2.38*	*ornithine decarboxylase antizyme 3*	*NM_016178*	*OAZ3*
1566624_at	*1.98*	*1.77*	*solute carrier family 1 (glial high affinity glutamate transporter), member 3*	*NM_004172*	*SLC1A3*
217406_at	*1.98*	*3.92*		*XM_929562 /// XM_939618*	
1570269_at	*1.98*	*2.40*			
1560706_at	*1.98*	*3.28*	*neural precursor cell expressed, developmentally down-regulated 9*	*NM_006403 /// NM_182966*	*NEDD9*
1568745_at	*1.97*	*2.65*		*XM_933506 /// XM_943630*	
243006_at	*1.96*	*1.91*	*FYN oncogene related to SRC,*	*NM_002037 ///*	*FYN*
1569953_at	*1.95*	*3.60*			
207939_x_at	*1.94*	*1.81*	*RNA binding protein S1, serine-*	*NM_006711 ///*	*RNPS1*
206341_at	*1.94*	*2.28*	*interleukin 2 receptor, alpha*	*NM_000417*	*IL2RA*
205050_s_at	*1.92*	*2.10*	*mitogen-activated protein kinase 8 interacting protein 2*	*NM_012324 /// NM_016431 /// NM_139124*	*MAPK8IP2*
1553626_a_at	*1.90*	*1.78*	*chromosome 17 open reading frame 57*	*NM_152347*	*C17orf57*
239347_at	*1.90*	*1.76*			
205898_at	*1.89*	*2.13*	*chemokine (C-X3-C motif) receptor 1*	*NM_001337*	*CX3CR1*
213849_s_at	*1.89*	*2.00*	*protein phosphatase 2 (formerly 2A), regulatory subunit B, beta isoform*	*NM_004576 /// NM_181674 /// NM_181675 /// NM_181676 /// NM_181677 /// NM_181678*	*PPP2R2B*
230452_at	*1.87*	*2.22*			
237764_at	*1.87*	*1.76*			
229739_s_at	*1.86*	*2.60*	*family with sequence similarity*	*NM_001001794*	*FAM116B*
223291_at	*1.85*	*3.00*	*mitochondrial ribosomal protein S15*	*NM_031280*	*MRPS15*
237210_at	*1.84*	*2.67*	*nuclear factor related to*	*NM_006165*	*NFRKB*
215062_at	*1.84*	*2.78*	*formin-like 2*	*NM_052905*	*FMNL2*

228297_at	1.84	1.86	calponin 3, acidic	NM_001839	CNN3
241216_at	1.83	2.08	kinesin family member 1B	NM_015074 /// NM_183416	KIF1B
207056_s_at	1.82	2.00	solute carrier family 4, sodium bicarbonate cotransporter, member 8	NM_001039960 /// NM_004858	SLC4A8
224063_at	1.81	1.82	neurolysin (metallopeptidase M3 family)	NM_020726	NLN
235635_at	1.81	2.19	Rho GTPase activating protein 5	NM_001030055 /// NM_001173	ARHGAP5
216752_at	1.80	4.23	phosphoinositide-3-kinase,	NM_014602	PIK3R4
237583_at	1.78	3.69			
1562988_at	1.78	2.06		XM_929815 /// XM_937547	
1553828_at	1.78	1.76	family with sequence similarity	NM_152315	FAM55D
1564468_at	1.76	2.14		NM_017884	
212012_at	1.76	1.86	peroxidasin homolog (Drosophila)	NM_012293	PXDN
243318_at	1.75	1.89	WD repeat domain 42A	NM_015726	WDR42A
240659_x_at	1.75	2.12	chromosome 14 open reading frame 106	NM_018353	C14orf106
1569672_at	1.74	2.84			
238575_at	-1.74	-1.84	oxysterol binding protein-like 6	NM_032523 /// NM_145739	OSBPL6
207017_at	-1.75	-2.99	RAB27B, member RAS oncogene family	NM_001001713 /// NM_004163 /// NM_007341	RAB27B
228772_at	-1.76	-11.53	histamine N-methyltransferase	NM_001024074 /// NM_001024075 /// NM_006895	HNMT
202478_at	-1.76	-3.33	tribbles homolog 2 (Drosophila)	NM_021643	TRIB2
1560171_at	-1.76	-2.57	SNF2 histone linker PHD RING helicase	NM_001042683 /// NM_173082	SHPRH
229202_at	-1.78	-1.83			
1558755_x_at	-1.78	-1.86	zinc finger protein 763	NM_001012753	ZNF763
1562474_at	-1.80	-1.75			
234956_at	-1.81	-3.46			
211424_x_at	-1.81	-2.38	methyltransferase like 7A	NM_014033	METTL7A
242014_at	-1.85	-2.93	zinc finger, RAN-binding	NM_032143	ZRANB3
214696_at	-1.85	-1.80	chromosome 17 open reading frame 91	NM_001001870 /// NM_032895	C17orf91
204627_s_at	-1.85	-2.35	integrin, beta 3 (platelet glycoprotein IIIa, antigen CD61)	NM_000212	ITGB3

243032_at	-1.85	-2.07		NM_015553	
230904_at	-1.90	-1.91	fibronectin type III and SPRY domain containing 1-like	NM_031919 /// NM_207647	FSD1L
1569013_s_at	-1.91	-1.80		XM_497054 /// XM_926051 /// XM_929002 /// XM_930920 /// XM_930928 /// XM_933343 /// XM_933346 /// XM_933352 /// XM_933364 /// XM_933367 /// XM_933371 /// XM_933373	
238307_at	-1.92	-2.06			
206932_at	-1.93	-3.22	cholesterol 25-hydroxylase	NM_003956	CH25H
1560023_x_at	-1.95	-1.85			
234805_at	-1.96	-1.94	ATPase family, AAA domain containing 1	NM_032810	ATAD1
227151_at	-1.97	-2.94	sorting nexin 33	NM_153271	SNX33
1565898_at	-1.98	-2.18	methyltransferase 5 domain containing 1	NM_152636	METT5D1
233627_at	-1.99	-4.04	megakaryoblastic leukemia	NM_020831	MKL1
1556697_at	-2.00	-15.95	GPRIN family member 3	NM_198281	GPRIN3
240557_at	-2.02	-1.82	TSC22 domain family, member 2	NM_014779	TSC22D2
228715_at	-2.03	-3.24	zinc finger, CCHC domain containing 12	NM_173798	ZCCHC12
235823_at	-2.07	-5.81	acyl-CoA synthetase family member 3	NM_174917	ACSF3
234168_at	-2.09	-2.04	TAF15 RNA polymerase II, TATA box binding protein	NM_003487 /// NM_139215	TAF15
209298_s_at	-2.11	-2.15	intersectin 1 (SH3 domain protein)	NM_001001132 /// NM_003024	ITSN1
203910_at	-2.11	-3.05	Rho GTPase activating protein 29	NM_004815	ARHGAP29
235218_x_at	-2.16	-1.84	THAP domain containing 6	NM_144721	THAP6
235044_at	-2.18	-2.02	cysteine/tyrosine-rich 1	NM_052954	CYYR1
231071_at	-2.20	-1.75			
227529_s_at	-2.22	-2.12	A kinase (PRKA) anchor	NM_005100 ///	AKAP12
238483_at	-2.23	-1.93			
203431_s_at	-2.24	-2.26		NM_014715	
231929_at	-2.25	-1.75			
220103_s_at	-2.28	-9.75	mitochondrial ribosomal protein S18C	NM_016067	MRPS18C
217537_x_at	-2.29	-4.05			
237494_at	-2.32	-2.10			
1569830_at	-2.35	-1.93	protein tyrosine phosphatase,	NM_002838 ///	PTPRC

222558_at	*-2.38*	*-1.89*		*NM_018170*	
215641_at	*-2.39*	*-1.80*	*SEC24 related gene family, member D (S. cerevisiae)*	*NM_014822*	*SEC24D*
1558345_a_at	*-2.39*	*-1.82*			
219944_at	*-2.40*	*-1.79*	*CAP-GLY domain containing linker protein family, member 4*	*NM_024692*	*CLIP4*
242868_at	*-2.41*	*-1.87*	*endothelial PAS domain protein 1*	*NM_001430*	*EPAS1*
1552425_a_at	*-2.42*	*-1.93*	*kelch-like 10 (Drosophila)*	*NM_152467*	*KLHL10*
235386_at	*-2.44*	*-1.82*			
1554170_a_at	*-2.58*	*-1.78*		*NM_203397*	
1561133_at	*-2.61*	*-2.16*	*SAR1 gene homolog B (S. cerevisiae)*	*NM_001033503 /// NM_016103*	*SAR1B*
206390_x_at	*-2.62*	*-5.78*	*platelet factor 4 (chemokine (C-X-C motif) ligand 4)*	*NM_002619*	*PF4*
235869_at	*-2.62*	*-10.07*	*short coiled-coil protein*	*NM_032547*	*SCOC*
206618_at	*-2.62*	*-1.77*	*interleukin 18 receptor 1*	*NM_003855*	*IL18R1*
238439_at	*-2.69*	*-1.75*	*ankyrin repeat domain 22*	*NM_144590*	*ANKRD22*
239351_at	*-2.69*	*-1.95*			
204666_s_at	*-2.71*	*-2.41*	*cold shock domain containing*	*NM_025073*	*CSDE1*
1569676_at	*-2.78*	*-2.19*	*torsin A interacting protein 2*	*NM_145034*	*TOR1AIP2*
237664_at	*-2.79*	*-1.97*	*protein tyrosine phosphatase-like (proline instead of catalytic arginine), member A*	*NM_014241*	*PTPLA*
1564331_at	*-2.80*	*-3.86*		*XM_091914*	
1553940_a_at	*-2.81*	*-3.29*	*leucine rich repeat containing 28*	*NM_144598*	*LRRC28*
214294_at	*-2.85*	*-3.63*			
236223_s_at	*-2.86*	*-2.54*	*Ras-like without CAAX 1*	*NM_006912*	*RIT1*
1558983_at	*-2.88*	*-1.87*	*leucine rich repeat containing 41*	*NM_006369*	*LRRC41*
229316_at	*-2.90*	*-6.03*	*chromosome 19 open reading frame 50*	*NM_024069*	*C19orf50*
201108_s_at	*-2.97*	*-2.06*	*thrombospondin 1*	*NM_003246*	*THBS1*
212641_at	*-3.00*	*-1.78*	*human immunodeficiency virus type I enhancer binding protein 2*	*NM_006734*	*HIVEP2*
234071_at	*-3.06*	*-2.97*	*DEP domain containing 6*	*NM_022783*	*DEPDC6*
1558208_at	*-3.16*	*-3.16*	*TAR DNA binding protein*	*NM_007375*	*TARDBP*
206537_at	*-3.38*	*-2.51*			
231997_at	*-3.51*	*-1.89*	*tubulin folding cofactor E-like*	*NM_152715*	*TBCEL*

1564474_at	-3.54	-2.89		XM_211988 /// XM_933335 /// XM_933337 /// XM_933341 /// XM_933350 /// XM_933356 /// XM_933360 /// XM_933363 /// XM_943735 /// XM_943738 /// XM_943741 /// XM_943744 /// XM_943747 /// XM_943750 /// XM_943754 /// XM_943757	
213260_at	-3.78	-1.94			
230422_at	-3.97	-16.25	formyl peptide receptor-like 2	NM_002030	FPRL2
231992_x_at	-4.34	-1.80		NR_002933	
205624_at	-4.41	-2.01	carboxypeptidase A3 (mast cell)	NM_001870	CPA3
243886_at	-4.46	-2.18		NM_001017927	
229485_x_at	-4.53	-2.74	shisa homolog 3 (Xenopus laevis)	XM_496688 /// XM_941105	SHISA3
1566902_at	-4.69	-2.00	low density lipoprotein receptor-related protein 8, apolipoprotein e receptor	NM_001018054 /// NM_004631 /// NM_017522 /// NM_033300	LRP8
234897_s_at	-4.94	-4.81	chromosome 6 open reading frame 21	NM_001003693	C6orf21
1554309_at	-5.03	-2.48	eukaryotic translation initiation	NM_003760	EIF4G3
207125_at	-5.41	-2.35	zinc finger protein 225	NM_013362	ZNF225
1552734_at	-5.42	-2.96		NM_152756	
217552_x_at	-5.56	-2.11	complement component (3b/4b) receptor 1 (Knops blood group)	NM_000573 /// NM_000651	CR1
233416_at	-5.88	-3.09			
204654_s_at	-6.13	-2.02	transcription factor AP-2 alpha (activating enhancer binding protein 2 alpha)	NM_001032280 /// NM_001042425 /// NM_003220	TFAP2A
242170_at	-6.26	-3.72	zinc finger protein 154		ZNF154
215124_at	-6.33	-13.23	zinc finger protein 550	NM_001039654	ZNF550
236344_at	-7.20	-2.14			
240845_at	-7.21	-2.66	ecotropic viral integration site 5	NM_005665	EVI5

207973_x_at	*-8.08*	*-13.98*	*acrosomal vesicle protein 1*	*NM_001612 /// NM_020069 /// NM_020107 /// NM_020108 /// NM_020109 /// NM_020110 /// NM_020111 /// NM_020113 /// NM_020115*	*ACRV1*
1553020_at	*-8.53*	*-1.86*	*Smith-Magenis syndrome chromosome region, candidate 5*		*SMCR5*
222722_at	*-9.74*	*-6.33*	*osteoglycin*	*NM_014057 /// NM_024416 /// NM_033014*	*OGN*
220811_at	*-11.90*	*-2.46*	*proteoglycan 3*	*NM_006093*	*PRG3*
239381_at	*-11.93*	*-1.79*	*kallikrein-related peptidase 7*	*NM_005046 /// NM_139277*	*KLK7*
1559623_at	*-12.13*	*-5.83*	*chromosome 11 open reading frame 54*	*NM_014039*	*C11orf54*
217687_at	*-13.40*	*-5.68*	*adenylate cyclase 2 (brain)*	*NM_020546*	*ADCY2*
1564760_at	*-15.94*	*-5.18*			
219788_at	*-18.54*	*-10.56*	*paired immunoglobin-like type*	*NM_013439 ///*	*PILRA*
233051_at	*-31.12*	*-4.00*	*SLIT and NTRK-like family, member 2*	*NM_032539*	*SLITRK2*
