# Supplementary figures and images for: Dissection of the Transformation of Primary Human Hematopoietic Cells by the Oncogene NUP98-HOXA9
Source: PLoS One. 2009 Aug 21;4(8):e6719. doi: 10.1371/journal.pone.0006719 (PMC2725295; doi:10.1371/journal.pone.0006719)

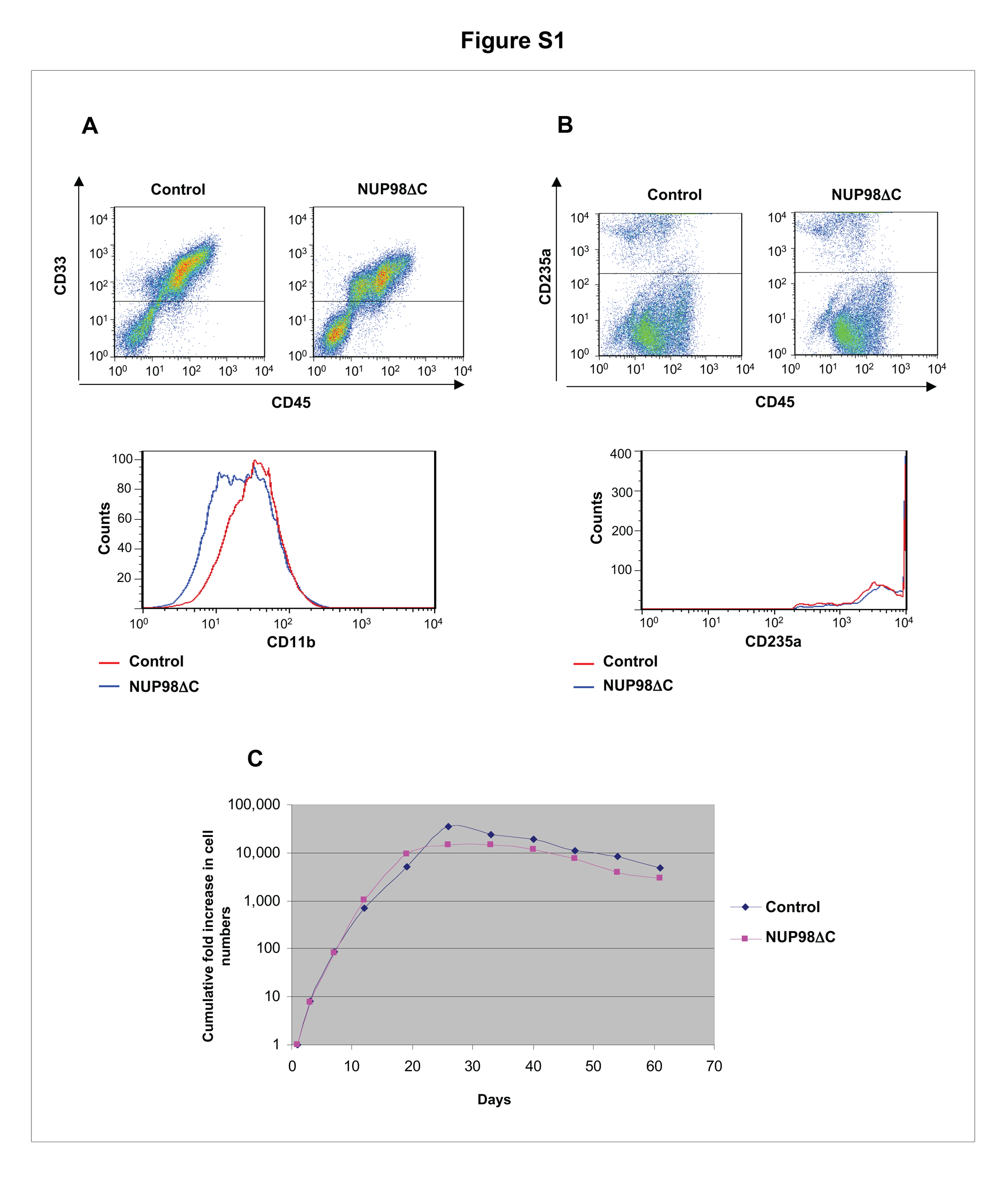

Supplement: Figure S1 — The FG repeat region of NUP98-HOXA9 has a partial effect on differentiation. The NUP98 portion of NUP98-HOXA9 (NUP98ΔC) was subcloned into the MSCV-IRES-GFP vector. Primary human CD34+ cells were retrovirally transduced with either control MSCV-IRES-GFP vector or vector expressing NUP98ΔC, and were sorted for GFP positivity. (A) Flow cytometry for myeloid differentiation: Cells from CFC plates were harvested and stained with CD45, CD33 and CD11b; the CD33+ gate was plotted on a histogram to show CD11b expression compared to control (lower panel). The data show a mild decrease in the number of myeloid cells with shift to immaturity in cells expressing NUP98ΔC. (B) Flow cytometry for erythroid differentiation: Cells from CFC plates were harvested and stained with antibodies to CD45 and CD235a. The CD235a+ gate was plotted on a histogram (lower panel) to show the expression of CD235a relative to control cells. (C) Sorted cells were grown in liquid culture in the presence of cytokines and the cumulative fold increase in cell numbers compared to day 0 is plotted on a logarithmic scale against time. (0.88 MB TIF) [file pone.0006719.s006.tif]
